# Involvement of *N4BP2L1*, *PLEKHA4*, and *BEGAIN* genes in breast cancer and muscle cell development

**DOI:** 10.3389/fcell.2024.1295403

**Published:** 2024-05-24

**Authors:** Hassan Dastsooz, Francesca Anselmi, Andrea Lauria, Chiara Cicconetti, Valentina Proserpio, Elham Mohammadisoleimani, Zahra Firoozi, Yaser Mansoori, Hamed Haghi-Aminjan, Livia Caizzi, Salvatore Oliviero

**Affiliations:** ^1^ Department of Life Sciences and Systems Biology, University of Turin, Turin, Italy; ^2^ IIGM-Italian Institute for Genomic Medicine, IRCCS, Candiolo, TO, Italy; ^3^ Candiolo Cancer Institute, FPO-IRCCS, Candiolo Cancer (IT), Torino, Italy; ^4^ Department of Medical Biotechnology, Fasa University of Medical Sciences, Fasa, Iran; ^5^ Department of Medical Genetics, Fasa University of Medical Sciences, Fasa, Iran; ^6^ Noncommunicable Diseases Research Center, Fasa University of Medical Sciences, Fasa, Iran; ^7^ Pharmaceutical Sciences Research Center, Ardabil University of Medical Sciences, Ardabil, Iran

**Keywords:** breast cancer, muscle cells, myoepithelial cells, the cancer genome atlas, breast cancer, tumor suppressor, biomarker

## Abstract

Patients with breast cancer show altered expression of genes within the pectoralis major skeletal muscle cells of the breast. Through analyses of The Cancer Genome Atlas (TCGA)-breast cancer (BRCA), we identified three previously uncharacterized putative novel tumor suppressor genes expressed in normal muscle cells, whose expression was downregulated in breast tumors. We found that NEDD4 binding protein 2-like 1 (*N4BP2L1*), pleckstrin homology domain-containing family A member 4 (*PLEKHA4*), and brain-enriched guanylate kinase-associated protein (*BEGAIN*) that are normally highly expressed in breast myoepithelial cells and smooth muscle cells were significantly downregulated in breast tumor tissues of a cohort of 50 patients with this cancer. Our data revealed that the low expression of *PLEKHA4* in patients with menopause below 50 years correlated with a higher risk of breast cancer. Moreover, we identified *N4BP2L1* and *BEGAIN* as potential biomarkers of HER2-positive breast cancer. Furthermore, low *BEGAIN* expression in breast cancer patients with blood fat, heart problems, and diabetes correlated with a higher risk of this cancer. In addition, protein and RNA expression analysis of TCGA-BRCA revealed *N4BP2L1* as a promising diagnostic protein biomarker in breast cancer. In addition, the *in silico* data of scRNA-seq showed high expression of these genes in several cell types of normal breast tissue, including breast myoepithelial cells and smooth muscle cells. Thus, our results suggest their possible tumor-suppressive function in breast cancer and muscle development.

## 1 Introduction

Breast cancer is among the most common cancers and a leading cause of cancer-related deaths among women in the world (IARC, 2022; “Statistics on preventable cancers” [Internet]; Cancer Research, 2015 [cited 2020 June 15]; Zemni et al., 2022). Currently, we are experiencing a rapid increase in new cases (Shiovitz and Korde, 2015; Giaquinto et al., 2022; Canadian Cancer Statistics, 2023; Kratzer et al., 2023). Its incidence has been continuously growing despite screening for some genes reported to be correlated with the progression of the disease, including BRCA1 and 2 DNA repair associated (*BRCA1* and *BRCA2*), phosphatase and tensin homolog (*PTEN*), ATM serine/threonine kinase (*ATM*), partner and localizer of BRCA2 (*PALB2*), and checkpoint kinase 2 (*CHEK2*) (Shiovitz and Korde, 2015). Considerably, many patients diagnosed with breast cancer experience metastasis and recurrence (Luo et al., 2018; Giaquinto et al., 2022) even after successful surgical resections and therapies (Siegel et al., 2017). Although several potential biomarkers involved in breast cancer development and metastasis have been reported, it is essential to deeply identify the pathways and processes underlying the progression of breast cancer and new prognostic and diagnostic RNA and protein biomarkers correlated with this cancer.

The pectoralis, a type of skeletal muscle, attaches the front of the chest wall to the upper extremities. On each side of the breastbone, two such muscles are present, namely, the pectoralis major and pectoralis minor (Sanchez et al., 2014). Understanding the tumor involvement of the pectoralis muscle in breast cancer before the surgical operation may change the management of surgery (Harris et al., 1992; Lagios, 1992; DiBiase et al., 1998; Obedian and Haffty, 2000; Park et al., 2000; Lee et al., 2014).

The role of mammary myoepithelial cells in breast tumors remains largely understood. Nonetheless, recent studies have suggested that these cells may regulate the transition from non-invasive to invasive cancer and the activity of stem cells. It has been reported that patients with pectoralis muscle invasion have a worse prognosis (Orel et al., 1994; Kerslake et al., 1995; Kazama et al., 2005; Sanchez et al., 2014; Mango et al., 2015; Mango et al., 2015; Bohlen et al., 2018; Pasquero et al., 2020; Myers et al., 2021). A study conducted by the E. Pistilli group focused on individuals with early-stage non-metastatic breast tumors. They found that there were changes in the expression of genes associated with muscle protein homeostasis in the pectoralis muscle of these patients (Bohlen et al., 2018). RNA sequencing (RNA-seq) on biopsies of skeletal muscle obtained from breast tumors compared with biopsies from individuals without cancer demonstrated a significant decrease in the levels of gene expression inside the breast tumor tissues in comparison to the non-tumor samples. They observed compromised canonical pathways essential for oxidative phosphorylation and defective mitochondrial function in the pectoralis muscle of their breast tumor patients. Thus, this suggested that skeletal muscle exhibited a comprehensive response to the growth of breast tumors. Tumor suppressors are persistently expressed in all or almost all myoepithelial cells in the normal ductal system (Man and Sang, 2004; Li and Man, 2009; Xu et al., 2009). In certain instances, cytoplasmic expression of *TP63* in the corresponding epithelial cells, representing more aggressiveness and invasiveness, was connected with the absence of *TP63* expression in myoepithelial cells (Zou et al., 1994; Barbareschi et al., 2001; Man et al., 2005; Man and Nieburgs, 2006; Xu et al., 2009; Hsiao et al., 2010; Man, 2010).

The goal of the current study is to investigate differential mRNA expression in patients with breast invasive carcinoma (BRCA) extracted from The Cancer Genome Atlas (TCGA). This involves univariate survival analysis, selection of uncharacterized genes showing downregulation and significant association with overall survival (OS) in BRCA patients as potential tumor suppressor genes, validation using quantitative reverse transcriptase polymerase chain reaction (RT-qPCR), *in silico* analysis of their gene set enrichment analysis (GSEA) using Enrichr [11], single-cell RNA sequencing (scRNA-seq) from the breast tissue and muscle cells during developmental stages, assessment of total RNA and protein expression in normal and breast cancer tissues, and evaluation of differential expression in various patients’ statuses of TCGA-BRCA.

Through database analyses of breast tumors, we identified new putative tumor-suppressive genes, including NEDD4 binding protein 2-like 1 (*N4BP2L1*), pleckstrin homology domain-containing family A member 4 (*PLEKHA4*), and brain-enriched guanylate kinase-associated protein (*BEGAIN*), and validated their decreased expression by RT-qPCR in our cohort of 50 patients with breast tumors and the expression in the muscle cells by RT-qPCR on muscle cells differentiated from H9 human embryonic stem cells (hESCs).

## 2 Materials and methods

**FIGURE 1:**
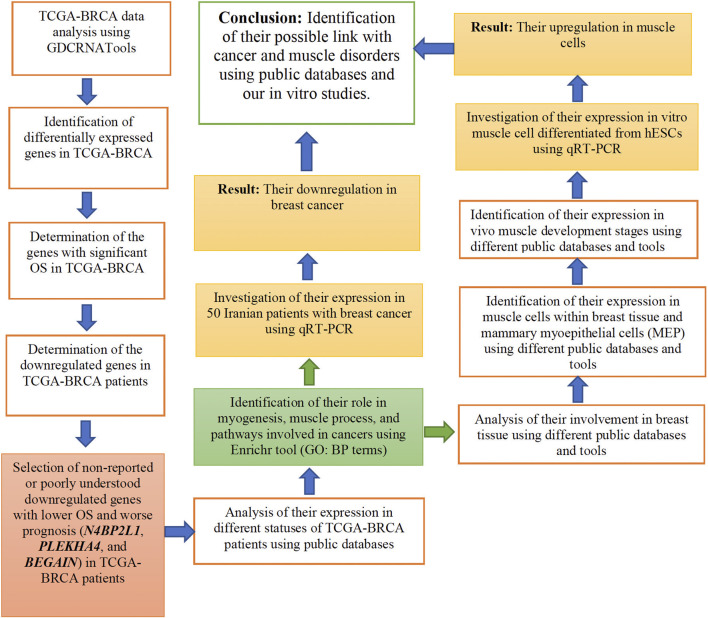
Flow chart of the research process used in the current study.

In our study, we considered experimental methods and bioinformatic analyses, as described in Figure 1. In each of the following sections, we explain them in detail.

### 2.1 Gene expression analysis in TCGA-BRCA

#### 2.1.1 Collection of TCGA-BRCA data and its differential mRNA expression levels using GDCRNATools

We used the R/Bioconductor package GDCRNATools (Li et al., 2018) to investigate, first, the differential gene expression of patients with BRCA from TCGA. The gene expression data from tumors and their matched adjacent non-tumor tissue were analyzed as described previously (Li et al., 2018). The following functions were applied to the TCGA-BRCA dataset: gdcParseMetadata, gdcFilterDuplicate, gdcFilterSampleType, gdcVoomNormalization, gdcDEAnalysis, and gdcDEReport functions, as reported in a previous study (Dastsooz et al., 2021). In our analysis, we considered 1,222 RNA sequencing data and 1,097 clinical data from the TCGA-BRCA dataset. In the next step, the univariate survival analysis was carried out by applying the gdcSurvivalAnalysis function from the same package (Kaplan–Meier division of patients into high- and low-expression groups). Among the differentially expressed genes, we looked for those with significant OS, and finally, we selected those downregulated genes (as possible tumor suppressor genes) that have not been reported or poorly understood in BRCA patients for further analyses.

#### 2.1.2 Expression analysis of the selected TCGA-BRCA genes across the different patients’ statuses and other TCGA cancers using TACCO and UALCAN web resources

To strengthen the possible role of three uncharacterized tumor-suppressive genes in breast cancer, we used different databases for extracting TCGA data, including the TACCO web server (http://tacco.life.nctu.edu.tw) (Chou et al., 2019) for the investigation of gene expression profiling across all TCGA cancers and UALCAN (https://ualcan.path.uab.edu/index.html) (Chandrashekar et al., 2017; Chandrashekar et al., 2022) for the analysis of gene expression in RNA and protein levels in different statuses of patients with breast tumor, including tumor stages, subclasses, histologic subtypes, and nodal metastasis. UALCAN used data from the Clinical Proteomic Tumor Analysis Consortium (CPTAC, https://proteomics.cancer.gov/programs/cptac) (Edwards et al., 2015) and International Cancer Proteogenome Consortium (ICPC, https://icpc.cancer.gov/portal/) datasets.

### 2.2 Gene ontology analysis of *N4BP2L1*, *PLEKHA4*, and *BEGAIN* using Enrichr

We used the Enrichr web-based tool (Kuleshov et al., 2016) to search the “biological process” sub-ontology of Gene Ontology (GO: BP) for *N4BP2L1*, *PLEKHA4*, and *BEGAIN*. This tool helped analyze the link between proteins (e.g., their interactions or co-regulation associations), assuming that proteins with the same annotations (or relations) of GO: BP terms have interactions with each other or are functionally connected.

### 2.3 *N4BP2L1*, *PLEKHA4*, and *BEGAIN* mRNA expression in a cohort of Iranian patients with breast cancer

#### 2.3.1 Breast cancer samples, RNA isolation, and cDNA synthesis assay

In the current study, 50 primary tumors and their paired normal tissues adjacent to the tumor were collected from Iranian patients with breast cancer from Shahid Faghihi Hospital (Shiraz, Fars, Iran). The patients enrolled in our study did not have chemotherapy or radiotherapy before the surgical operation. Fresh tissue samples were snap-frozen in liquid nitrogen and stored at −80°C until future processing. It should be noted that all tumors were diagnosed and validated by histopathological examination. We collected written informed consent from all patients before the start of our studies, and the Research Ethics Committee of the Fasa University of Medical Sciences (ethical code: IR FUMS. REC.1397.143) provided ethical approval for the study, thereby granting the necessary authorization for the research to be conducted adhering to established ethical guidelines and principles. The clinical and demographic data from the patients were collected using a standard questionnaire, as reported in our previous study (Mansoori et al., 2021). As shown in Supplementary Table S1, different statuses (the study variables) of our cohort were as follows: age, tumor size, estrogen receptor, progesterone receptor, HER-2 receptor, nuclear grade, histologic grade, lymph nodes metastasis, histologic type of invasive carcinoma, marital status, cut point of BMI, age of menarche, regular or irregular menstruation, age of menopause, menopausal status, history of uterine surgery, number of pregnancies, age at FFTP, breastfeeding duration, number of abortions, abortion history, family history of cancer, disease history, chest radiograph history, hormone therapy history, duration of OCP consumption, OCP consumption, vitamin D consumption, regular sleep, exercising, deodorant use, hair dye use, and cosmetics use. As shown in Supplementary Table S1, for each variable, the patients were divided into different groups, and then we investigated the correlation between each variable and the expression of the selected genes in our cohort. The experiment was carried out with the comprehension and consent of the human participants, along with a declaration that the ethical committee in charge had approved the experiments. The administration of anesthetics or surgical procedures and the provision of evidence demonstrating the implementation of all feasible measures to prevent animal distress at every stage of the experiment were ensured.

Total RNA was prepared from 50 primary tumors and their paired normal tissues adjacent to the tumor of the Iranian patients with breast cancer using TRIzol (Invitrogen, Thermo Fisher Scientific) based on the manufacturer’s recommendations. We measured their concentration using a spectrophotometer and determined their quality by agarose gel electrophoresis. Then, we performed complementary DNA (cDNA) synthesis using a Thermo Fisher Scientific ™ First Strand cDNA Synthesis Kit (Fermentas, Cat. No: K1622). All clinical data on our patients are provided in Supplementary Table S1. The RNA and clinical data from these samples have also been used in our previous study (Mansoori et al., 2021).

#### 2.3.2 RT-qPCR analysis

The real-time PCR reaction was prepared for the test, internal control samples, and negative control in duplicate using RealQ Plus 2x Master Mix Green with ${High\;ROX}^{TM}$ (Ampliqon, Cat. No: A325402-25). The RT-qPCR reaction was carried out based on the following conditions: 45 cycles of 95°C for 20 s and then 60°C for 30 s. The *B2M*, *GAPDH*, and *ACTB* genes are often employed as housekeeping genes for breast cancer studies (Gaffo et al., 2019; Vo et al., 2019). Expression stability values of endogenous control genes were calculated by the geNorm and NormFinder programs. In our study, we used the β2M housekeeping gene as an internal control (oligo sequences are given in Table 1) that is more suitable for breast tissues (McNeill et al., 2007). The ΔΔCt value (logarithmic scale, log fold-change) was considered for relative expression analysis.

**TABLE 1:** Primer sequences used for the RT-qPCR assay.

Gene symbol	Oligo sequence (5′ to 3′)
*BEGAIN*	F: AGCACTATGAGGAGGAGA
	R: AGA​TTG​CAG​TCC​TTC​CTA​T
*PLEKHA4*	F: TTCATAAGCAGGACAGCT
	R: CCC​ATC​TGG​TCT​AAT​ATT​GT
*N4BP2L1*	F: GAAACACCTCTACCTCCT
	R: CAG​GAA​GTC​AGG​ATT​GAA​C
*β2M*	F: AGA​TGA​GTA​TGC​CTG​CCG​TG
	R: GCG​GCA​TCT​TCA​AAC​CTC​CA
*MYOG*	F-MYOG-QPCR2 ATG​GAG​CTG​TAT​GAG​ACA​TC
	R-MYOG-QPCR2 ACA​CCG​ACT​TCC​TCT​TAC​A
	F-MYOG.QPCR3 GCT​GTA​TGA​GAC​ATC​CCC​CTA
	R-MYOG.QPCR3 CGA​CTT​CCT​CTT​ACA​CAC​CTT​AC
*PUM1*	PUM1-F: GGG​CAT​GGA​GCC​TCT​TCA​GTT​T
	PUM1-R: GGA​CAG​CAA​GCG​CAT​TAG​GTC​TTT
*TBXT*	F-TBXT: CCAGATCATGCTGAACTC
	R-TBXT: CTGTGATCTCCTCGTTCT
*PAX3*	F-PAX3: AAGATCCTGTGCAGGTAC
	R-PAX3: CTGATGGAACTCACTGAC

#### 2.3.3 Statistical analysis of experiments related to breast cancer samples

The mean and standard deviation were used for presenting ΔΔCt, and the median was used for fold change. ΔΔCt ‘s comparison between the tumors and adjacent non-tumor tissues was performed using the paired sample *t*-test, and the comparison of log fold changes between these samples was performed using the Mann–Whitney test. The association between the expression of these genes and clinicopathologic and demographic factors was analyzed using the Mann–Whitney and Kruskal–Wallis tests. According to the median, log fold changes were divided into two groups of high and low expressions, and the chi-square test and independent *t*-test were used to compare these groups. IBM SPSS 26 statistical software was applied for data analyses. The degree of a *p*-value less than 0.05 was defined as statistically significant.

### 2.4 Analysis of *N4BP2L1*, *PLEKHA4*, and *BEGAIN* involvement in breast tissue and muscle cells

#### 2.4.1 Expression analysis of these genes in normal public bulk RNA-seq, single-cell RNA-seq, and protein databases

We retrieved the expression of *N4BP2L1*, *PLEKHA4*, and *BEGAIN* at bulk and single-cell RNA-seq levels from the GTEx database (Uhlen et al., 2005; Consortium, 2013; Carithers and Moore, 2015; Uhlen et al., 2015; Karlsson et al., 2021) using the Human Protein Atlas (https://www.proteinatlas.org). The protein expression levels were also available as immunohistochemistry of breast tissues in the Human Protein Atlas (Uhlen et al., 2005).

#### 2.4.2 *PLEKHA4*, *N4BP2L1*, and *BEGAIN* expression across developmental stages using the Human Skeletal Muscle Atlas

In this analysis, we used the data from the Human Skeletal Muscle Atlas (https://aprilpylelab.com/datasets/) (https://doi.org/10.1016/j.stem.2020.04.017) to investigate the expression of these genes in different *in vivo* muscle developmental stages. The data contain single-cell RNA sequencing of human skeletal muscle tissues from the embryonic, fetal, and postnatal stages (Xi et al., 2020).

#### 2.4.3 *PLEKHA4*, *N4BP2L1*, and *BEGAIN* expression in mammary myoepithelial cells compared to luminal epithelial cells using GREIN tools

In another part of our study, we investigated the expression levels of *N4BP2L1*, *PLEKHA4*, and *BEGAIN* in myoepithelial (MEP) cells compared with luminal epithelial (LEP) cells isolated from primary breast organoids and also the expression pattern of recognized tumor suppressor genes, including *TP63* and *SERPINB5*, among others. Since organoids are simple tissue-engineered cell-based *in vitro* models that recapitulate many aspects of the complex structure and function of the corresponding *in vivo* tissue, we analyzed this model. The GSE182338 data were analyzed using online GREIN tools (GEO RNA-seq Experiments Interactive Navigator, http://www.ilincs.org/apps/grein/?gse=GSE182338) (Mahi et al., 2019). This web-based tool offers alternatives for exploring and analyzing GEO RNA-seq data that are easy to use. It is supported by several existing processed datasets and the back-end computational pipeline to consistently process the RNA-seq data. GSE182338 data contain transcriptional profiling of human mammary LEP and MEP cells from different samples, including the primary breast organoids. We analyzed differential expression in organoids prepared from samples with an age/risk status considered normal risk for individuals <30 years (Sayaman, 2021).

#### 2.4.4 Expression analysis of the selected genes in muscle cells

In our study, we used a protocol described by Shelton et al. (2016) to differentiate H9 hESCs into skeletal muscle cells. H9 hESCs were grown and maintained in TeSR™-E8™ (STEMCELL Technologies) in 6-well plates coated with 0.5% Corning Matrigel^®^ Growth Factor Reduced (GFR) Basement Membrane Matrix, Phenol Red-Free (Cat. No: 356231), prepared in PBS 1X, or advanced DMEM/F12 (Gibco). The cells were grown in an incubator at 37°C with 5% CO_2_ and 5% O_2_. The ES media was changed daily, and when the cells reached 70%–80% confluency with healthy pluripotent colonies, they were dissociated into single cells to form cell clusters, with 6–15 cells in each cluster on the day of differentiation.

The H9 hESCs were differentiated in a 12-well plate coated with 3% Corning Matrigel with the protocol described by Shelton, M et al. Our differentiation stages were performed for 46 days (D46, terminal stage), and we collected cells for RNA extraction and RT-qPCR at different muscle stages (D0: undifferentiated, D2: mesoderm stage, D7: somite stage, D30: skeletal muscle progenitors’ (SMPs’) stage, and D46: terminally differentiated stage). RT-qPCR for the *T* (Brachyury) gene (*TBXT*) on day 2, paired box 3 (*PAX3*) on day 7, and myogenin (*MYOG*) on day 30 (the *PUM1* gene was considered an internal reference gene, Table 1) and immunofluorescent (IF) staining for MF20 (myosin heavy chain protein) on D46 were performed to confirm the differentiation process and presence of muscle cells. Regarding the MF20 IF assay, the first antibody was mouse DSHB (Developmental Studies Hybridoma Bank) AB_2147781, and the secondary antibody was anti-mouse Alexa Fluor 488, goat Invitrogen A11001. RNA extraction was carried out by QIAzol reagent (QIAGEN), and the RNA quality was determined using the Agilent Bioanalyzer 2100 (RNA integrity number ranging from 8 to 10). We then investigated the relative expression levels of *PLEKHA4*, *N4BP2L1*, and *BEGAIN* by RT-qPCR in the muscle cells compared to hESCs.

### 2.5 Disease association analysis of *PLEKHA4*, *N4BP2L1*, and *BEGAIN* using the CTD dataset

Moreover, we used the CTD dataset (Davis et al., 2023) (https://ctdbase.org) for finding any possible association between these genes and diseases. This dataset contains curated information related to interactions between chemical compounds and the gene/protein, associations of chemicals with diseases, and genes with diseases, combined with functional and pathway data. CTD includes curated and inferred chemical–disease associations. The *curated* one is retrieved from the publications, while the *inferred* relationships are created through curated interactions between chemical compounds and genes (for example, chemical compound H has a relationship with cancer I since chemical compound H shows a curated interaction with gene L, and gene L has a curated relationship with cancer I) (Barabasi and Albert, 1999; Li and Liang, 2009; King et al., 2012).

## 3 Results

### 3.1 Differential gene expression analysis of TCGA-BRCA samples

We performed a differential gene expression analysis of normal *versus* tumor samples collected in TCGA-BRCA through the GDCRNATools package, resulting in several differentially expressed genes (Supplementary Table S2). We found that the expression of some of them was correlated with patient prognosis (Supplementary Table S3). Our analysis revealed some downregulated genes, including *N4BP2L1*, *BEGAIN*, and *PLEKHA4*, and some upregulated genes, such as *SLC35A2*, *DONSON*, *BRI3BP*, *NDUFAF6*, *C2CD4D*, and *SEZ6L2*, which have not been reported or poorly understood in breast cancer (Table 2). Considering the expression data and patient OS, we proceeded by investigating the genes with lower expression and a possible tumor-suppressive role (Table 3).

**TABLE 2:** Poorly understood proto-oncogenes and tumor suppressor genes annotated in the TCGA-BRCA dataset with significant OS.

Ensembl stable ID	Symbol	HR^a^	lower95	upper95	*p*-value
ENSG00000102100	*SLC35A2*	1,63406375	1,18565104	2,25206596	0,00251
ENSG00000105559	*PLEKHA4*	0,61182267	0,44462008	0,84190301	0,00282
ENSG00000156170	*NDUFAF6*	1,59721628	1,16097767	2,19737202	0,00469
ENSG00000183092	*BEGAIN*	0,69059614	0,50139344	0,95119519	0,0225
ENSG00000174938	*SEZ6L2*	1,38749366	1,00812272	1,9096273	0,0443
ENSG00000159147	*DONSON*	1,38587909	1,00734744	1,90665185	0,0464
ENSG00000184992	*BRI3BP*	1,38031826	1,00242378	1,90067168	0,0472
ENSG00000139597	*N4BP2L1*	0,72531056	0,52635472	0,99946935	0,0472
ENSG00000225556	*C2CD4D*	0,7264943	0,52788767	0,99982251	0,0483

^a^ Hazard ratio.

**TABLE 3:** Downregulated genes with possible new tumor suppressor function in TCGA-BRCA.

Ensembl stable ID	Symbol	logFC	AvgExpr	t	*p*-value	FDR	B
ENSG00000183092	*BEGAIN*	−1,406	0.173	−11.2	8,85E-28	4,39E-27	51.96
ENSG00000139597	*N4BP2L1*	−1,462	3,660	−22,055	7,72E-91	2,84E-89	196.18
ENSG00000105559	*PLEKHA4*	−1,225	4,638	−11.16	1,40E-27	6,87E-27	51.05

t, Student’s t-Test; B, B-statistic.

### 3.2 *N4BP2L1*, *PLEKHA4,* and *BEGAIN* expression across all TCGA cancers and different statuses of TCGA-BRCA patients

We retrieved TCGA data from all tumors to show the possible involvement of *N4BP2L1*, *PLEKHA4,* and *BEGAIN* not only in breast cancer but also in other cancers. Using different databases to extract TCGA data, we found that these genes were significantly downregulated in several tumors, with the most significant difference in breast cancer (Supplementary Table S4). Using the TACCO web server, it was found that *N4BP2L1* showed lower expression in cancers of the breast, lung, liver, uterine, head–neck, stomach, prostate, colon, bladder, kidney (chromophobe), brain (glioblastoma multiforme), pancreas, cervix, and endometrium (Supplementary Table S4). Regarding *PLEKHA4*, it revealed decreased expression levels in tumors of the breast, liver, lung, kidney (chromophobe), uterine, bladder, and head–neck (Supplementary Table S4). *BEGAIN* showed downregulation in cancers of the breast, uterine, stomach, prostate, colon, bladder, thyroid, kidney (kidney renal papillary cell carcinoma and chromophobe), and brain (Glioblastoma multiforme) (Supplementary Table S4). These data, representing the downregulation of these genes in several cancers, may indicate their important roles in tumorigenesis. Therefore, we analyzed their expression in different patients’ statuses in TCGA-BRCA.

In the next step, we looked for their expression in different tumor stages of TCGA-BRCA. As shown in Supplementary Figure S1, panel 1, all three genes, *N4BP2L1*, *PLEKHA4*, and *BEGAIN*, had a significantly decreased expression in all tumor stages (1, 2, 3, and 4) compared to their matched normal tissues. Regarding *N4BP2L1*, the later tumor stage 4 also showed significantly lower expression compared to stage 1 (*p*-value: 0.0157). Concerning *BEGAIN,* tumor stage 4 revealed significantly more downregulation compared to stages 1, 2, and 3 (*p*-values: 0.00066, 0.0037, and 0.0022, respectively). Stage IV (4) tumors are usually considered metastatic forms. Therefore, these results represent their more probable role in tumor metastasis.

We then analyzed their expression in the three main breast cancer subclasses, and these genes had a significant downregulation in all the subclasses compared to normal tissues, including luminal types (*N4BP2L1*: *p*-value <1E-12; *PLEKHA4*: *p*-value = 1.62E-12; and *BEGAIN*: *p*-value = 1.49-E08), HER2-positive (*N4BP2L1*: *p*-value = 1.62E-12; *PLEKHA4*: *p*-value = 1.62E-12; and *BEGAIN*: *p*-value = 4.3-E15), and triple-negative (*N4BP2L1*: *p*-value <1E-12; *PLEKHA4*: *p*-value = 5.7E-10; and *BEGAIN*: *p*-value = 0.0011) (Supplementary Figure S1; panel 2). It is worth noting that their expression in the HER2-positive subclass was lower than in the other two subclasses, followed by the triple-negative and luminal categories. These data may represent the possible involvement of these genes in all three different subclasses of breast cancer, mainly the HER2-positive one.

After that, we evaluated the expression of these genes in all triple-negative subclasses of TCGA-BRCA, and they revealed downregulation in most of these subclasses compared to normal tissues (Supplementary Figure S1; panel 3). Our results found that *N4BP2L1* had lower expression in all subclasses, *PLEKHA4* had decreased expression in almost all, except for TNBC-basal-like 2 (BL2) and -unspecified (UNS), and *BEGAIN* had downregulation in TNBC-basal-like 1 (BL1), TNBC-immunomodulatory (IM), and TNBC-luminal androgen receptor (LAR). As seen in Supplementary Figure S1, panel 3, *N4BP2L1* and *PLEKHA4* genes also had significant downregulation in TNBC-MSL (TNBC mesenchymal stem-like) and mainly TNBC-M (TNBC mesenchymal), which originate from the mesoderm involved in the muscle differentiation process.

Next, we investigated *N4BP2L1*, *PLEKHA4*, and *BEGAIN* expression in TCGA-BRCA based on histologic subtypes (IDC: infiltrating ductal carcinoma, mixed histology, medullary carcinoma, INOS: infiltrating carcinoma NOS, ILC: infiltrating lobular carcinoma, mucinous carcinoma, and metaplastic carcinoma). As seen in Supplementary Figure S1, panel 4, the data showed a significant *N4BP2L1* downregulation in all subtypes (regarding INOS, it represented NA, not applicable, for the three genes due to its low sample size); significantly, *PLEKHA4* showed lower expression in all subtypes except for metaplastic ones, and significantly decreased expression of *BEGAIN* was found only in IDC, ILC, and mixed subtypes compared to normal tissues. As shown in this panel, several breast cancer subtypes revealed significant downregulation of these genes compared to normal tissue as well as each other, indicating their possible role in specific subtypes. Based on these findings, these genes may be involved in different histologic breast tumor subtypes, mainly for *N4BP2L1* and *PLEKHA4*, with differential expression in most of them. Since *N4BP2L1* also had significantly lower expression in metaplastic breast cancer (*p*-value = 1.7E-12), it may indicate its involvement in breast cancer metastasis, as mentioned in the earlier sections.

We also analyzed the possible involvement of these genes in the nodal metastasis statuses of TCGA-BRCA (N0: no regional lymph node metastasis, N1: metastases in 1–3 axillary lymph nodes, N2: metastases in 4–9 axillary lymph nodes, and N3: metastases in 10 or more axillary lymph nodes). The TCGA data showed their significant downregulation in all statuses compared to their normal counterparts (Supplementary Figure S1; panel 5). *PLEKHA4* and *BEGAIN* revealed significantly decreased expression not only in N0 compared to the normal but also in other comparisons as follows: *PLEKHA4* lower expression in N1 (*p*-value = 0.038) and N2 (*p*-value = 0.017) compared to N3, and *BEGAIN* downregulation in N2 compared to N0 (*p*-value = 0.0021), N1 (*p*-value = 0.013), and N3 (*p*-value = 0.037). Since studies have shown that non-regional lymph node (NRLN) metastases can be considered in the prognosis evaluation and clinical management of cancers (Pond et al., 2014; James et al., 2015), assessing these genes together may be considered a prognostic biomarker set. This is because the downregulation of *N4BP2L1* is not changed in N1, N2, and N3 compared to N0 regional lymph node metastasis, while others have alterations. Altogether, these data may represent the possible role of these genes in breast cancer metastasis (Supplementary Figure S1; panel 5).

### 3.3 GO-associated processes of *N4BP2L1*, *BEGAIN*, and *PLEKHA4*

**FIGURE 2:**
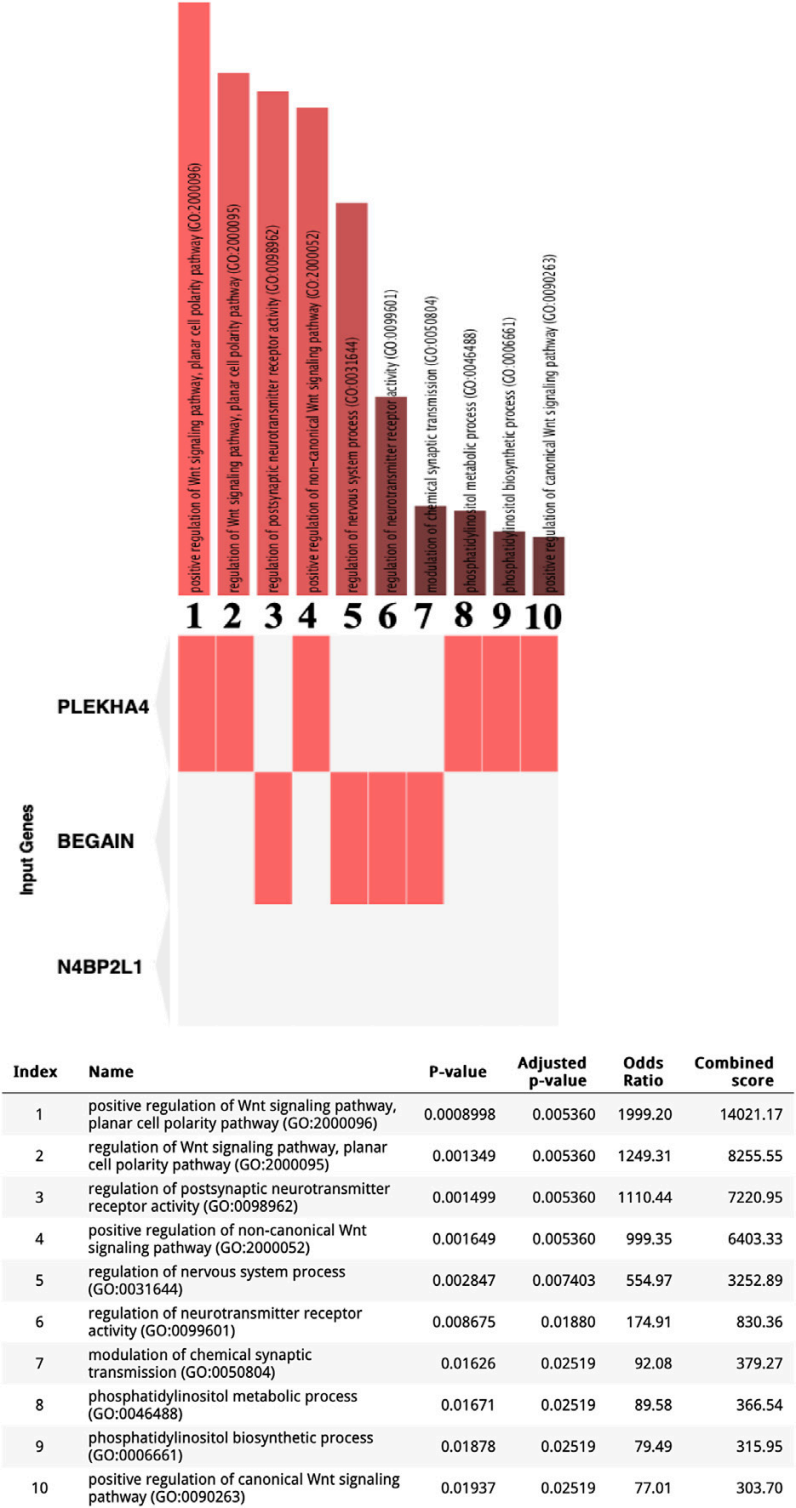
Enrichr GO: BP terms for three uncharacterized tumor suppressor genes in the TCGA-BRCA dataset. Significant GO: BP terms were only found for *BEGAIN* and *PLEKHA4*.

We interrogated the Enrichr web tool with our uncharacterized tumor suppressor genes and found their involvement in different biological processes. Interestingly, these genes may have a direct or indirect role in myogenesis based on Enrichr GO: BP (Figure 2). In particular, *PLEKHA4* is involved in the regulation of the Wnt signaling pathway, which is critical for the induction of mesoderm and muscle lineage. The significant Enrichr GO: BP terms associated with *PLEKHA4* included the positive regulation of the Wnt signaling pathway, planar cell polarity pathway (GO:2000096), regulation of the Wnt signaling pathway, planar cell polarity pathway (GO:2000095), positive regulation of the non-canonical Wnt signaling pathway (GO:2000052), positive regulation of the canonical Wnt signaling pathway (GO:0090263), phosphatidylinositol metabolic process (GO:0046488), and phosphatidylinositol biosynthetic process (GO:0006661) (Figure 2). *BEGAIN* can also be involved in muscle differentiation since it has a function in the regulation of postsynaptic neurotransmitter receptor activity (GO:0098962 and GO:0099601) and the modulation of chemical synaptic transmission (GO:0050804) (Figure 2), with an important role in neuromuscular junctions (NMJs), a cellular synapse between a motor neuron and a skeletal muscle fiber (Vilmont et al., 2016). It also plays a role in the regulation of the nervous system process (GO:0031644) (Figure 2). Regarding *N4BP2L1,* although Enrichr did not reveal any biological processes for this gene (Figure 2), through literature mining (https://www.genecards.org) (Stelzer et al., 2016), we found that this gene is reported to be involved in inflammatory leiomyosarcoma, which originated from muscle tissues. Therefore, for the first time, we propose its role in the muscle process and also breast cancer.

### 3.4 *N4BP2L1*, *BEGAIN*, and *PLEKHA4* expression in 50 Iranian patients with breast cancer and their correlations with the patients’ statuses

**FIGURE 3:**
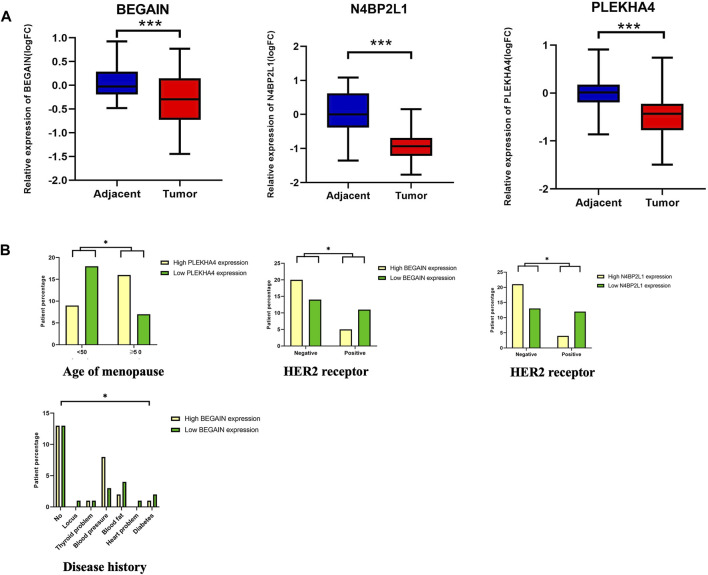
**(A)** Downregulation of *N4BP2L1*, *BEGAIN*, and *PLEKHA4* in 50 patients using RT-qPCR. **(B)** Correlation between *PLEKHA4* expression and the percentage of patients with breast cancer at different ages of menopause (Mann–Whitney test), a correlation between *N4BP2L1* and *BEGAIN* expression (Kruskal–Wallis test) and HER2-positive (Mann–Whitney test), and a correlation between *BEGAIN* expression and disease history of our patients with breast cancer. The box and whisker plot (10–90 percentile) of the relative expression levels of genes (ns, *p* > 0.05; *,*p* ≤ 0.05; **,*p* ≤ 0.01; ***,*p* ˂0.001) were plotted using GraphPad Prism 9.3.1.

In the next step, we considered the uncharacterized genes with a low OS and a lower expression in TCGA-BRCA tumor samples compared to their normal counterparts in the dataset (Tables 2 and 3). We confirmed the downregulation of *N4BP2L1*, *BEGAIN*, and *PLEKHA4* in our cohort of 50 patients with breast cancer using RT-qPCR (Figure 3A). As described in Supplementary Table S1, for each variable, our cases were divided into different groups, and then we analyzed their correlation with the mRNA expression of the selected genes.

When we compared the relative expression of these genes, grouping the patients according to the age of the onset of menopause, *PLEKHA4* revealed a significant difference (Mann–Whitney test, *p*-value <0.05) (Figure 3B; Supplementary Table S1, Panel A). Our results showed that 69.6% of patients with an age of menopause onset below 50 years or after were in the higher *PLEKHA4* expression group, while 30.4% of the patients were in the lower *PLEKHA4* expression group. However, for patients with an age of menopause onset before 50 years, 66.7% of them were in the lower *PLEKHA4* expression group, and 33.3% of the patients were in the higher *PLEKHA4* expression group. Therefore, patients with an age of menopause onset below 50 who had lower expression of *PLEKHA4* were at a higher risk of breast cancer, and this gene could be correlated with the age of menopause in patients with breast cancer. Moreover, our study showed that 75% and 69% of breast cancer patients who were HER2-positive had lower *N4BP2L1* and *BEGAIN* expressions, respectively. Therefore, these two genes can be proposed as potential biomarkers of HER2-positive breast cancer (Supplementary Table S1; panels B and C). Furthermore, breast cancer patients with high blood fat, heart problems, diabetes, and locus together with low *BEGAIN* expression were at a higher risk of this cancer (Figure 3B; Supplementary Table S1; panel C).

### 3.5 N4BP2L1, PLEKHA4, and BEGAIN protein expression in TCGA-BRCA and other datasets

We also looked for the protein expression of these genes in TCGA breast cancer tissues. As shown in Supplementary Figure S2, panel A, N4BP2L1 showed weak expression in most of the samples (12 out of 18, 66%). However, PLEKHA4 had weak expression only in one sample out of 30 samples (Supplementary Figure S2; panel B), and BEGAIN protein revealed moderate expression in all 20 samples tested (Supplementary Figure S2; panel C). In addition to the protein expression of these genes in TCGA-BRCA, we investigated their expression in the CPTAC using UALCAN. As seen in Supplementary Figure S2, panel D, the N4BP2L1 protein showed highly significant lower expression (*p*-value: 4.56E-05) in 125 primary breast tumors compared to their matched normal tissues. Moreover, as shown in Supplementary Figure S2, panel E, its protein downregulation was found in breast cancer stages 1, 2, and 3; tumor subclasses of luminal, HER2-positive, and TNBC; histological subtypes of IDC and mixed ones; pathways of *HIPPO*, *WNT*, *mTOR*, *RTK*, *P53*/*RB*, and other ones; *SWI-SNF* complex status of patients; *MYC*/*MYCN* altered condition; and chromatin modifier altered patients’ statuses. Therefore, based on these protein expression data, the N4BP2L1 protein may be considered a promising protein biomarker in breast cancer.

### 3.6 *N4BP2L1*, *BEGAIN*, and *PLEKHA4* expression at the normal single-cell RNA level and total RNA and protein expression levels

In the next step, we investigated the bulk and single-cell RNA expression levels of these genes and their protein abundance by interrogating the Human Protein Atlas (https://www.proteinatlas.org) and GTEx (https://gtexportal.org/home/) databases. In particular, single-cell RNA data could help reveal which cell types within breast tissue express these genes, giving important hints for their function elucidation. We selected normal breast tissues from the GTEx database and observed that *N4BP2L1* had an RNA expression of 25.7 normalized transcripts per million (nTPM) in GTEx. The Human Protein Atlas database reports a medium protein immunostaining score in myoepithelial and glandular cells (Supplementary Figure S2; panel F). *PLEKHA4* RNA expression was 31.7 nTPM in GTEx, and its immunostaining score is high in myoepithelial and medium in glandular cells (Supplementary Figure S2; panel G). *BEGAIN* RNA expression showed 1.7 nTPM in GTEx, and its protein expression analysis is pending and has not been performed yet. These data revealed that these genes are expressed in the breast tissue in bulk RNA and by protein immunohistochemistry.

**FIGURE 4:**
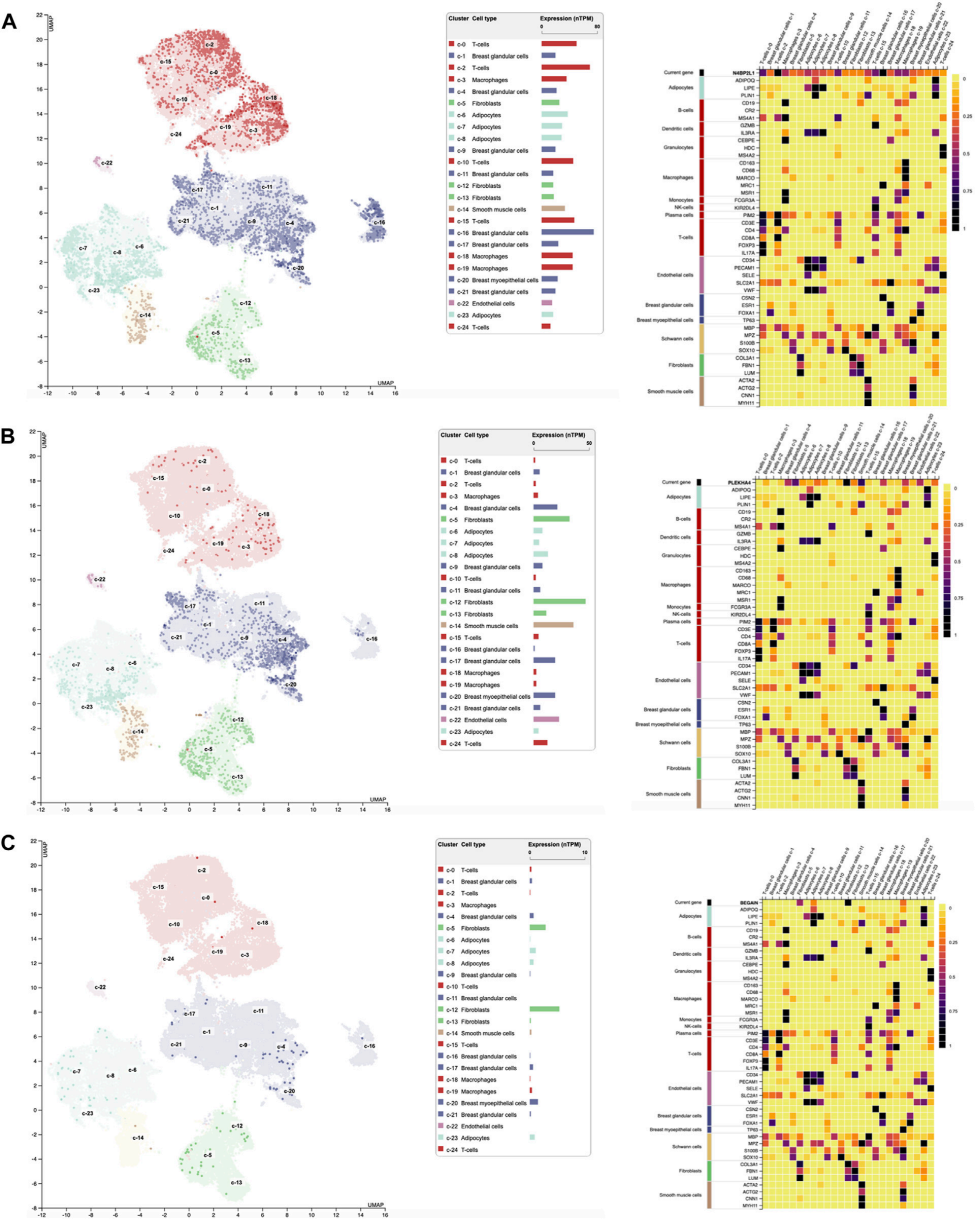
Single-cell RNA expression of breast tissue extracted from the Human Protein Atlas; **(A–C)** *N4BP2L1*, *PLEKHA4,* and *BEGAIN* expressions, respectively, in different cell types of breast tissue.

Single-cell RNA sequencing extracted from the Human Protein Atlas revealed the different expression patterns of these genes in different cell types grouped into 25 clusters of breast tissues as follows (Figure 4): regarding *N4BP2L1*, its higher expression was observed in clusters 16, C-16 (nTPM: 75.2, breast glandular cells), C-2 (nTPM: 69.5, T-cells), and C-0 (nTPM: 50.3, T-cells) (Figure 4A). Moreover, it had expression in breast myoepithelial cells C-20 (nTPM: 23) and smooth muscle cells C-14 (nTPM: 33.2), revealing its involvement in muscle cells. In most of the cell clusters, it showed a notable expression that can represent its role in these cells in breast tissue.

In relation to *PLEKHA4*, its higher expression was in clusters C-12 (nTPM: 47.1, fibroblasts, mesenchymal cells), C-14 (nTPM: 36.2, smooth muscle cells), and C-5 (nTPM: 32.7, fibroblasts, mesenchymal cells) (Figure 4B). Since we observed *PLEKHA4* expression in myoepithelial cells (C-20, nTPM: 19.4) and smooth muscle cells, we conclude that it may have a function in muscle cells. This gene is also expressed in other cells of breast tissue, such as endothelial cells (C-22) and breast glandular cells (such as C-4 and C-17), among others. In the previous section, our data from Enrichr GO: BP showed *PLEKHA4* involvement in the regulation of the Wnt signaling pathway (important in the mesenchymal stage); therefore, it is reliable to see its higher expression in fibroblasts, as mesenchymal cells, of breast tissues.

*BEGAIN* revealed higher expression in clusters C-12 (nTPM: 5.4, fibroblasts, mesenchymal cells), C-5 (nTPM: 2.9, fibroblasts, mesenchymal cells), and C-20 (nTPM: 1.5, myoepithelial cells). Its expression in the smooth muscle cells was 0.3 nTPM (Figure 4C). Higher expression of *BEGAIN* in fibroblasts, mesenchymal cells, and breast tissue suggests its possible role in the muscle process.

The highest expression of *PLEKHA4* and *BEGAIN* was in mesenchymal cells, which was 103 nTPM and 5.4 nTPM, respectively. However, the notable expression of *N4BP2L1* was in both mesenchymal cells (fibroblasts) and myoepithelial cells with the same level, 75.2 nTPM. These expression data can suggest their important roles in different cell types of breast tissue, mainly muscle cells and their progenitors, as found in our muscle cell differentiation data explained in the next section. Moreover, we observed the notably higher expression of *PLEKHA4* with respect to *N4BP2L1* and *BEGAIN* in smooth muscle cells in RNA single-cell sequencing data extracted from the Human Protein Atlas database, as found in our muscle cells differentiated from hESCs.

### 3.7 *PLEKHA4*, *N4BP2L1*, and *BEGAIN* expression across *in vivo* developmental stages

**FIGURE 5:**
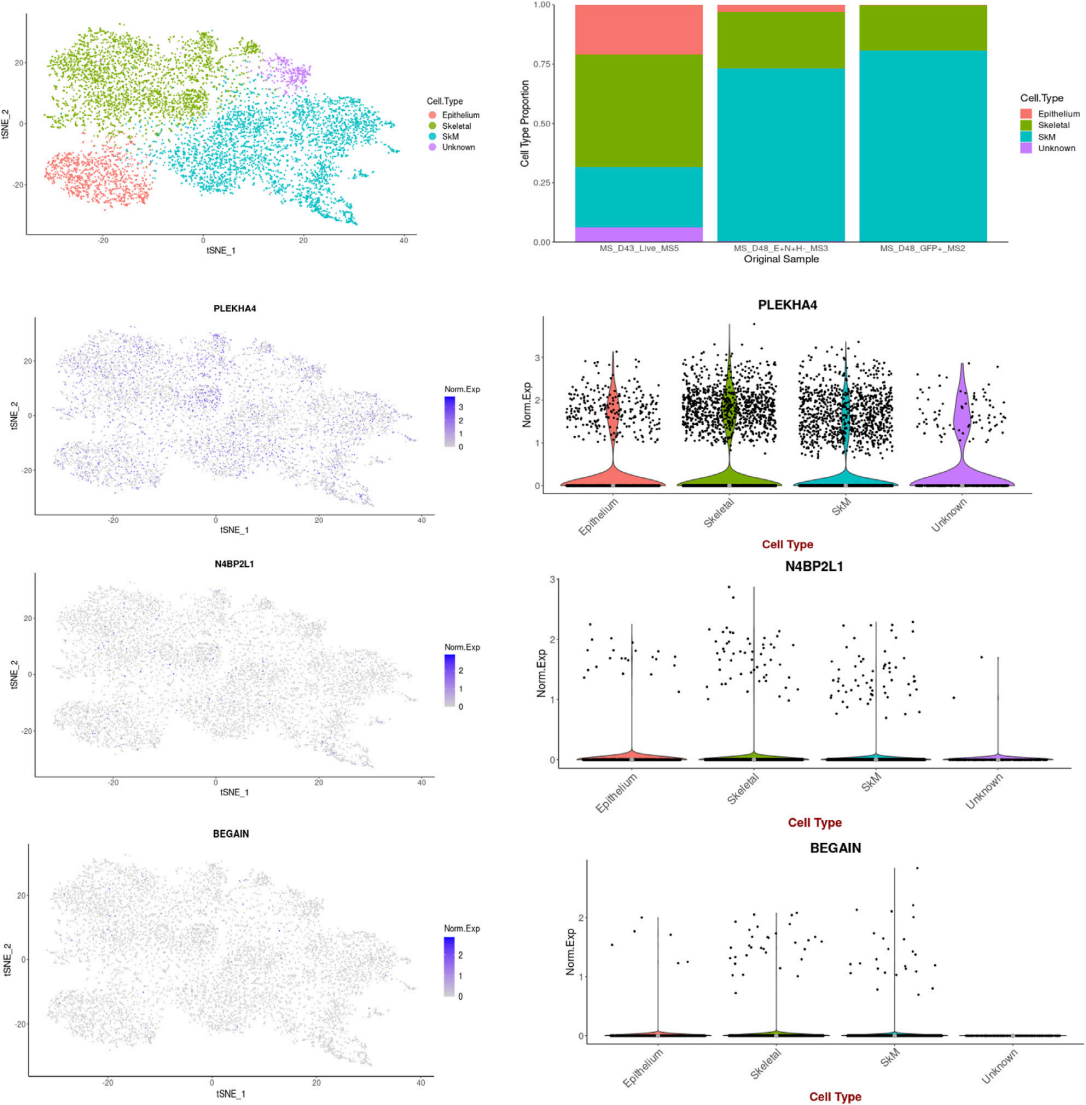
*PLEKHA4*, *N4BP2L1*, and *BEGAIN* expressions in muscle cells retrieved from the scRNA-seq data of the *in vitro* MS protocol.

Based on the data retrieved from the human muscle atlas, *PLEKHA4* is expressed in all muscle developmental stages, both in the hind limb muscle and myogenic subset, and its expression is higher than that of the other two genes. *N4BP2L1* also showed expression in the muscle developmental stages, except for the embryonic week 6–7 myogenic subset. We found that *BEGAIN* is mainly expressed in hind limb muscle in all of these developmental stages. The scRNA-seq data could separate different cell types in each muscle developmental stage, and the expression of these genes was different in each subset given in Table 4; Supplementary Figure S3. Moreover, using the scRNA-seq data of the *in vitro* MS protocol, the protocol used in our study, we found that these genes were also expressed in these muscle cells (Figure 5). The scRNA-seq data of the MS protocol distinguished different cell types, including epithelium, skeletal development, and skeletal muscle cells. These three genes showed expression in these subsets, with the highest expression for *PLEKHA4*, followed by *N4BP2L1* and *BEGAIN* (Figure 5).

**TABLE 4:** *PLEKHA4*, *N4BP2L1*, and *BEGAIN* expression in different cell types of *in vivo* muscle developmental stages.

Embryonic development	
Embryonic week 5–6 hind limb	*PLEKHA4*: SKM^1^, RBC^2^, Limb.Mesen^3^, Skin, ^4^WBC, ^5^EC
	*N4BP2L1*: Skin, Limb.Mesen, SKM, RBC, EC, WBC
	*BEGAIN*: EC, SKM, Limb.Mesen, RBC, Skin, WBC
Embryonic week 5–6 myogenic subset	*PLEKHA4*: ^6^MP
	*N4BP2L1*: MP
	*BEGAIN*: NO
Embryonic week 6–7 hind limb	*PLEKHA4*: Schwann, ^7^Chondro, SKM, Limb.Mesen, and ^8^PreChondro cells
	*N4BP2L1*: Schwann, Skin, Chondro, RBC, Limb.Mesen, PreChondro, and SKM cells
	*BEGAIN*: Chondro, PreChondro, and Limb.Mesen
Embryonic week 6–7 myogenic subset	*PLEKHA4*: ^9^MB-MC and MP
	*N4BP2L1*: NO
	*BEGAIN*: NO
Embryonic week 7–8 hind limb	*PLEKHA4*: Schwann, RBC, PreChondro, Chondro, SKM, Limb.Mesen^10^Dermal, Skin, and EC cells
	*N4BP2L1*: Skin, Chondro, SKM, EC, PreChondro, Limb.Mesen, RBC, Dermal and Schwann cells
	*BEGAIN*: SKM, Dermal, PreChondro
Embryonic week 7–8 myogenic subset	*PLEKHA4*: SKM.Other, MC, MB, MP
	*N4BP2L1*: MB, SKM.Other, MP, MC
	*BEGAIN*: NO
Fetal development	
Fetal week 09 hind limb	*PLEKHA4*: Schwann,^11^Teno,^12^SMC, Limb.Mesen, PreChondro, Chondro, RBC, EC, WBC, SKM, Dermal
	*N4BP2L1*: WBC, SKM, Chondro, PreChondro
	*BEGAIN*: RBC, EC, Teno, PreChondro, Lim.Mesen, Chondro
Fetal week 09 myogenic subset	*PLEKHA4*: MC, SKM.Other, MP
	*N4BP2L1*: MB, MC, SKM.Other
	*BEGAIN*: NO
Fetal week 12–14 hind limb muscle	*PLEKHA4*: Schwann, Teno, MSC, SMC, SKM, Dermal
	*N4BP2L1*: Teno, SKM, MSC
	*BEGAIN*: Teno, SKM, MSC
Fetal week 12–14 myogenic subset	*PLEKHA4*: MB-MC, SKM.Other, MP
	*N4BP2L1*: MB-MC, SKM. Other, MP
	*BEGAIN*: NO
Fetal week 17–18 hind limb muscle	*PLEKHA4*: Schwann, SMC, Teno, MSC, SKM
	*N4BP2L1*: SKM, MSC, SMC, Teno, Schwann
	*BEGAIN*: Schwann, MSC, SMC, SKM, Teno
Fetal week 17–18 myogenic subset	*PLEKHA4*: MB-MC, SKM.Other, MP
	*N4BP2L1*: MB-MC, SKM.Other, MP
	*BEGAIN*: SKM.Other, MP, MB-MC
Juvenile	
Juvenile hind limb muscle	*PLEKHA4*: ^13^SC
	*N4BP2L1*: SC
	*BEGAIN*: NO
Juvenile myogenic subset	*PLEKHA4*: ^14^FAP.1, SKM, FAP.2, SMC, EC, Teno, ^15^Hema
	*N4BP2L1*: Hema, EC, Teno, FAP.2, SKM, SMC, FAP.1
	*BEGAIN*: NO
Adult	
Adult hind limb muscle	*PLEKHA4*: SKM, Schwann, SMC, FAP, EC-Hema
	*N4BP2L1*: SKM, EC-Hema, FAP, SMC, Schwann
	*BEGAIN*: FAP, SMC, SKM, ^16^EC-Hema, Schwann
Adult myogenic subset	*PLEKHA4*: SC
	*N4BP2L1*: SC

1 SKM, skeletal muscle.

2 RBC, red blood cells.

3 Limb.Mesen, limb mesenchymal progenitors.

4 WBCs, white blood cells.

5 ECs, endothelial cells.

6 MP, myogenic progenitor.

7 Chondro, chondrogenic cells.

8 PreChondro, prechondrogenic cells.

9 MBs-MCs, myoblasts–myocytes.

10 Dermal, dermal fibroblasts and progenitors.

11 Teno, tenogenic cells.

12 SMCs, smooth muscle cells.

13 SCs, postnatal satellite cells.

14 FAPs, fibro-adipogenic progenitors.

15 Hema, hematopoietic lineages.

16 EC-Hema, endothelial-hematopoietic.

### 3.8 Higher expression of *PLEKHA4* and *BEGAIN* in MEP cells in comparison with LEP cells

**FIGURE 6:**
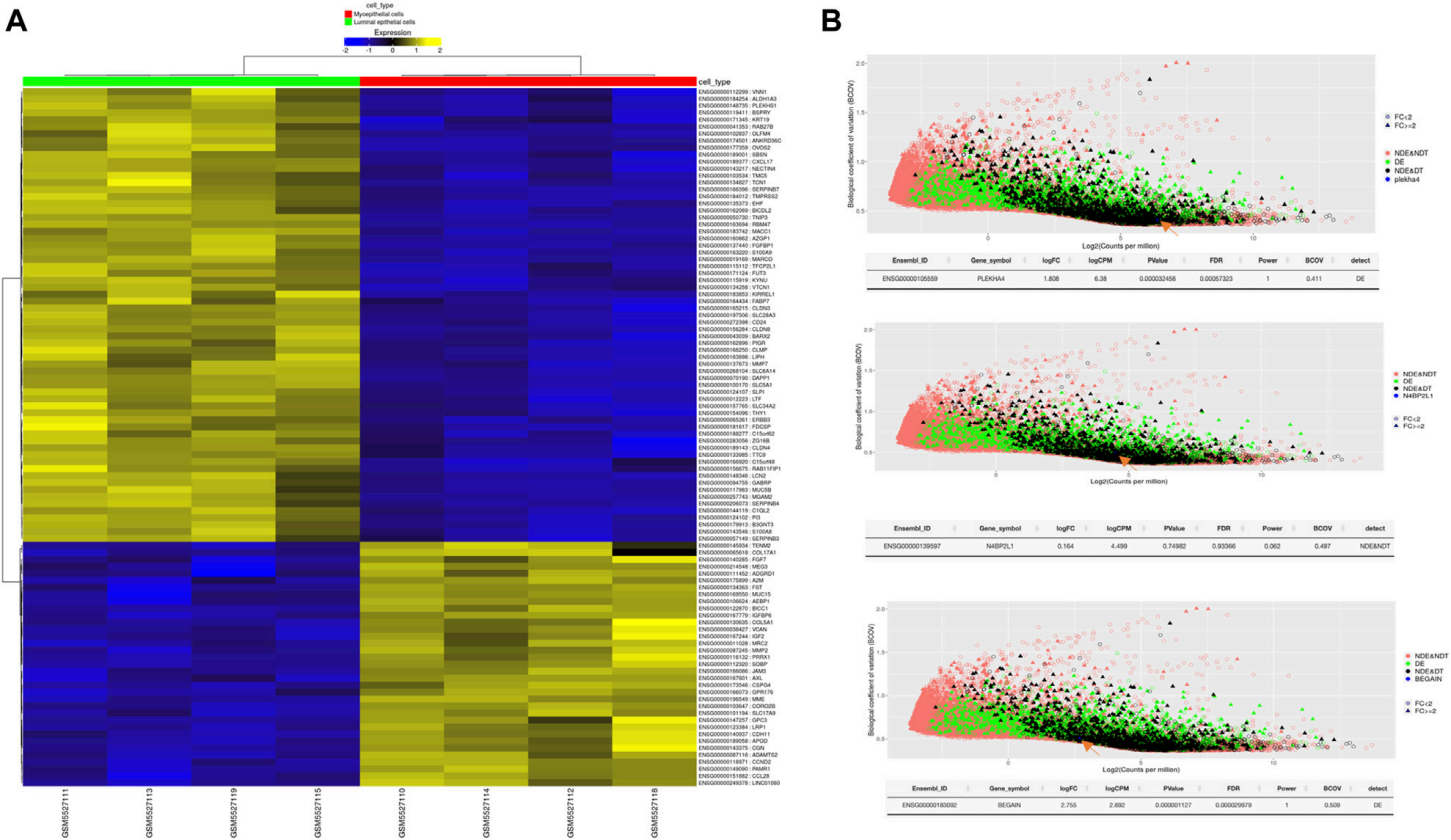
**(A)** Heatmap of differentially expressed genes between MEP and LEP cells isolated from primary breast organoids. **(B)** Power analysis of our proposed genes involved in muscle cells and breast cancer. The statistical power for each of the genes to be detected as differentially expressed between the selected groups is estimated. The plot shows the effect of BCOV and mean coverage on the detectability of the genes. logCPM, log counts per million; BCOV, biological coefficient of variation.

Our results revealed statistically significant higher expression of *PLEKHA4* (logFC: 1.81 and FDR: 0.0006) and *BEGAIN* (logFC: 2.76 and FDR: 0.00003) in MEP compared to LEP cells. However, for *N4BP2L1*, the expression was not different (logFC: 0.164 and FDR: 0.93) between these two cell types of organoids, representing their possible function in both of these cells (Figure 6; Table 5).

**TABLE 5:** Differential expression of some important tumor suppressor genes and our proposed genes between MEP and LEP cells isolated from primary breast organoid.

Ensembl_ID	Gene_symbol	logFC	logCPM	*p*-value	FDR
ENSG00000105559	*PLEKHA4*	1.808	6.38	0.000032	0.00057
ENSG000000183092	*BEGAIN*	2.755	2.692	0.0000011	0.00003
ENSG000000139597	*N4BP2L1*	0.164	4.499	0.749	0.933
ENSG00000073282	*TP63*	4.886	5.127	4.313e-12	4.041e-10
ENSG00000139687	*RB1*	1.164	5.264	0.002238	0.02006
ENSG00000206075	*SERPINB5*	2.878	6.557	0.00007625	0.001204

We also identified higher expression of some important tumor suppressor genes as expected, such as *TP63* (logFC:4.886 and FDR:4.041e-10), *RB1* (logFC:1.164 and FDR:0.02), and *SERPINB5* (logFC:2.88 and FDR:0.0012) in MEP cells compared with LEP cells (Table 5). Therefore, the higher expression of these genes in normal MEP compared to LEP cells should be considered for future functional studies to find their exact roles in these cells and breast tumors.

### 3.9 *N4BP2L1*, *PLEKHA4*, and *BEGAIN* upregulation in *in vitro* muscle cells differentiated from hESCs

**FIGURE 7:**
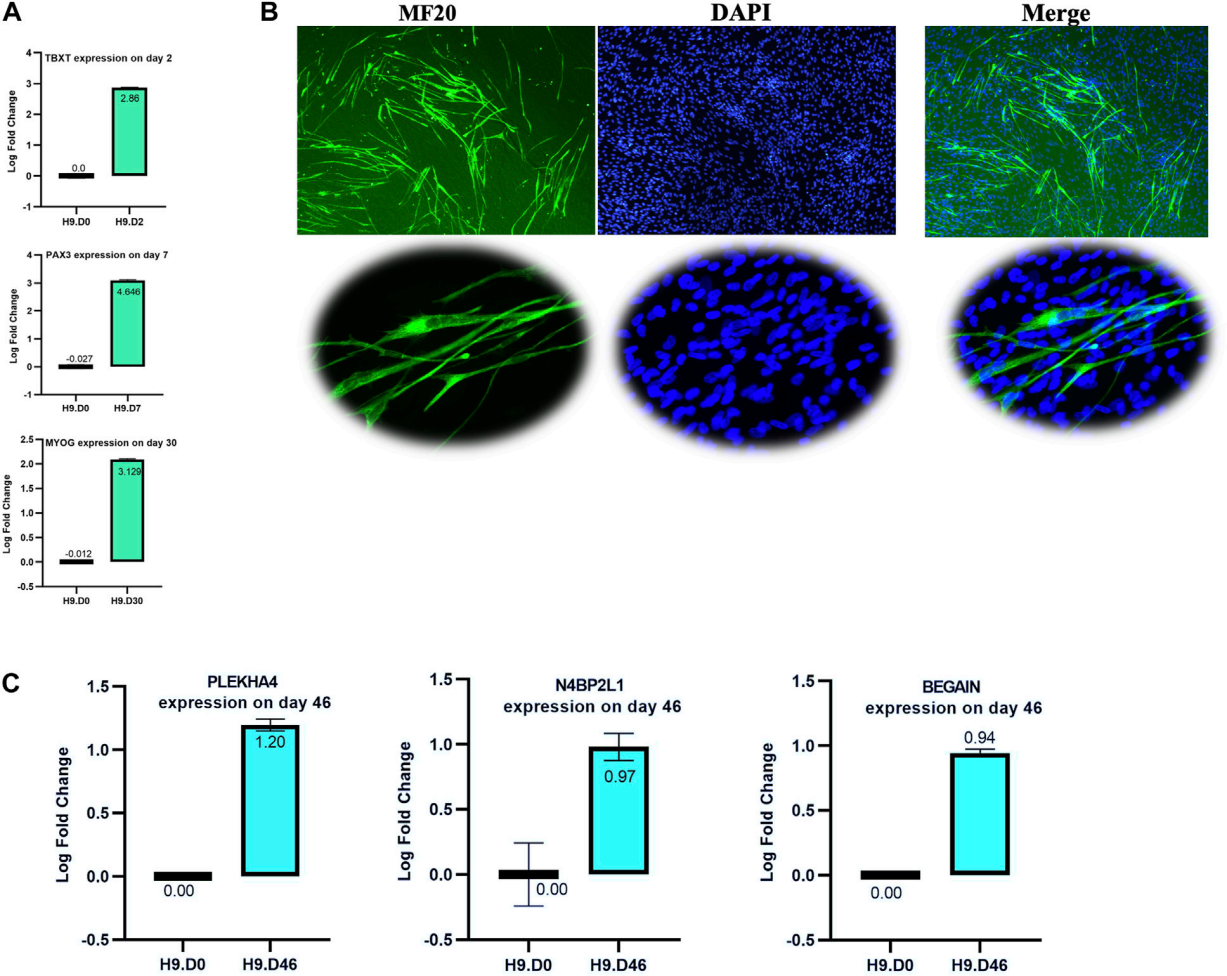
Validation of the muscle differentiation process. **(A)** RT-qPCR of selected stage marker genes shows a higher relative expression: *TBXT* on day 2, *PAX3* on day 7, and *MYOG* on day 30. **(B)** Immunofluorescence staining of the myosin heavy chain using the MF20 antibody on day 46 of the muscle cell process. The lower image shows some multinucleated cells in our muscle cell process. **(C)** RT-qPCR analysis of *N4BP2L1*, *PLEKHA4*, and *BEGAIN* expression in muscle cells differentiated from hESCs.

To evaluate the involvement of *N4BP2L1, PLEKHA4,* and *BEGAIN* genes in muscle cell development, we investigated their expression levels by performing RT-qPCR on muscle cells differentiated from hESCs (D0: stem stage and D46: terminally differentiated stage). The muscle differentiation process was confirmed by controlling the expression of specific stage marker genes, including *TBXT* (paraxial mesoderm stage marker) on day 2, *PAX3* (the somite stage marker) on day 7, *MYOG* (a marker of skeletal muscle progenitors’ stage) on day 30 using qRT-PCR (Figure 7A), and myosin heavy chain (detected by MF20 antibody) by IF staining on day 46 (Figure 7B), confirming the expected behavior of stage-specific markers.

In our muscle differentiation data, we found higher expression of *N4BP2L1*, *PLEKHA4*, and *BEGAIN* using RT-qPCR in muscle cells compared to H9 hESCs, as shown in Figure 7C. Among these genes, only *PLEKHA4* functions as a transcription factor that promotes Wnt signaling through the inhibition of cytoplasmic protein disheveled (DVL) degradation (Shami Shah et al., 2019). Our data represent the role of *N4BP2L1, PLEKHA4*, and *BEGAIN* in muscle cells and breast tissue given their higher expression level in these cells compared to hESCs and their lower expression in breast cancer, representing their function as tumor-suppressive genes.

## 4 Discussion

In the current study, we found that *PLEKHA4*, *BEGAIN*, and *N4BP2L1* correlated with patient prognosis as possible tumor suppressors with a role in muscle cells. Our result revealed a *PLEKHA4* expression correlation with the age of menopause in our cohort of patients with breast cancer (Supplementary Table S1; panel A). Furthermore, in this cohort, we showed *N4BP2L1* and *BEGAIN* as potential biomarkers of HER2-positive breast cancer (Supplementary Table S1; panels B and C). By searching Enrichr, we identified the involvement of these genes in biological processes critical for muscle cell differentiation (Figure 2).

Using the gene expression analysis of these genes in different patient statuses of TCGA-BRCA, our data could propose their function in tumorigenesis. The TCGA data analysis revealed significant downregulation of *N4BP2L1*, *PLEKHA4*, and *BEGAIN* genes not only in breast cancer but also in other main cancers (Supplementary Table S4). Moreover, all three genes had a significantly decreased expression in all TCGA-BRCA tumor stages compared to their matched normal tissues, representing their probable role in tumor metastasis (Supplementary Figure S1; panel 1). Furthermore, these genes showed significant downregulation in all subclasses of TCGA-BRCA compared to normal tissues (Supplementary Figure S1; panel 2), with the highest significant value for the HER2-positive subclass. Additionally, they had downregulation in various TNBC subclasses compared to normal tissues. Since *N4BP2L1* and *PLEKHA4* genes also had significantly decreased expression in TNBC-MSL and TNBC-M originated from mesoderm lineage, essential for the muscle differentiation process (Supplementary Figure S1; panel 3), it may indicate that their involvement is not only in breast cancer but also in muscle development. In addition, based on the histologic subtypes of TCGA-BRCA, we found significantly lower expression of these genes in their subtypes (Supplementary Figure S1; panel 4). *N4BP2L1* may also play an important role in metaplastic breast cancer due to its significantly lower expression in this subtype compared to normal. Moreover, the analysis of these genes in nodal metastasis statuses of TCGA-BRCA revealed their significant downregulation in all statuses compared to the normal one, representing their probable function in breast cancer metastasis (Supplementary Figure S1; panel 5). Since NRLN metastases are considered in the prognosis assessment and clinical control of cancers, we proposed *PLEKHA4* and *BEGAIN* as a prognostic biomarker set.

To extend the possible functionality of *N4BP2L1* as a protein biomarker in breast cancer, we could find its weak expression in most of the TCGA-BRCA tumor samples (66%) (Supplementary Figure S2; panel A), its significantly lower expression in primary breast tumors of the CPTAC dataset compared to their matched normal tissues (Supplementary Figure S2; panel D), and its downregulation in different breast tumor subclasses, histological subtypes, pathways, and patients’ statues (Supplementary Figure S2; panel E).

The bulk RNA-seq data and protein expression data of these uncharacterized tumor suppressor genes in normal breast tissues collected in the GTEx databases revealed a high level of expression for *PLEKHA4* and a considerable level for *N4BP2L1* and *BEGAIN.* The single-cell RNA-seq data available from the Human Protein Atlas uncovered the different expression patterns of these genes in different cell types of breast tissue. Notably, these genes were expressed in mesenchymal cells, breast myoepithelial cells, and smooth muscle cells, indicating their possible involvement in muscle cells (Figure 4), as found by their upregulation in muscle cells differentiated from hESCs and also scRNA-seq data of *in vivo* muscle developmental stages (Figure 7C and 5; Supplementary Figure S3).

As mentioned in the result section, at the single-cell RNA level, we found that *PLEKHA4* exhibited high expression not only in cells related to the early muscle stages and their precursors but also showed the highest expression in smooth muscle cells, C-14 (nTPM: 36.2) (Figure 4). Moreover, as expected in our muscle differentiation process, it had higher expression in terminally differentiated muscle cells (D46) (Figure 7C), representing its probable role in these cells.

In addition to the above-mentioned data, based on the data extracted from the CTD dataset, all these genes showed an association with both breast cancer and muscular disorders (Supplementary Table S5), which strengthens their possible function in these disorders.

*PLEKHA4* is encoded for a plasma membrane-localized signaling protein that functions as a positive regulator of DVL protein levels through the inhibition of its polyubiquitination. This gene is a positive modulator of Wnt signaling pathways (canonical, β-catenin-dependent and non-canonical, β-catenin-independent ones) by the recruitment of the CUL3-KLHL12 E3 ligase complex to the plasma membrane, which reduces DVL protein ubiquitination, helps in increasing the DVL levels, and subsequently enhances and strengthens Wnt signaling pathways (Angers et al., 2006; Shami Shah et al., 2019). Its expression levels in our muscle differentiation stages can indicate its role in this pathway, and its downregulation in our breast cancer samples and also in TCGA-BRCA can show its role in this cancer.

*N4BP2L1* is a target gene of *FoxO1* and has an important function in the insulin signaling pathway due to its role in GLUT4-mediated glucose uptake, supporting its key role in adipocyte differentiation (Watanabe et al., 2019; Watanabe et al., 2021). Since the insulin signaling pathway is involved with the skeletal muscle (Ijuin et al., 2015) and breast cancer (Yee et al., 2020), *N4BP2L1* can be involved in this process and breast cancer, as found in our study, with its upregulation during muscle differentiation and its downregulation in our breast cancer samples, as well as TCGA-BRCA at both the RNA and protein levels.

One study in sheep showed that *BEGAIN* is ubiquitously expressed in different tissues, such as skeletal muscle, at all times of development. Moreover, in the ovine, it was found as a paternally expressed gene in the ovine *DLK1-GTL2-*imprinted domain, expressed in the brain, kidney, liver, and skeletal muscle (Smit et al., 2005). *BEGAIN* was reported to play an important role in the transmission of pathological pain through the activation of the spinal cord NMDA receptor (NMDAR) in its inner lamina II (Katano et al., 2016). Therefore, based on our findings, its higher expression during muscle cell differentiation stages compared to hESCs can show its involvement in muscle cells, and its downregulation in our breast samples and TCGA-BRCA can represent its role in this cancer as a tumor suppressor gene.

In conclusion, our results proposed the important functions of these genes in muscle cells and breast tissue due to their notable upregulation in these cells compared to hESCs, their expression in muscle cells during *in vivo* muscle development, and their significant downregulation in different statuses of patients with breast cancer, indicating their function as possible tumor-suppressive genes.

## Data Availability

In the current study, some publicly available datasets were analyzed for the involvement of genes in cancers including TCGA, Genecards, Human Protein Atlas, and Enrichr. The raw data can be found here: https://tcga‐data.nci.nih.gov/tcga/, https://www.genecards.org, https://www.proteinatlas.org, and https://maayanlab.cloud/Enrichr/. Concerning the GDCRNATools, we used the R codes from the following link: https://github.com/Jialab-UCR/GDCRNATools. Regarding data of in vivo human skeletal muscle development and primary breast organoids, we used the publicly available dataset aprilpylelab (https://aprilpylelab.com/datasets/) and GREIN tools (http://www.ilincs.org/apps/grein/?gse=GSE182338), respectively.
