# Supplementary material for: Involvement of N4BP2L1, PLEKHA4, and BEGAIN genes in breast cancer and muscle cell development
Source: Front Cell Dev Biol. 2024 May 24;12:1295403. doi: 10.3389/fcell.2024.1295403 (PMC11163233; doi:10.3389/fcell.2024.1295403)
Supplement: Supplementary file 1 [file DataSheet1.zip › Supplementary files/Supplementary Table S1.pdf]

Supplementary Table S1. All clinical data of our patients and their correlation with *N4BP2L1, PLEKHA4*, and *BEGAIN* expression.

Panel A. All clinical data of our patients and correlation between *PLEKHA4* and patient status.

	Mean	Valid N	Standard Deviation	Median	P-value
ΔΔN_PLEKHA4	.040	50	1.123	-.043	0.697
ΔΔT_PLEKHA4	1.587	50	1.413	1.432	
LogFC_N_PLEKHA4	-.0122	50	.3375	.0100	<0.001
LogFC_T_PLEKHA4	-.4778	50	.4255	.-.4314	

		LogFC.T.PLEKH				
		Valid N	Mean	Standard Deviation	Median	P-value
Age cutpoint	<50	21	-.5829	.4323	-.4597	0.208
	≥50	29	-.4017	.4113	-.3904	
Tumor size cutpoint	<2.5	30	-.4893	.4851	-.3997	0.921
	≥2.5	20	-.4605	.3275	-.4345	
Estrogen receptor	negative	5	-.3942	.2592	-.3391	0.528
	positive	45	-.4871	.4412	-.4449	
Progesterone receptor	negative	7	-.5121	.2963	-.4597	0.856
	positive	43	-.4722	.4455	-.4179	
HER-2 receptor	negative	34	-.4484	.4706	-.3831	0.499
	positive	16	-.5401	.3132	-.4480	
Nuclear grade	1	8	-.3258	.3487	-.2248	0.066
	2	34	-.4550	.4449	-.4314	
	3	8	-.7267	.3363	-.8126	
Histologic grade	1	12	-.3328	.4277	-.3619	0.258
	2	23	-.5857	.4149	-.5346	
	3	15	-.4284	.4240	-.3757	
Lymph nodes metastasis	NO	34	-.4648	.4743	-.4314	0.992
	YES	16	-.5053	.3089	-.4552	

Histologic type of invasive carcinoma	ILC	1	-.1343	.	-.1343	0.177
	IDC	49	-.4848	.4270	-.4449	
Marital status	married	48	-.4803	.4342	-.4314	1.000
	single	2	-.4177	.0594	-.4177	
Cutpoint of BMI	≤25	15	-.4224	.4970	-.4179	0.971
	25-29	23	-.4795	.4197	-.4510	
	≥30	12	-.5436	.3622	-.5274	
Age of menarch	<14	29	-.4916	.4646	-.4510	0.673
	≥14	21	-.4587	.3752	-.4179	
Is menstruation regular?	NO	5	-.5750	.4574	-.4510	0.808
	YES	45	-.4670	.4260	-.4179	
Age of menpause	<50	27	-.6173	.3788	-.6308	0.009
	≥50	23	-.3140	.4264	-.2907	
Menopausal status	pre	11	-.6582	.2658	-.7328	0.052
	post	39	-.4269	.4504	-.3546	
History of uterine surgery	NO	47	-.4730	.4366	-.4179	0.728
	YES	3	-.5531	.2098	-.6073	
Number of pregnancy	≤3	34	-.4410	.4541	-.4480	0.560
	>3	16	-.5560	.3582	-.4042	
Age at FFTP	<25	33	-.4968	.4388	-.5317	0.908
	≥25	12	-.5472	.4145	-.3755	
Breastfeeding duration	0	4	-.1905	.3291	-.2959	0.270
	<24	26	-.4412	.4621	-.4314	
	≥24	20	-.5829	.3716	-.5331	
Number of abortion	0	34	-.4325	.4171	-.3681	0.256
	1	13	-.5284	.4640	-.4179	
	>1	3	-.7719	.3060	-.8041	
Abortion history	NO	34	-.4325	.4171	-.3681	0.205
	YES	16	-.5741	.4408	-.5907	
Family history of cancer	NO	23	-.4280	.3638	-.3546	0.345

	YES	27	-.5202	.4746	-.5317	
Diseases history	NO	26	-.5098	.5016	-.4480	0.360
	LOCUS	1	-.5317	.	-.5317	
	Thyroid problem	2	-.2927	.1771	-.2927	
	blood pressure	11	-.5761	.2234	-.6073	
	blood fat	6	-.2477	.4946	-.2160	
	heart problem	1	-.8069	.	-.8069	
	Diabetes	3	-.2960	.2245	-.2277	
Chest radiograph history	NO	21	-.4967	.5230	-.4597	0.398
	YES	29	-.4641	.3479	-.3757	
Hormone therapy history	NO	48	-.4793	.4331	-.4314	0.843
	YES	2	-.4415	.2345	-.4415	
Duration of OCP consumption	0	28	-.4446	.3935	-.4177	0.928
	≤5	16	-.4957	.4942	-.3863	
	>5	6	-.5848	.4300	-.5770	
OCP consumption	NO	28	-.4446	.3935	-.4177	0.777
	YES	22	-.5200	.4692	-.4823	
Vitamin D consumption	NO	27	-.4860	.4953	-.4449	0.969
	YES	23	-.4681	.3364	-.4179	
Regular sleep	NO	19	-.4226	.4725	-.3904	0.454
	YES	31	-.5116	.3984	-.4449	
Exercising	NO	33	-.4900	.4660	-.4597	0.616
	YES	17	-.4540	.3454	-.4179	
Deodorant use	NO	33	-.4560	.4780	-.3757	0.743
	YES	17	-.5202	.3078	-.4597	
Hair dye use	NO	10	-.4942	.3830	-.2835	0.698
	YES	40	-.4737	.4400	-.4480	
Cosmetics use	NO	29	-.4935	.4816	-.3546	0.875
	YES	21	-.4562	.3436	-.4449	

		LogFC_T_PLEKHA4_cut				
		Low		High		
		Count	Row N %	Count	Row N %	P-value
Age_cutpoint	<50	12	57.1%	9	42.9%	0.390
	≥50	13	44.8%	16	55.2%	
Tumor size_cutpoint	<2.5	15	50.0%	15	50.0%	1.000
	≥2.5	10	50.0%	10	50.0%	
Estrogen receptor	negative	2	40.0%	3	60.0%	0.637
	positive	23	51.1%	22	48.9%	
Progesterone receptor	negative	4	57.1%	3	42.9%	0.684
	positive	21	48.8%	22	51.2%	
HER-2 receptor	negative	16	47.1%	18	52.9%	0.544
	positive	9	56.3%	7	43.8%	
Nuclear grade	1	2	25.0%	6	75.0%	0.135
	2	17	50.0%	17	50.0%	
	3	6	75.0%	2	25.0%	
Histologic grade	1	5	41.7%	7	58.3%	0.364
	2	14	60.9%	9	39.1%	
	3	6	40.0%	9	60.0%	
Lymph nodes metastasis	NO	17	50.0%	17	50.0%	1.000
	YES	8	50.0%	8	50.0%	
Histologic type of invasive carcinoma	ILC	0	0.0%	1	100.0%	0.312
	IDC	25	51.0%	24	49.0%	
Marital status	married	24	50.0%	24	50.0%	1.000
	single	1	50.0%	1	50.0%	
Cutpoint_of_BMI	≤25	7	46.7%	8	53.3%	0.946
	25-29	12	52.2%	11	47.8%	

	≥30	6	50.0%	6	50.0%	
Age of menarche	<14	15	51.7%	14	48.3%	0.774
	≥14	10	47.6%	11	52.4%	
Is menstruation regular?	NO	3	60.0%	2	40.0%	0.637
	YES	22	48.9%	23	51.1%	
Age of menopause	<50	18	66.7%	9	33.3%	0.011
	≥50	7	30.4%	16	69.6%	
Menopausal status	pre	8	72.7%	3	27.3%	0.088
	post	17	43.6%	22	56.4%	
History of uterine surgery	NO	23	48.9%	24	51.1%	0.552
	YES	2	66.7%	1	33.3%	
Number of pregnancy	≤3	18	52.9%	16	47.1%	0.544
	>3	7	43.8%	9	56.3%	
Age at FFTP	<25	19	57.6%	14	42.4%	0.344
	≥25	5	41.7%	7	58.3%	
Breastfeeding duration	0	1	25.0%	3	75.0%	0.549
	<24	13	50.0%	13	50.0%	
	≥24	11	55.0%	9	45.0%	
Number of abortion	0	16	47.1%	18	52.9%	0.202
	1	6	46.2%	7	53.8%	
	>1	3	100.0%	0	0.0%	
Abortion history	NO	16	47.1%	18	52.9%	0.544
	YES	9	56.3%	7	43.8%	
Family history of cancer	NO	9	39.1%	14	60.9%	0.156
	YES	16	59.3%	11	40.7%	
Diseases history	NO	14	53.8%	12	46.2%	0.240
	LOCUS	1	100.0%	0	0.0%	
	Thyroid problem	0	0.0%	2	100.0%	
	blood pressure	7	63.6%	4	36.4%	
	blood fat	1	16.7%	5	83.3%	
	heart problem	1	100.0%	0	0.0%	
	Diabetes	1	33.3%	2	66.7%	

Chest radiograph history	NO	12	57.1%	9	42.9%	0.390
	YES	13	44.8%	16	55.2%	
Hormone therapy history	NO	24	50.0%	24	50.0%	1.000
	YES	1	50.0%	1	50.0%	
Duration of OCP consumption	0	14	50.0%	14	50.0%	0.632
	≤5	7	43.8%	9	56.3%	
	>5	4	66.7%	2	33.3%	
OCP consumption	NO	14	50.0%	14	50.0%	1.000
	YES	11	50.0%	11	50.0%	
Vitamin D consumption	NO	14	51.9%	13	48.1%	0.777
	YES	11	47.8%	12	52.2%	
Regular sleep	NO	9	47.4%	10	52.6%	0.771
	YES	16	51.6%	15	48.4%	
Exercising	NO	17	51.5%	16	48.5%	0.765
	YES	8	47.1%	9	52.9%	
Deodorant use	NO	16	48.5%	17	51.5%	0.765
	YES	9	52.9%	8	47.1%	
Hair dye use	NO	4	40.0%	6	60.0%	0.480
	YES	21	52.5%	19	47.5%	
Cosmetics use	NO	14	48.3%	15	51.7%	0.774
	YES	11	52.4%	10	47.6%	

	LogFC_T_PLEKHA4_cut						
	Low			High			
	Valid N	Standard Deviation	Mean	Valid N	Standard Deviation	Mean	P-value
Age (years)	25	9	51	25	10	53	0.576
BMI	25	4	28	25	4	27	0.777

Correlations

			Age (years) <50 or ≥50	BMI	LogFC.T.PLEKHA4
Spearman's rho	Age (years) <50 or ≥50	Correlation Coefficient	1.000	-.052	.148
		Sig. (2-tailed)	.	.721	.304
		N	50	50	50
	BMI	Correlation Coefficient	-.052	1.000	-.057
		Sig. (2-tailed)	.721	.	.694
		N	50	50	50
	LogFC.T.PLEKHA4	Correlation Coefficient	.148	-.057	1.000
		Sig. (2-tailed)	.304	.694	.
		N	50	50	50

Panel B. All clinical data of our patients and correlation between *N4BP2L1* and patient status.

	Valid N	Mean	Standard Deviation	Median	P-value
ΔΔN_N4BP2L1	50	-.154	2.123	.010	0.565
ΔΔT_N4BP2L1	50	3.127	1.258	3.085	
FC_N_N4BP2L1	50	.0466	.6391	-.0031	<0.001
FC_T_N4BP2L1	50	-.9414	.3785	-.9303	

		LogFC.T.N4BP2L1				
		Valid N	Mean	Standard Deviation	Median	P-value
Age cutpoint	<50	21	-.9731	.3584	-.9469	0.694
	≥50	29	-.9184	.3970	-.9136	
Tumor size_cutpoint	<2.5	30	-.9546	.3927	-1.0333	0.628
	≥2.5	20	-.9215	.3652	-.8962	
Estrogen receptor	negative	5	-.8970	.2190	-.8827	0.571
	positive	45	-.9463	.3936	-.9469	

Progesterone receptor	negative	7	-.9326	.2002	-.8928	0.748
	positive	43	-.9428	.4017	-.9469	
HER-2 receptor	negative	34	-.8589	.3847	-.8434	0.043
	positive	16	-1.1167	.3069	-1.1279	
Nuclear grade	1	8	-.9968	.2777	-1.1279	0.066
	2	34	-.9289	.4235	-.9303	
	3	8	-.9388	.2754	-.8945	
Histologic grade	1	12	-.9442	.4940	-1.0670	0.258
	2	23	-.9867	.3788	-1.1079	
	3	15	-.8695	.2744	-.8041	
Lymph nodes metastasis	NO	34	-.9378	.4362	-.9469	0.787
	YES	16	-.9488	.2219	-.9049	
Histologic type of invasive carcinoma	ILC	1	-.7959	.	-.7959	0.510
	IDC	49	-.9443	.3818	-.9469	
Marital status	married	48	-.9454	.3858	-.9469	0.520
	single	2	-.8434	.0556	-.8434	
Cutpoint of BMI	≤25	15	-.8566	.4958	-1.1871	0.971
	25-29	23	-.9978	.2884	-.9469	
	≥30	12	-.9391	.3774	-.9014	
Age of menarch	<14	29	-.9526	.4419	-.9469	0.426
	≥14	21	-.9258	.2779	-.8729	
Is menstruation regular?	NO	5	-.9438	.2161	-.9469	0.948
	YES	45	-.9411	.3941	-.9136	
Age of menpause	<50	27	-1.0188	.3215	-.9586	0.080
	≥50	23	-.8505	.4254	-.8827	
Menopausal status	pre	11	-1.0778	.3014	-1.1308	0.232
	post	39	-.9029	.3923	-.9066	
History of uterine surgery	NO	47	-.9296	.3846	-.9136	0.347
	YES	3	-1.1259	.2302	-1.1249	
Number of pregnancy	≤3	34	-.9500	.4114	-.9528	0.819
	>3	16	-.9230	.3085	-.9101	
Age at FFTP	<25	33	-.9570	.3757	-.9136	0.827
	≥25	12	-.9717	.3678	-1.0333	
Breastfeeding duration	0	4	-.6399	.4188	-.7960	0.270
	<24	26	-.9484	.4094	-.9101	
	≥24	20	-.9924	.3150	-1.1221	
Number of abortion	0	34	-.9647	.4234	-1.1135	0.256

	1	13	-.8546	.2725	-.8928	
	>1	3	-1.0524	.1728	-.9586	
Abortion history	NO	34	-.9647	.4234	-1.1135	0.432
	YES	16	-.8917	.2641	-.9049	
Family history of cancer	NO	23	-.9464	.3796	-.9136	0.830
	YES	27	-.9370	.3847	-.9469	
Diseases history	NO	26	-.8930	.4409	-.8895	0.360
	LOCUS	1	-1.1938	.	-1.1938	
	Thyroid problem	2	-.8624	.5731	-.8624	
	blood pressure	11	-1.0432	.2189	-1.1192	
	blood fat	6	-.7989	.2953	-.8443	
	heart problem	1	-1.3188	.	-1.3188	
	Diabetes	3	-1.1144	.3941	-1.2218	
Chest radiograph history	NO	21	-.9662	.4296	-.9586	0.426
	YES	29	-.9234	.3436	-.9066	
Hormone therapy history	NO	48	-.9389	.3792	-.9303	0.804
	YES	2	-.9993	.5052	-.9993	
Duration of OCP consumption	0	28	-.9760	.3299	-.9528	0.928
	≤5	16	-.7814	.3945	-.8460	
	>5	6	-1.2064	.4201	-1.2588	
OCP consumption	NO	28	-.9760	.3299	-.9528	0.494
	YES	22	-.8973	.4367	-.9101	
Vitamin D consumption	NO	27	-.9217	.4238	-.9136	0.907
	YES	23	-.9645	.3252	-1.1079	
Regular sleep	NO	19	-.9305	.4153	-.9469	0.522
	YES	31	-.9480	.3611	-.8928	
Exercising	NO	33	-.9147	.3896	-.9136	0.984
	YES	17	-.9931	.3618	-.9469	
Deodorant use	NO	33	-.9118	.4181	-.9066	0.486
	YES	17	-.9988	.2894	-1.1079	
Hair dye use	NO	10	-.8777	.2697	-.9049	0.416
	YES	40	-.9573	.4024	-.9528	
Cosmetics use	NO	29	-.8982	.4196	-.9136	0.637
	YES	21	-1.0009	.3131	-1.1079	

		LogFC_T_N4BP2L1_cut				
		Low		High		
		Count	Row N %	Count	Row N %	P-value
Age_cutpoint	<50	11	52.4%	10	47.6%	0.774
	≥50	14	48.3%	15	51.7%	
Tumor size_cutpoint	<2.5	17	56.7%	13	43.3%	0.248
	≥2.5	8	40.0%	12	60.0%	
Estrogen receptor	negative	1	20.0%	4	80.0%	0.157
	positive	24	53.3%	21	46.7%	
Progesterone receptor	negative	2	28.6%	5	71.4%	0.221
	positive	23	53.5%	20	46.5%	
HER-2 receptor	negative	13	38.2%	21	61.8%	0.015
	positive	12	75.0%	4	25.0%	
Nuclear grade	1	5	62.5%	3	37.5%	0.607
	2	17	50.0%	17	50.0%	
	3	3	37.5%	5	62.5%	
Histologic grade	1	7	58.3%	5	41.7%	0.303
	2	13	56.5%	10	43.5%	
	3	5	33.3%	10	66.7%	
Lymph nodes metastasis	NO	18	52.9%	16	47.1%	0.544
	YES	7	43.8%	9	56.3%	
Histologic type of invasive carcinoma	ILC	0	0.0%	1	100.0%	0.312
	IDC	25	51.0%	24	49.0%	
Marital status	married	25	52.1%	23	47.9%	0.149
	single	0	0.0%	2	100.0%	
Cutpoint_of_BMI	≤25	8	53.3%	7	46.7%	0.801
	25-29	12	52.2%	11	47.8%	
	≥30	5	41.7%	7	58.3%	
Age of menarche	<14	16	55.2%	13	44.8%	0.390
	≥14	9	42.9%	12	57.1%	

Is menstruation regular?	NO	3	60.0%	2	40.0%	0.637
	YES	22	48.9%	23	51.1%	
Age of menopause	<50	15	55.6%	12	44.4%	0.395
	≥50	10	43.5%	13	56.5%	
Menopausal status	pre	6	54.5%	5	45.5%	0.733
	post	19	48.7%	20	51.3%	
History of uterine surgery	NO	23	48.9%	24	51.1%	0.552
	YES	2	66.7%	1	33.3%	
Number of pregnancy	≤3	18	52.9%	16	47.1%	0.544
	>3	7	43.8%	9	56.3%	
Age at FFTP	<25	16	48.5%	17	51.5%	0.559
	≥25	7	58.3%	5	41.7%	
Breastfeeding duration	0	1	25.0%	3	75.0%	0.376
	<24	12	46.2%	14	53.8%	
	≥24	12	60.0%	8	40.0%	
Number of abortion	0	18	52.9%	16	47.1%	0.080
	1	4	30.8%	9	69.2%	
	>1	3	100.0%	0	0.0%	
Abortion history	NO	18	52.9%	16	47.1%	0.544
	YES	7	43.8%	9	56.3%	
Family history of cancer	NO	11	47.8%	12	52.2%	0.777
	YES	14	51.9%	13	48.1%	
Diseases history	NO	12	46.2%	14	53.8%	0.778
	LOCUS	1	100.0%	0	0.0%	
	Thyroid problem	1	50.0%	1	50.0%	
	blood pressure	6	54.5%	5	45.5%	
	blood fat	2	33.3%	4	66.7%	
	heart problem	1	100.0%	0	0.0%	
	Diabetes	2	66.7%	1	33.3%	
Chest radiograph history	NO	11	52.4%	10	47.6%	0.774
	YES	14	48.3%	15	51.7%	
	NO	24	50.0%	24	50.0%	1.000

Hormone therapy history	YES	1	50.0%	1	50.0%	
Duration of OCP consumption	0	15	53.6%	13	46.4%	0.080
	≤5	5	31.3%	11	68.8%	
	>5	5	83.3%	1	16.7%	
OCP consumption	NO	15	53.6%	13	46.4%	0.569
	YES	10	45.5%	12	54.5%	
Vitamin D consumption	NO	13	48.1%	14	51.9%	0.777
	YES	12	52.2%	11	47.8%	
Regular sleep	NO	10	52.6%	9	47.4%	0.771
	YES	15	48.4%	16	51.6%	
Exercising	NO	16	48.5%	17	51.5%	0.765
	YES	9	52.9%	8	47.1%	
Deodorant use	NO	15	45.5%	18	54.5%	0.370
	YES	10	58.8%	7	41.2%	
Hair dye use	NO	4	40.0%	6	60.0%	0.480
	YES	21	52.5%	19	47.5%	
Cosmetics use	NO	14	48.3%	15	51.7%	0.774
	YES	11	52.4%	10	47.6%	

	LogFC_T_N4BP2L1_cut						
	Low			High			P-value
	Valid N	Standard Deviation	Mean	Valid N	Standard Deviation	Mean	
Age (years)	25	9	51	25	10	53	0.553
BMI	25	3	27	25	4	28	0.256

Correlations

			Age (years)	BMI	LogFC.T.N4BP2 L1
Spearman's rho	Age (years)	Correlation Coefficient	1.000	-.052	.107
		Sig. (2-tailed)	.	.721	.461
		N	50	50	50
	BMI	Correlation Coefficient	-.052	1.000	-.009
		Sig. (2-tailed)	.721	.	.951
		N	50	50	50
	LogFC.T.N4BP2L1	Correlation Coefficient	.107	-.009	1.000
		Sig. (2-tailed)	.461	.951	.
		N	50	50	50

Panel C. All clinical data of our patients and correlation between *BEGAIN* and patient status.

	Valid N	Mean	Standard Deviation	Median	P-value
ΔΔN_BEGAIN	50	-.275	1.256	.079	0.263
ΔΔT_BEGAIN	50	.953	1.696	.974	
LogFC_N_BEGAIN	50	.0828	.3780	-.0239	<0.001
LogFC_T_BEGAIN	50	-.2869	.5104	.-.2933	

		LogFC.T.BEGAIN				
		Valid N	Mean	Standard Deviation	Median	P-value
Age cutpoint	<50	21	-.4042	.5359	-.4486	0.169
	≥50	29	-.2019	.4826	-.2104	
	<2.5	30	-.2931	.5678	-.2570	0.945

Tumor size cutpoint	≥2.5	20	-.2775	.4237	-.2933	
Estrogen receptor	negative	5	-.2396	.6307	-.2403	0.961
	positive	45	-.2921	.5036	-.2976	
Progesterone receptor	negative	7	-.2798	.5304	-.2403	0.989
	positive	43	-.2880	.5135	-.2976	
HER-2 receptor	negative	34	-.2571	.5192	-.2254	0.479
	positive	16	-.3501	.5016	-.3219	
Nuclear grade	1	8	-.2534	.4345	-.2437	0.066
	2	34	-.2605	.5499	-.2751	
	3	8	-.4325	.4245	-.5668	
Histologic grade	1	12	-.1873	.4704	-.1924	0.258
	2	23	-.3382	.5548	-.3036	
	3	15	-.2877	.4912	-.3010	
Lymph nodes metastasis	NO	34	-.2706	.5657	-.2045	0.670
	YES	16	-.3214	.3807	-.3219	
Histologic type of invasive carcinoma	ILC	1	-.2403	.	-.2403	0.862
	IDC	49	-.2878	.5156	-.2976	
Marital status	married	48	-.3094	.4974	-.2993	0.216
	single	2	.2533	.7277	.2533	
Cutpoint of BMI	≤25	15	-.1760	.5157	-.1319	0.971
	25-29	23	-.2720	.5133	-.2976	
	≥30	12	-.4538	.4972	-.5794	
Age of menarch	<14	29	-.3307	.5522	-.3401	0.437
	≥14	21	-.2263	.4523	-.1952	
Is menstruation regular?	NO	5	-.5220	.4230	-.3036	0.340
	YES	45	-.2607	.5166	-.2612	
Age of menpause	<50	27	-.4017	.4501	-.3401	0.069
	≥50	23	-.1520	.5528	-.0958	
	pre	11	-.4175	.3852	-.3401	0.261

Menopausal status	post	39	-.2500	.5390	-.2403	
History of uterine surgery	NO	47	-.2993	.5074	-.2890	0.638
	YES	3	-.0924	.6318	-.3010	
Number of pregnancy	≤3	34	-.2434	.5289	-.2794	0.394
	>3	16	-.3792	.4713	-.2950	
Age at FFTP	<25	33	-.2718	.5175	-.2403	0.464
	≥25	12	-.4027	.5740	-.5102	
Breastfeeding duration	0	4	-.0598	.2542	-.0568	0.270
	<24	26	-.2666	.5656	-.3219	
	≥24	20	-.3586	.4730	-.2950	
Number of abortion	0	34	-.2246	.4956	-.2195	0.256
	1	13	-.3658	.5675	-.3401	
	>1	3	-.6497	.3055	-.8069	
Abortion history	NO	34	-.2246	.4956	-.2195	0.126
	YES	16	-.4191	.5322	-.4668	
Family history of cancer	NO	23	-.3193	.3802	-.2612	0.755
	YES	27	-.2592	.6057	-.3010	
Diseases history	NO	26	-.2753	.5650	-.2794	0.360
	LOCUS	1	-.0958	.	-.0958	
	Thyroid problem	2	.2336	.7598	.2336	
	blood pressure	11	-.4582	.4437	-.5654	
	blood fat	6	-.2581	.4263	-.2195	
	heart problem	1	-.1319	.	-.1319	
	Diabetes	3	-.2786	.4439	-.0931	
Chest radiograph history	NO	21	-.2150	.6293	-.3010	0.575
	YES	29	-.3389	.4077	-.2890	
Hormone therapy history	NO	48	-.2998	.5023	-.2933	0.488
	YES	2	.0228	.8410	.0228	
Duration of OCP consumption	0	28	-.2604	.4413	-.2751	0.928
	≤5	16	-.2611	.5685	-.2902	
	>5	6	-.4788	.6935	-.7356	

OCP consumption	NO	28	-.2604	.4413	-.2751	0.681
	YES	22	-.3205	.5960	-.3943	
Vitamin D consumption	NO	27	-.3609	.5296	-.3036	0.340
	YES	23	-.2000	.4838	-.2403	
Regular sleep	NO	19	-.3455	.5065	-.3036	0.522
	YES	31	-.2509	.5177	-.2104	
Exercising	NO	33	-.3620	.5150	-.3401	0.149
	YES	17	-.1410	.4827	-.1952	
Deodorant use	NO	33	-.3235	.4890	-.2612	0.830
	YES	17	-.2157	.5580	-.3010	
Hair dye use	NO	10	-.2807	.5486	-.2633	0.865
	YES	40	-.2884	.5078	-.2933	
Cosmetics use	NO	29	-.3581	.5291	-.3401	0.275
	YES	21	-.1885	.4782	-.2403	

		LogFC_T_BEGAIN_cut				
		Low		High		
		Count	Row N %	Count	Row N %	P-value
Age_cutpoint	<50	13	62%	8	38%	0.288
	≥50	12	41%	17	58%	
Tumor size_cutpoint	<2.5	15	50%	15	50%	0.458
	≥2.5	10	50%	10	50%	
Estrogen receptor	negative	2	40%	3	60%	0.823
	positive	23	51%	22	49%	
Progesterone receptor	negative	3	43%	4	57%	0.824
	positive	22	51%	21	49%	
HER-2 receptor	negative	14	41%	20	59%	0.041
	positive	11	69%	5	31%	
	1	4	50%	4	50%	0.910

Nuclear grade	2	16	47%	18	53%	
	3	5	63%	3	38%	
Histologic grade	1	5	42%	7	58%	0.554
	2	12	52%	11	48%	
	3	8	53%	7	47%	
Lymph nodes metastasis	NO	15	44%	19	56%	0.418
	YES	10	63%	6	38%	
Histologic type of invasive carcinoma	ILC	0	0%	1	100%	0.575
	IDC	25	51%	24	49%	
Marital status	married	25	52%	23	48%	0.324
	single	0	0%	2	100%	
Cutpoint_of_BMI	≤25	5	33%	10	67%	0.304
	25-29	12	52%	11	48%	
	≥30	8	67%	4	33%	
Age of menarche	<14	17	59%	12	41%	0.219
	≥14	8	38%	13	62%	
Is menstruation regular?	NO	4	80%	1	20%	0.364
	YES	21	47%	24	53%	
Age of menopause	<50	17	63%	10	37%	0.099
	≥50	8	35%	15	65%	
Menopausal status	pre	7	64%	4	36%	0.548
	post	18	46%	21	54%	
History of uterine surgery	NO	23	49%	24	51%	0.825
	YES	2	67%	1	33%	
Number of pregnancy	≤3	17	50%	17	50%	0.331
	>3	8	50%	8	50%	
Age at FFTP	<25	16	48%	17	51%	0.734
	≥25	7	58%	5	42%	
	0	1	25%	3	75%	0.681

Breastfeeding duration	<24	14	54%	12	46%	
	≥24	10	50%	10	50%	
Number of abortion	0	15	44%	19	56%	0.150
	1	7	54%	6	46%	
	>1	3	100%	0	0%	
Abortion history	NO	15	44%	19	56%	0.120
	YES	10	63%	6	37%	
Family history of cancer	NO	11	48%	12	52%	0.593
	YES	14	52%	13	48%	
Diseases history	NO	13	50%	13	50%	0.002
	LOCUS	0	0%	1	100%	
	Thyroid problem	1	50%	1	50%	
	blood pressure	8	73%	3	27%	
	blood fat	2	33%	4	67%	
	heart problem	0	0%	1	100%	
	Diabetes	1	33%	2	67%	
Chest radiograph history	NO	11	52%	10	48%	0.445
	YES	14	48%	15	52%	
Hormone therapy history	NO	24	50%	24	50%	0.979
	YES	1	50%	1	50%	
Duration of OCP consumption	0	13	46%	15	54%	0.551
	≤5	8	50%	8	50%	
	>5	4	67%	2	33%	
OCP consumption	NO	13	46%	15	54%	0.397
	YES	12	55%	10	46%	
Vitamin D consumption	NO	15	56%	12	44%	0.429
	YES	10	43%	13	56%	
Regular sleep	NO	12	63%	7	37%	0.291
	YES	13	42%	18	58%	
Exercising	NO	19	58%	14	42%	0.159
	YES	6	35%	11	64%	

Deodorant use	NO	16	48%	17	52%	0.327
	YES	9	53%	8	47%	
Hair dye use	NO	5	50%	5	50%	0.878
	YES	20	50%	20	50%	
Cosmetics use	NO	17	59%	12	41%	0.219
	YES	8	38%	13	62%	

	LogFC_T_BEGAIN_cut						
	Low			High			P-value
	Valid N	Standard Deviation	Mean	Valid N	Standard Deviation	Mean	
Age (years)	25	8	50	25	11	54	0.943
BMI	25	4	28	25	3	27	0.058

Correlations

			Age (years) <50 or ≥50	BMI	LogFC.T.BEGAIN
Spearman's rho	Age (years)	Correlation Coefficient	1.000	-.052	.121
		Sig. (2-tailed)	.	.721	.404
		N	50	50	50
	BMI	Correlation Coefficient	-.052	1.000	-.224
		Sig. (2-tailed)	.721	.	.118
		N	50	50	50
	LogFC.T.BEGAIN	Correlation Coefficient	.121	-.224	1.000
		Sig. (2-tailed)	.404	.118	.
		N	50	50	50
