# Supplementary material for: Involvement of N4BP2L1, PLEKHA4, and BEGAIN genes in breast cancer and muscle cell development
Source: Front Cell Dev Biol. 2024 May 24;12:1295403. doi: 10.3389/fcell.2024.1295403 (PMC11163233; doi:10.3389/fcell.2024.1295403)

Supplementary Figure S2. The protein expression of *N4BP2L1*, *PLEKHA4*, and *BEGAIN* in TCGA - BRCA, samples from the CPTAC dataset, and normal breast tissues from the GTEx database.

Panel A. N4bp2l1 protein expression in TCGA-BRCA samples.
12 out of 18 samples showed weak expression and 6 samples had moderate expression.
The following samples showed weak expressions.

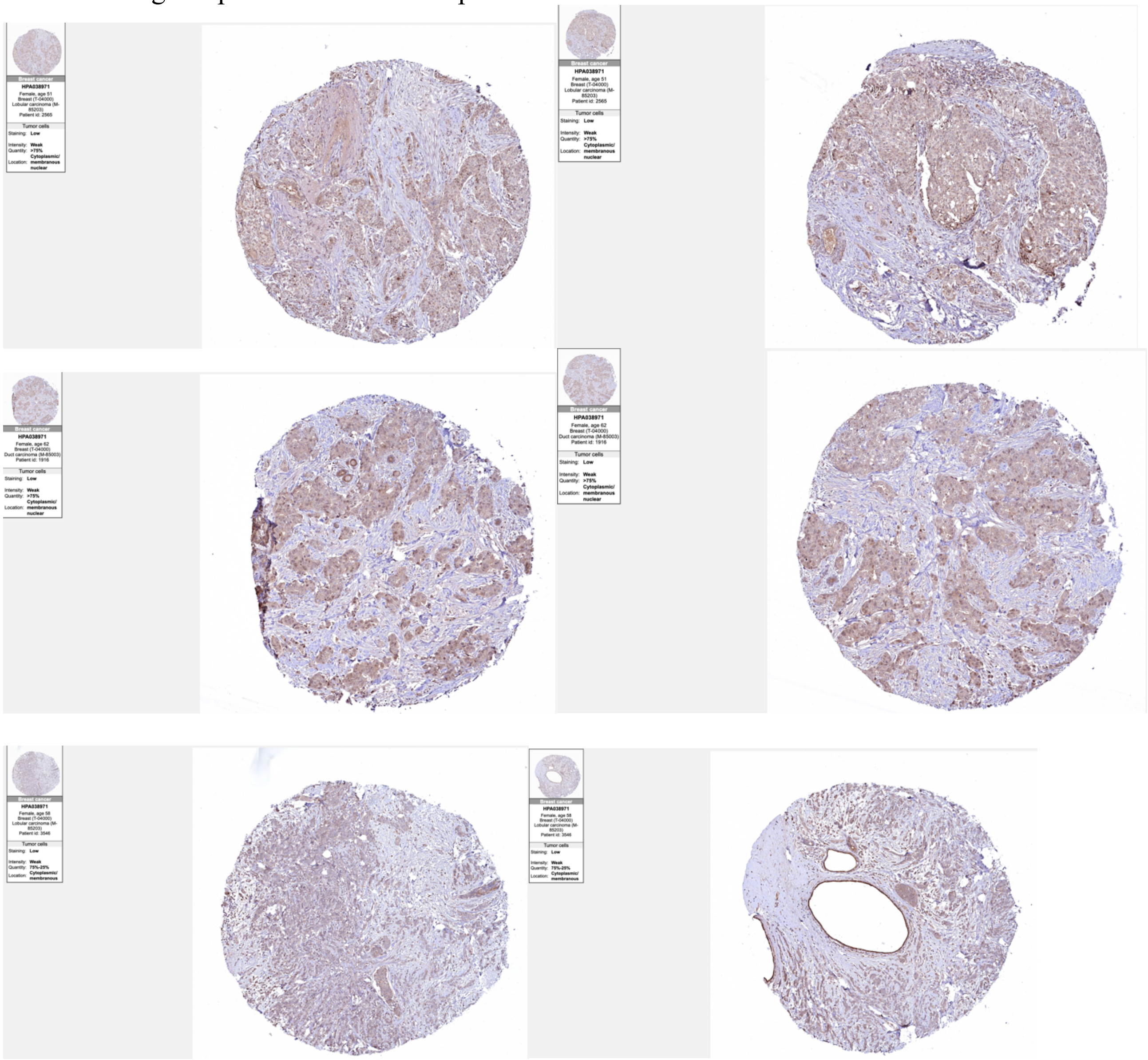

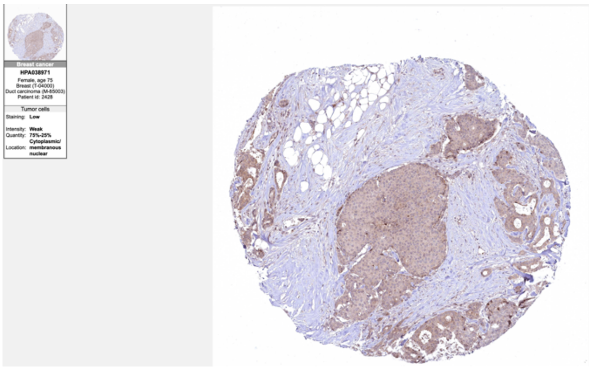
Breast cancer
HPA038971
Female, age 75
Breast (T-04000)
Duct carcinoma (M-85003)
Patient id: 2428
Tumor cells
Staining: Low
Intensity: Weak
Quantity: 75%-25%
Location: Cytoplasmic/ membranous nuclear

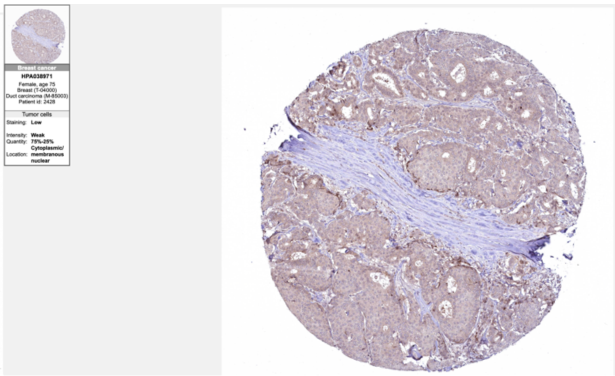
Breast cancer
HPA038971
Female, age 75
Breast (T-04000)
Duct carcinoma (M-85003)
Patient id: 2428
Tumor cells
Staining: Low
Intensity: Weak
Quantity: 75%-25%
Location: Cytoplasmic/ membranous nuclear

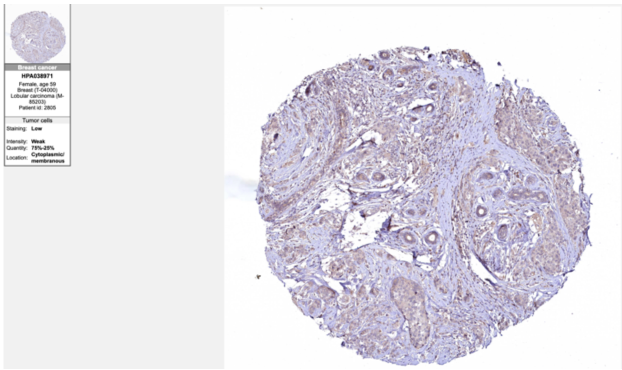
Breast cancer
HPA038971
Female, age 59
Breast (T-04000)
Lobular carcinoma (M-85203)
Patient id: 2805
Tumor cells
Staining: Low
Intensity: Weak
Quantity: 75%-25%
Location: Cytoplasmic/ membranous

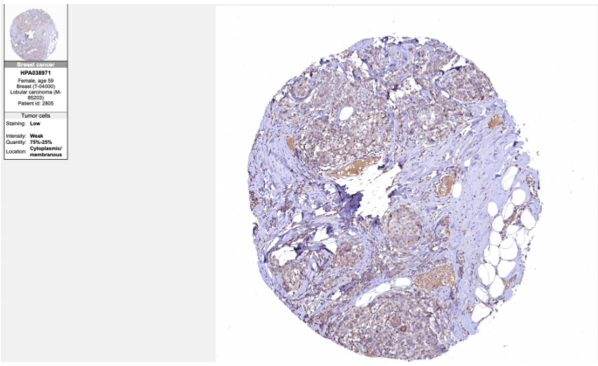
Breast cancer
HPA038971
Female, age 59
Breast (T-04000)
Lobular carcinoma (M-85203)
Patient id: 2805
Tumor cells
Staining: Low
Intensity: Weak
Quantity: 75%-25%
Location: Cytoplasmic/ membranous

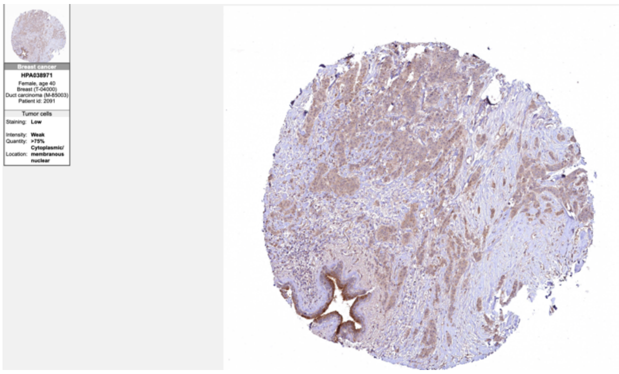
Breast cancer
HPA038971
Female, age 40
Breast (T-04000)
Duct carcinoma (M-85003)
Patient id: 2091
Tumor cells
Staining: Low
Intensity: Weak
Quantity: >75%
Location: Cytoplasmic/ membranous nuclear

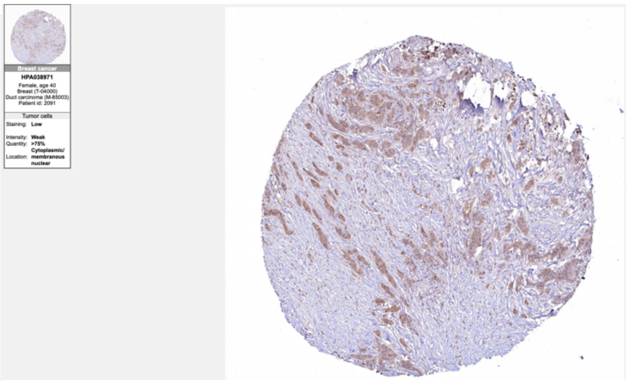
Breast cancer
HPA038971
Female, age 40
Breast (T-04000)
Duct carcinoma (M-85003)
Patient id: 2091
Tumor cells
Staining: Low
Intensity: Weak
Quantity: >75%
Location: Cytoplasmic/ membranous nuclear

The following samples showed moderate expression.

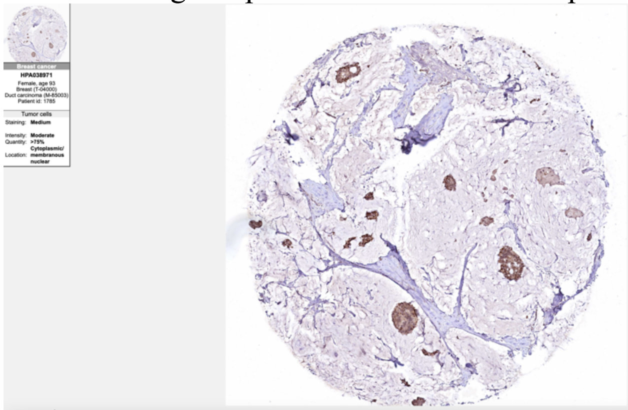

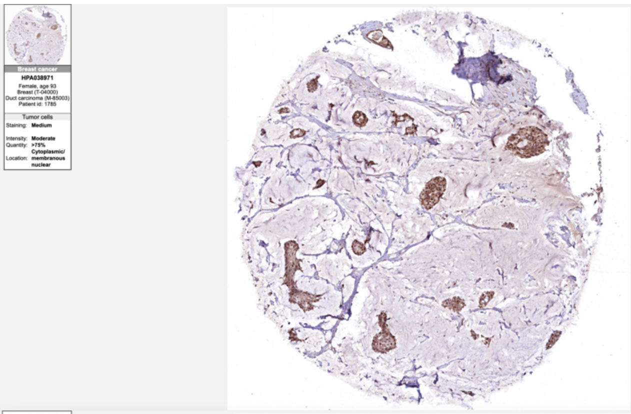

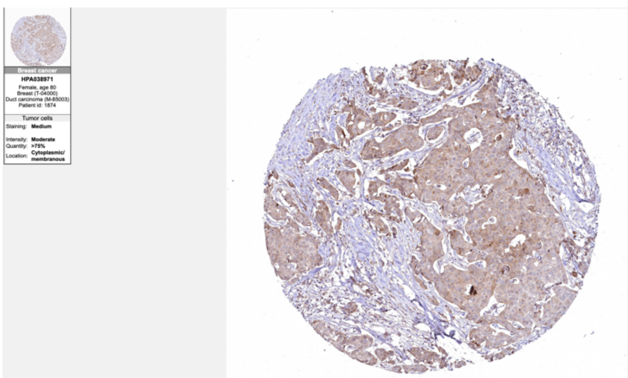

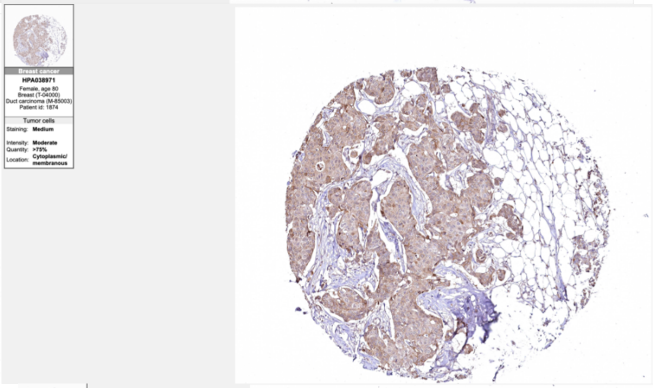

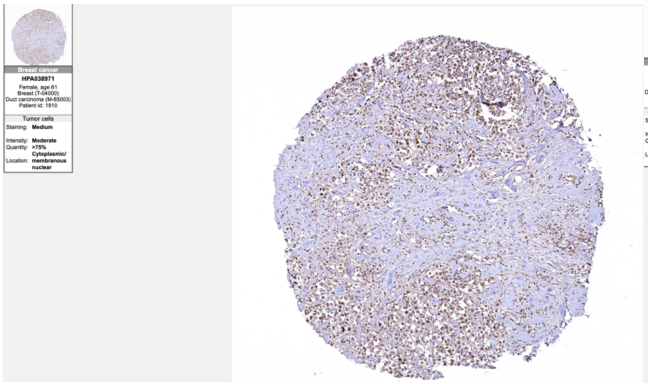

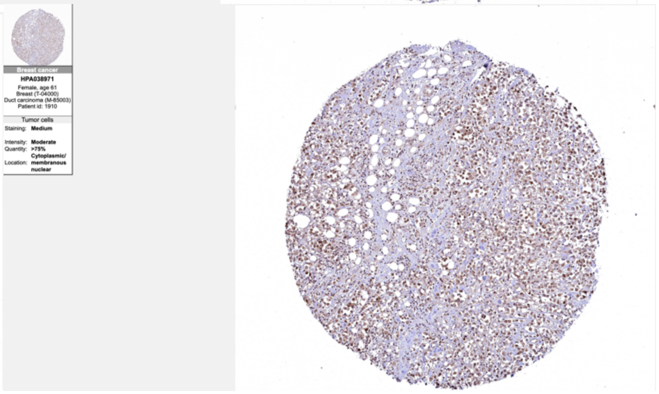

Panel B. Plekha4 protein expression in TCGA-BRCA samples.
Expression quality of less than 25% was excluded.
Here only one sample with a weak plekha4 protein expression is shown. Other 29 samples showed moderate or strong.

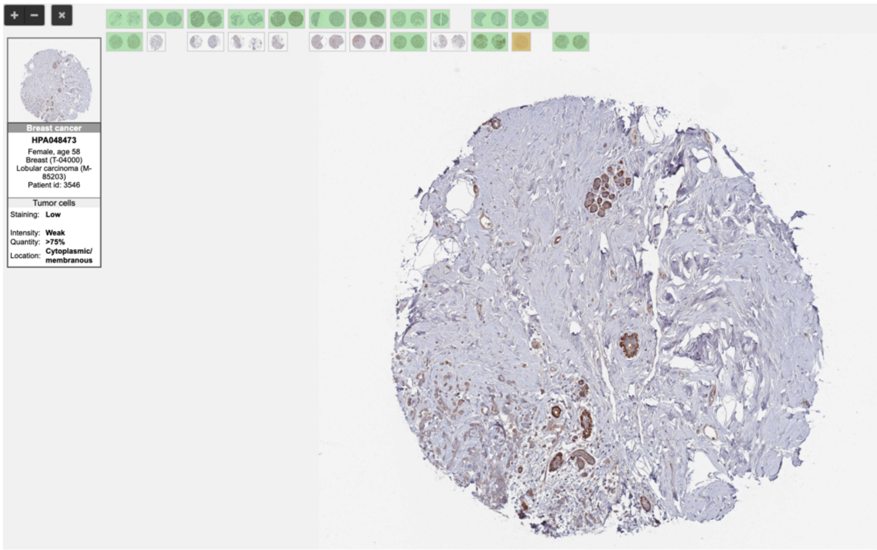

Panel C. Begain protein expression in TCGA-BRCA samples.
Expression quality of less than 25% was excluded.
All samples had moderate intensity for BEGAIN protein expression.

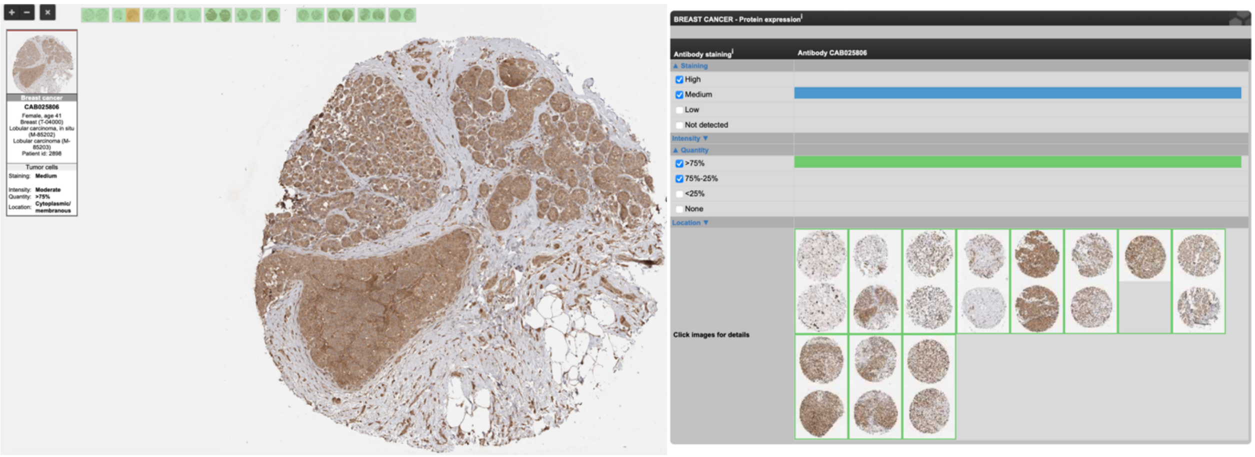

Panel D. N4bp2l1 protein expression in samples from CPTAC datasets.

N4bp2l1 protein expression in breast cancer

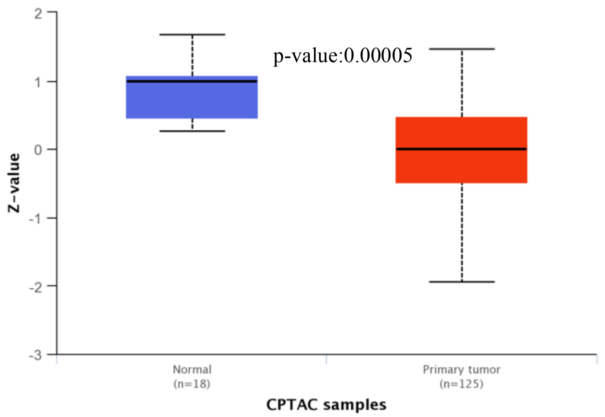

*Z-values: standard deviations from the median across samples for the specific tumor type.

Panel E. N4bp2l1 protein expression in breast cancer samples of CPTAC datasets based on patient's statuses.

N4bp2l1 proteomic expression profile based on individual cancer stages

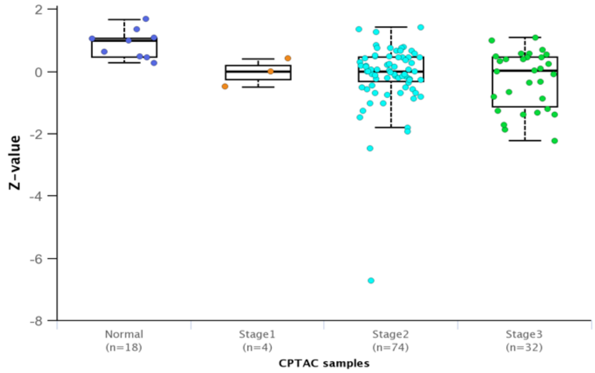

N4bp2l1 proteomic expression profile based on major subclasses

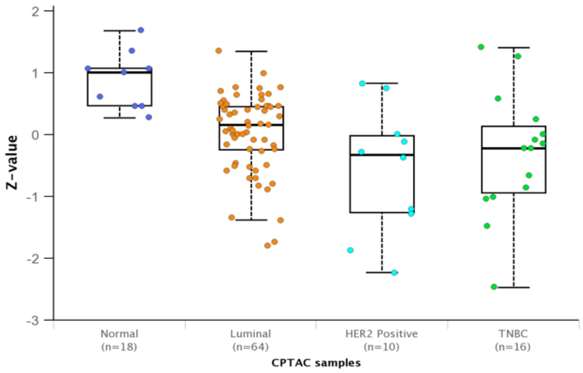

Comparison	[#]Statistical significance
[##]N-vs-[###]S1	0.046
N-vs-S2	3.5E-05
N-vs-S3	2.4E-05

Comparison	Statistical significance
N-vs-Luminal	2.6E-04
N-vs-HER2 Positive	1.5E-03
N-vs-TNBC	7.14E-04

[#]Only significant differential expressions are given.
[##]N: Normal
[###]S: Stage

N4bp2l1 proteomic expression profile based on tumor histology

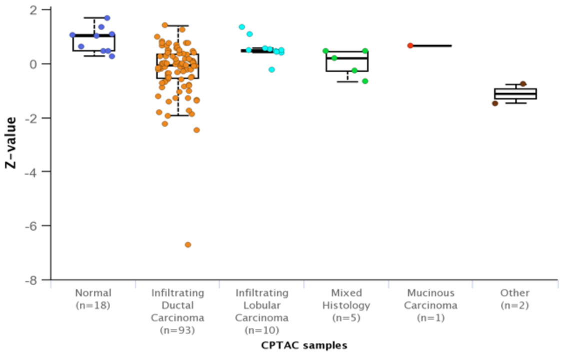

Comparison	Statistical significance
N-vs- Infiltrating Ductal Carcinoma	9.3E-06
N-vs- Mixed histology	0.013
Infiltrating Ductal Carcinoma-vs- Infiltrating Lobular Carcinoma	6.1E-05
Mucinous carcinoma-vs-Other	<1E-12

N4bp2l1 proteomic expression profile based on HIPPO pathway status

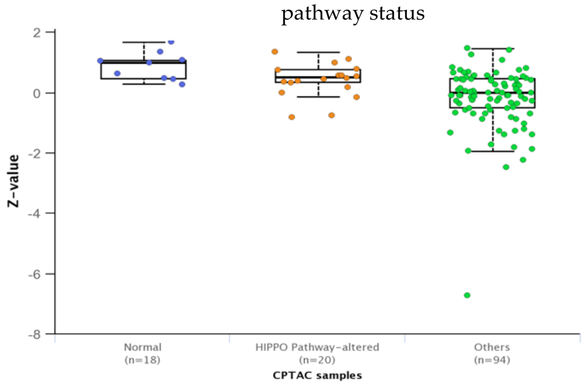

Comparison	Statistical significance
N-vs-HIPPO pathway altered	0.026
N-vs-Others	1.92E-05
HIPPO pathway altered-vs- Others	9.4E-04

N4bp2l1 proteomic expression profile based on WNT pathway status

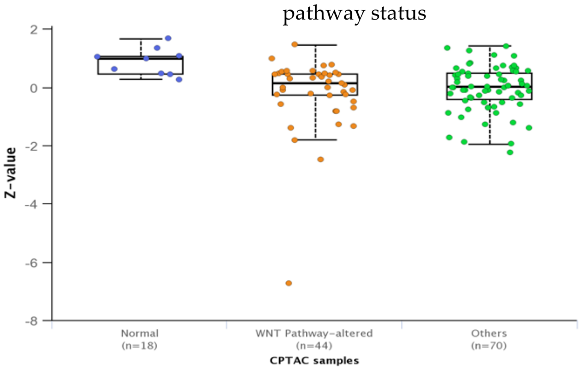

Comparison	Statistical significance
N-vs-WNT pathway altered	1.2E-04
N-vs-Others	1.2E-04

N4bp2l1 proteomic expression profile based on mTOR pathway status

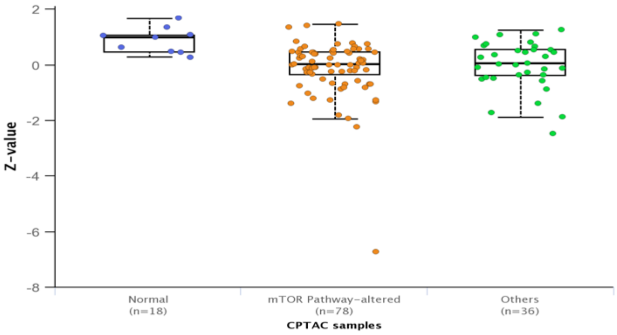

Comparison	Statistical significance
N-vs-mTOR pathway altered	3.7E-05
N-vs-Others	2.8E-04

N4bp2l1 proteomic expression profile based on NRF2 pathway status

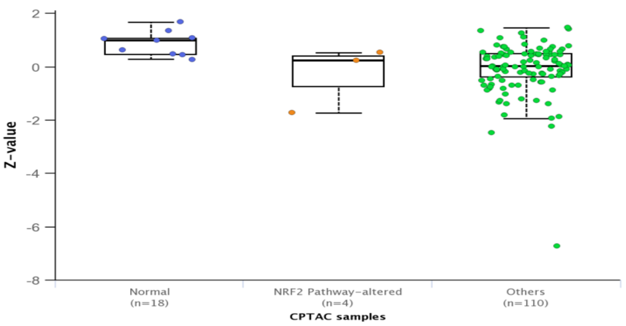

Comparison	Statistical significance
Normal-vs-Others	6.5E-05

N4bp2l1 proteomic expression profile based on WNT pathway status

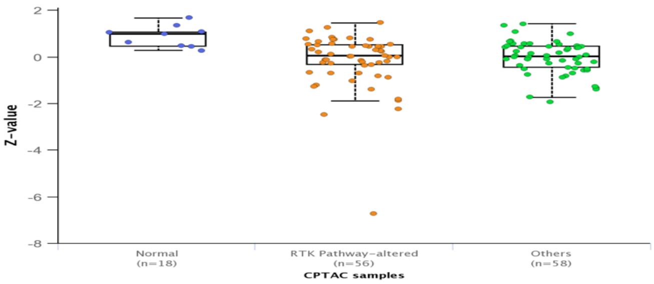

Comparison	Statistical significance
Normal-vs-RTK pathway altered	4.6E-05
Normal-vs-Others	1.53E-04

N4bp2l1 proteomic expression profile based on p53/Rb-related pathway status

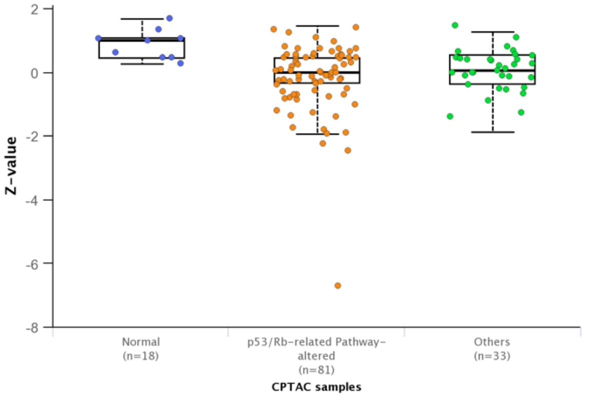

Comparison	Statistical significance
Normal-vs-p53/Rb-related pathway altered	2.21E-05
Normal-vs-Others	5.74E-04

N4bp2l1 proteomic expression profile based on SWI-SNF complex status

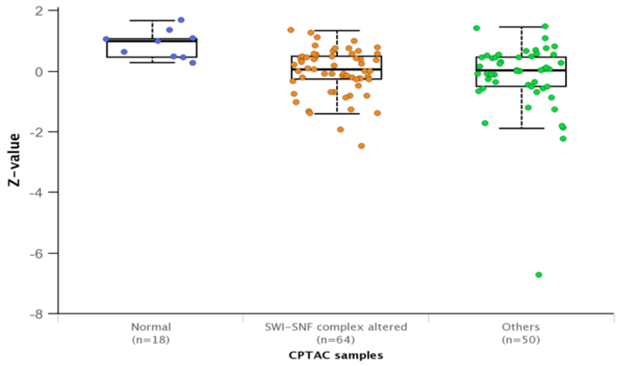

Comparison	Statistical significance
Normal-vs-SWI-SNF complex altered	1.5E-04
Normal-vs-Others	4.8E-05

N4bp2l1 proteomic expression profile based on MYC/MYCN status

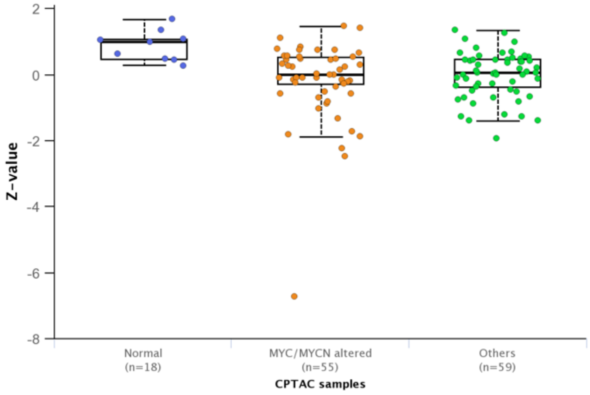

Comparison	Statistical significance
Normal-vs-MYC/MYCN altered	4.3E-05
Normal-vs-Others	1.72E-04

N4bp2l1 proteomic expression profile based on chromatin modifier status

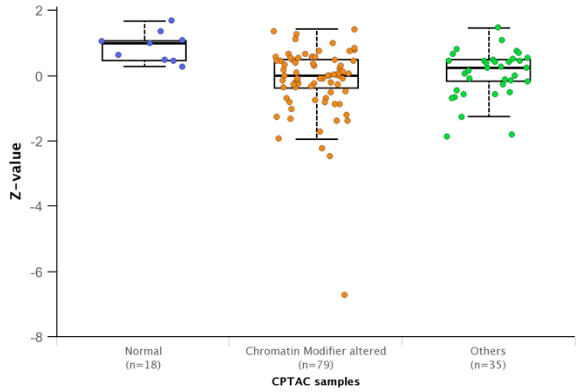

Comparison	Statistical significance
Normal-vs-Chromatin Modifier altered	2.6E-05
Normal-vs-Others	3.86E-04

*UALCAN methodically detects pathway-level somatic alterations in cancers combining data of merged protein, whole-exome sequencing, and Copy-number alterations (CNA), in which it involves main pathways and genes previously annotated in various tumor types on the basis of domain knowledge.

Panel F. N4bp2l1 protein expression in adipocytes, myoepithelial, and glandular cells of normal breast tissues from the GTEx database.

Antibody HPA038971

**Adipocytes cells**	**Not detected**
**Glandular cells**	**Medium**
**Myoepithelial cells**	**Medium**

N4bp2l1

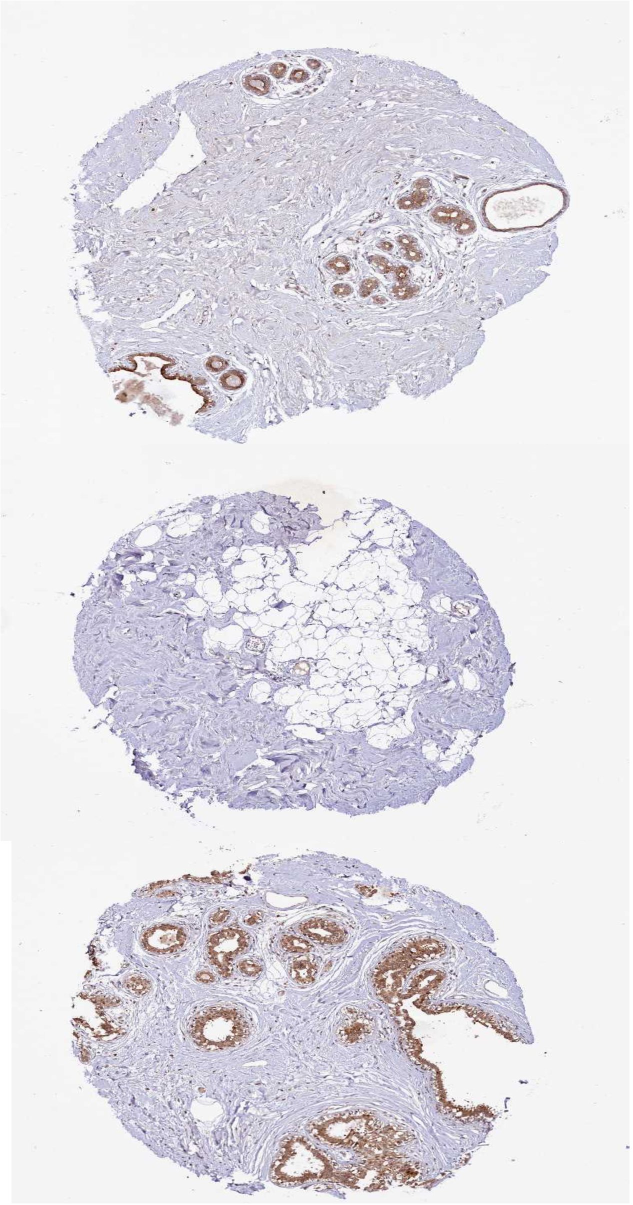

Panel G. Plekha4 protein expression in adipocytes, myoepithelial, and glandular cells of normal breast tissues from the GTEx database.

**Adipocytes cells**	**Not detected**
**Glandular cells**	**Medium**
**Myoepithelial cells**	**High**

Plekha4

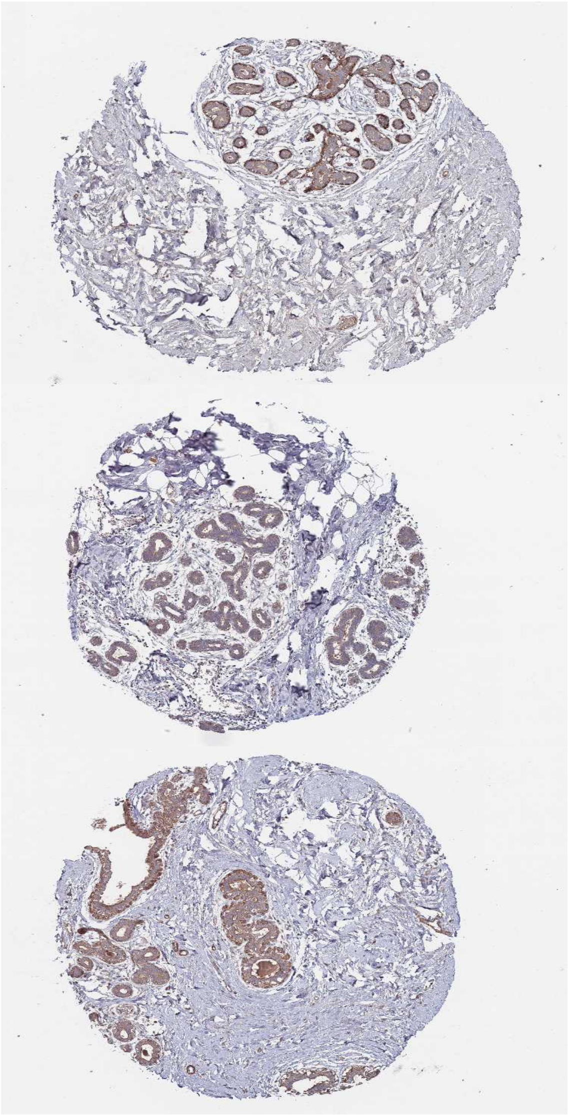

Supplement: Supplementary file 1 [file DataSheet1.zip › Supplementary files/Supplementary Figure S2.pdf]
