# Supplementary material for: Involvement of N4BP2L1, PLEKHA4, and BEGAIN genes in breast cancer and muscle cell development
Source: Front Cell Dev Biol. 2024 May 24;12:1295403. doi: 10.3389/fcell.2024.1295403 (PMC11163233; doi:10.3389/fcell.2024.1295403)

Supplementary Figure S3. | The RNA expression of *N4BP2L1*, *PLEKHA4*, and *BEGAIN* in different cell types of in vivo muscle developmental stages extracted from their scRNA seq data.

Panel.1. Embryonic Development

Embryonic Week 5-6 Hindlimb

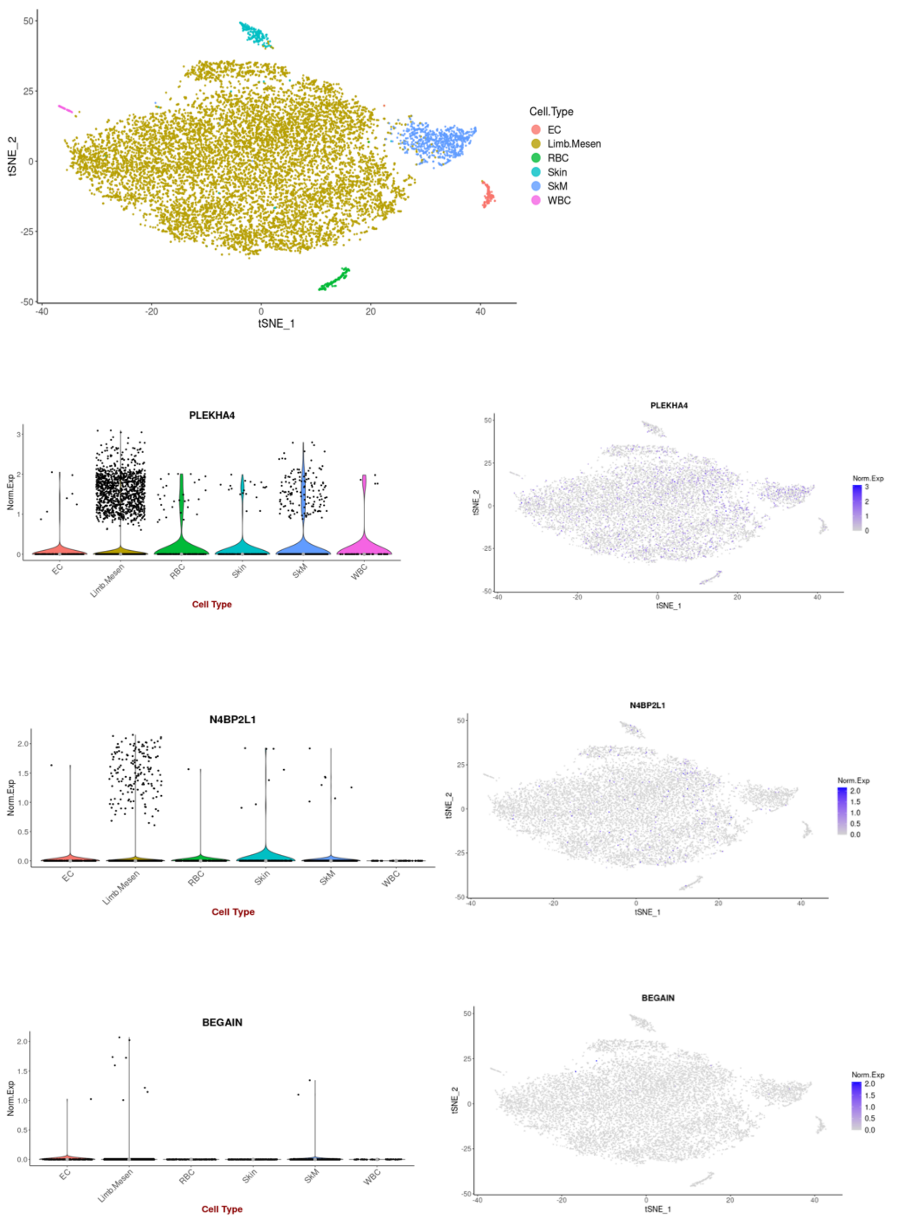

Embryonic Week 5-6 Myogenic Subset

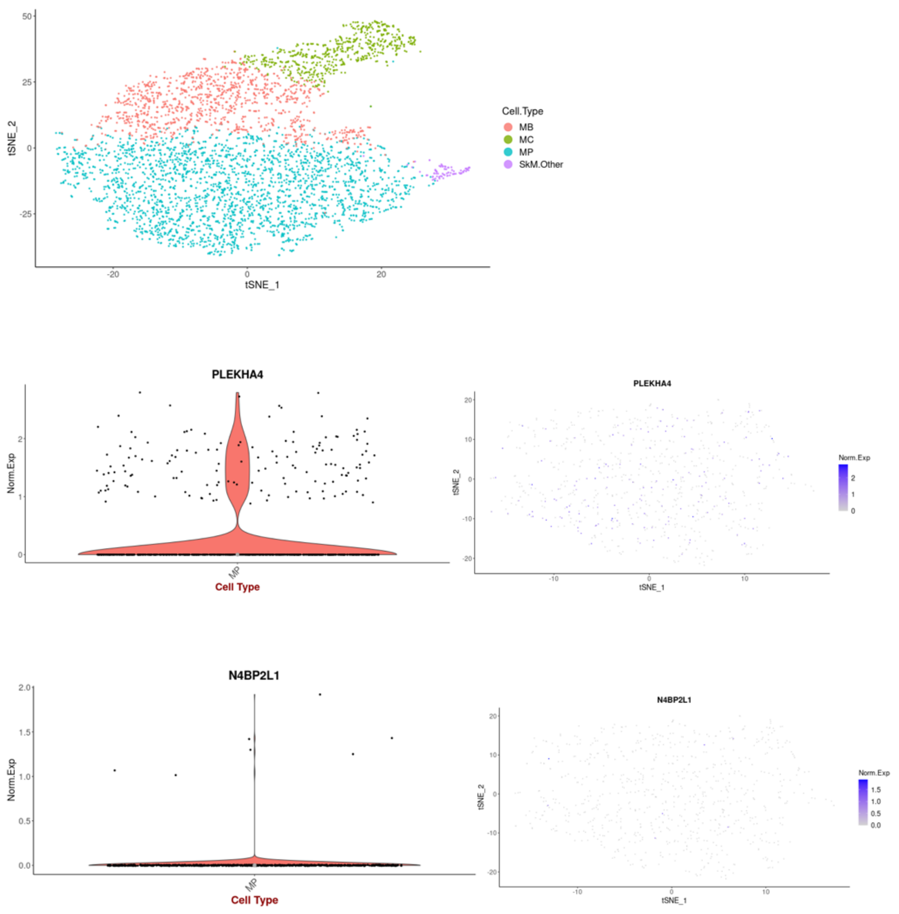

Embryonic Week 6-7 Hindlimb

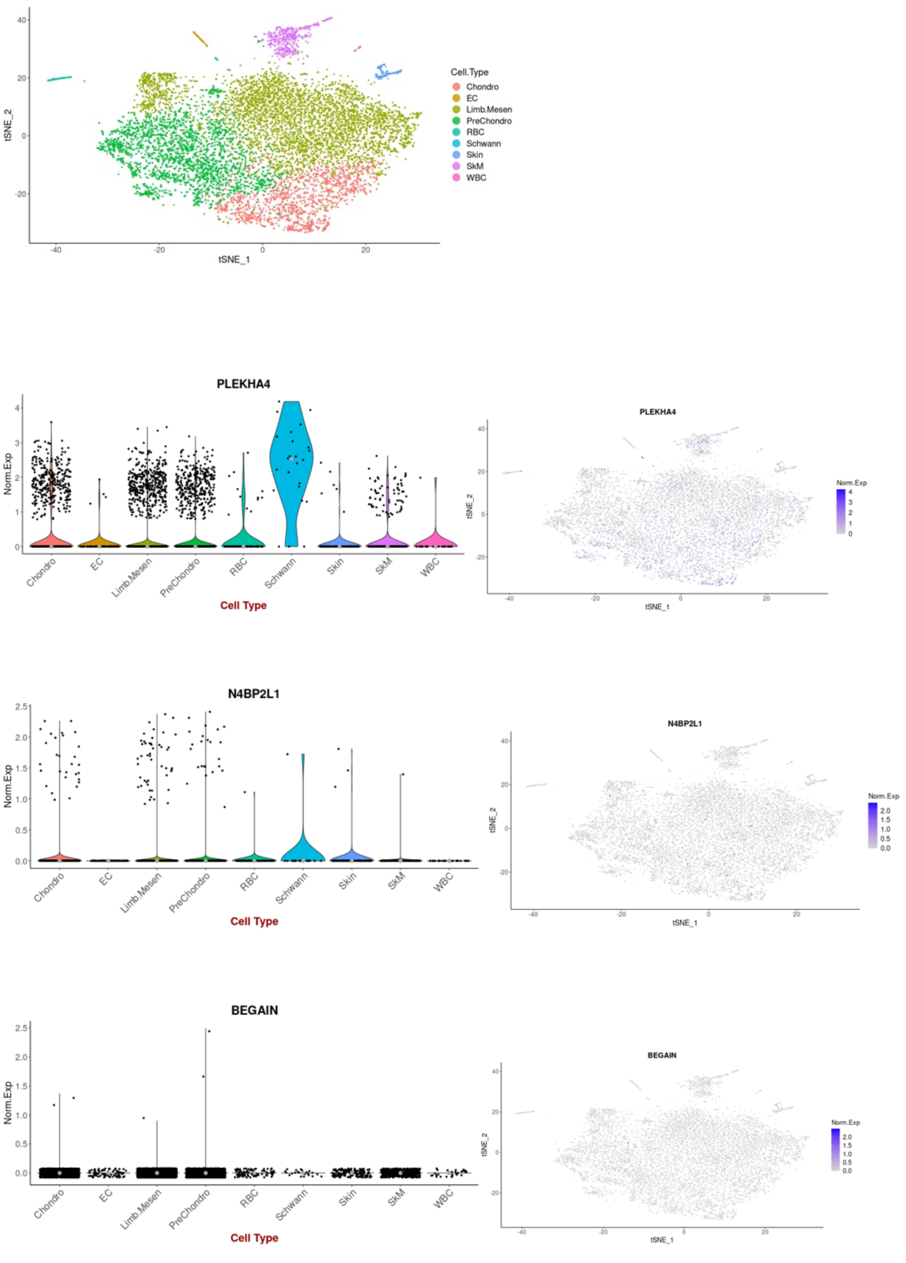

Embryonic Week 6-7 Myogenic Subset

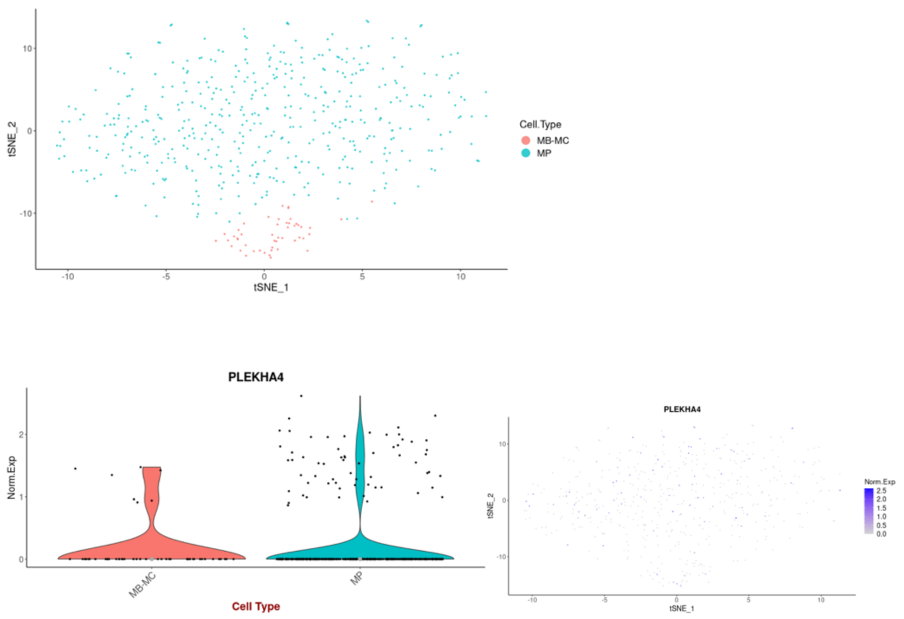

Embryonic Week 7-8 Hindlimb

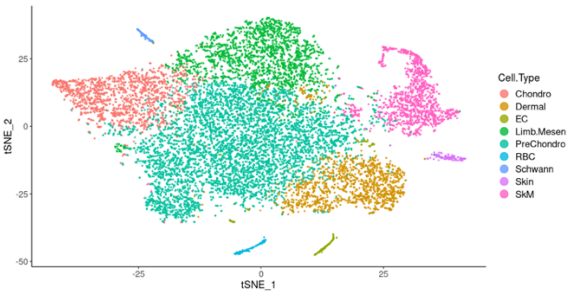

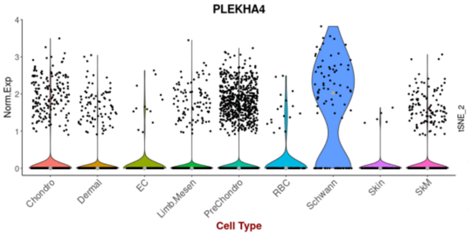

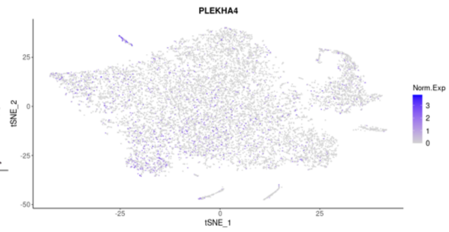

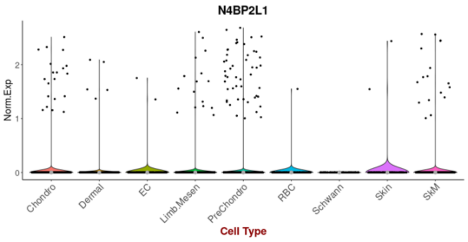

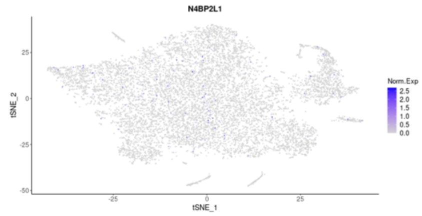

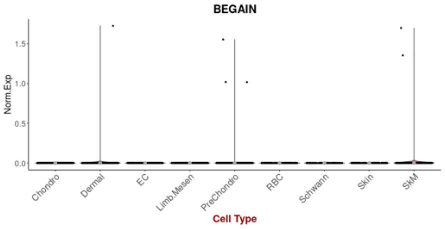

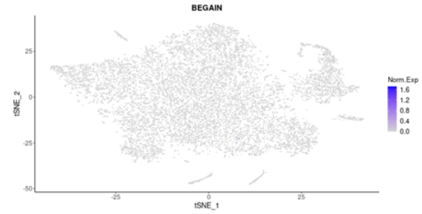

Embryonic Week 7-8 Myogenic Subset

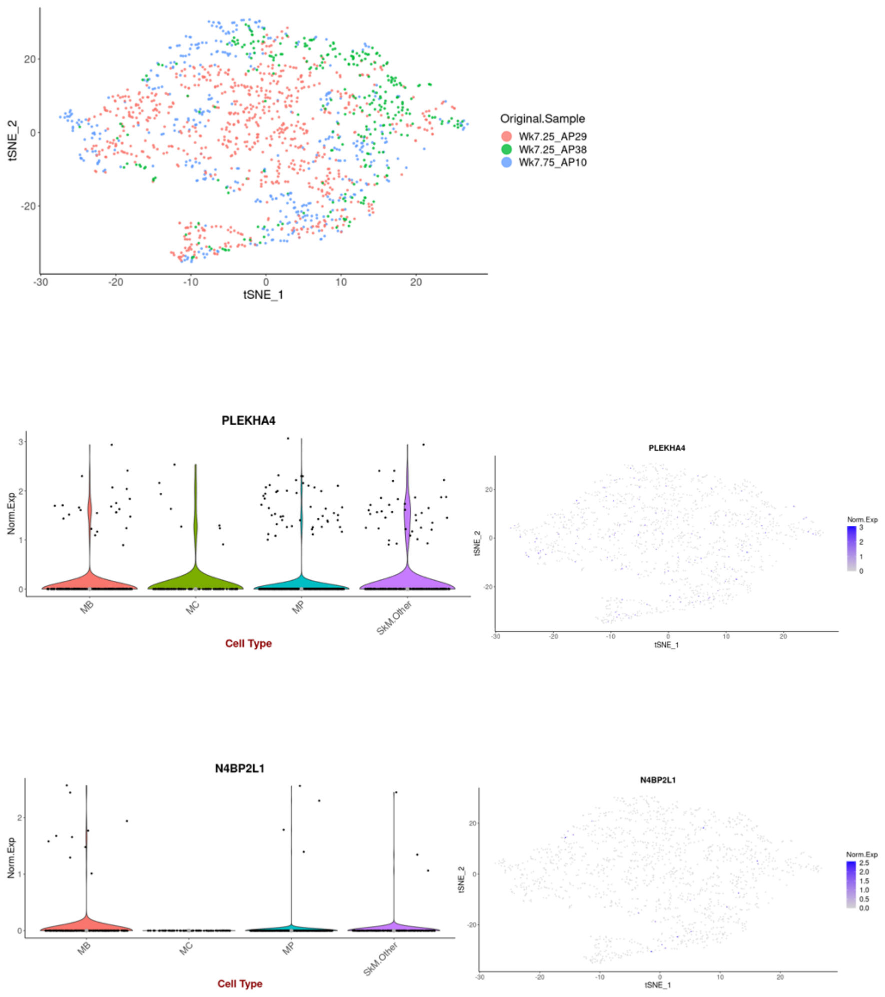

Panel.2. Fetal Development

Fetal Week 09 Hindlimb

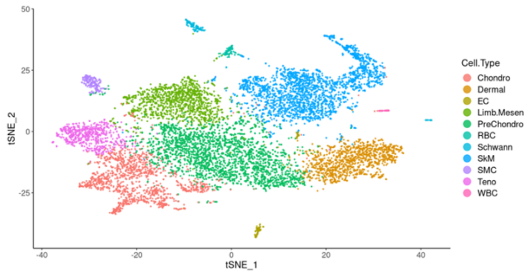

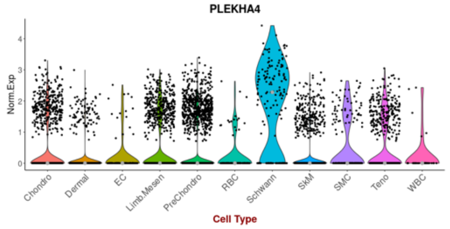

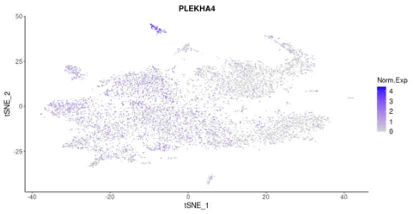

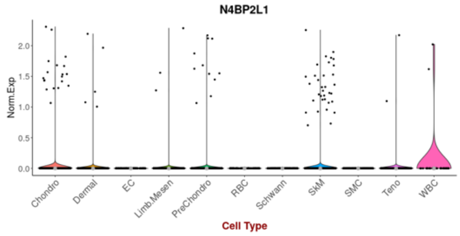

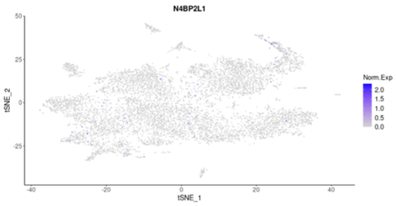

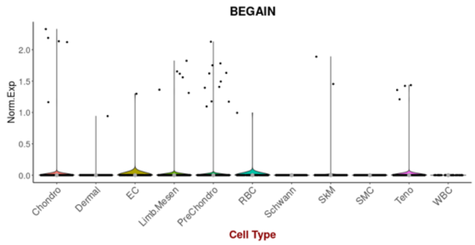

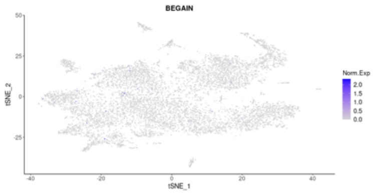

Fetal Week 09 Myogenic Subset

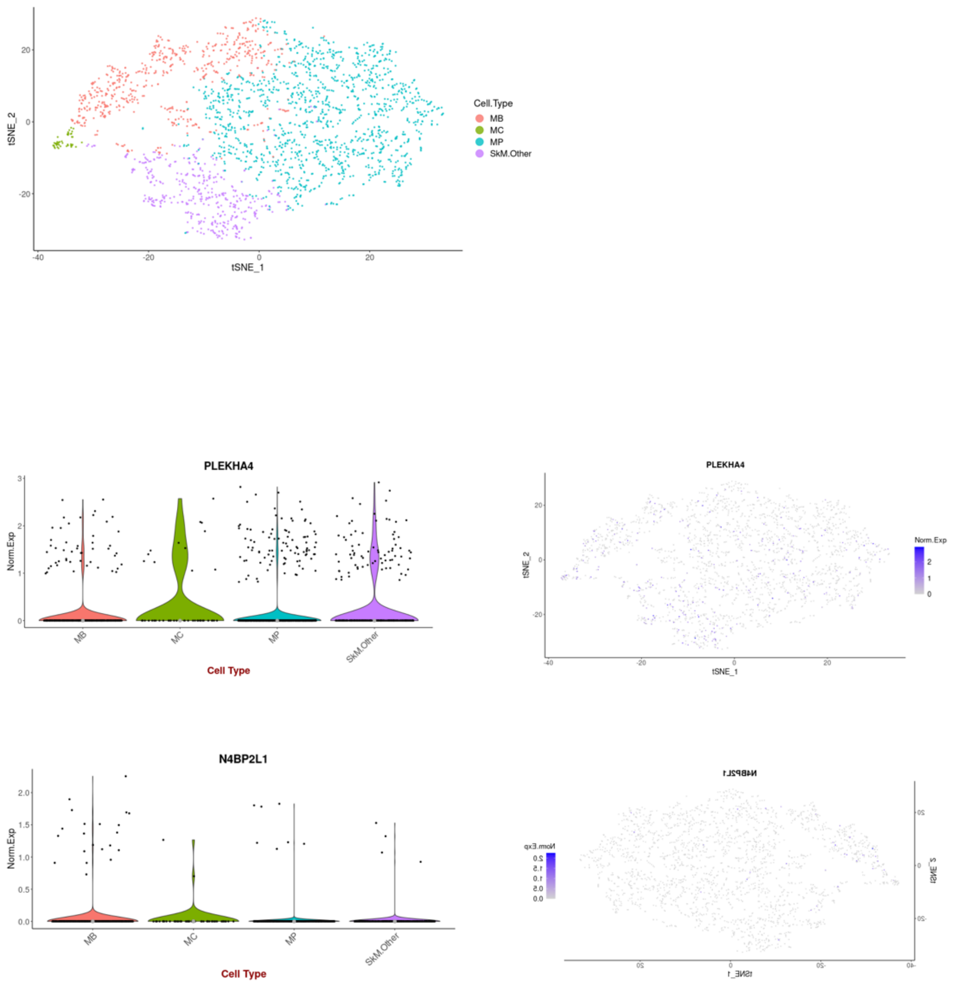

Fetal Week 12-14 Hindlimb Muscle

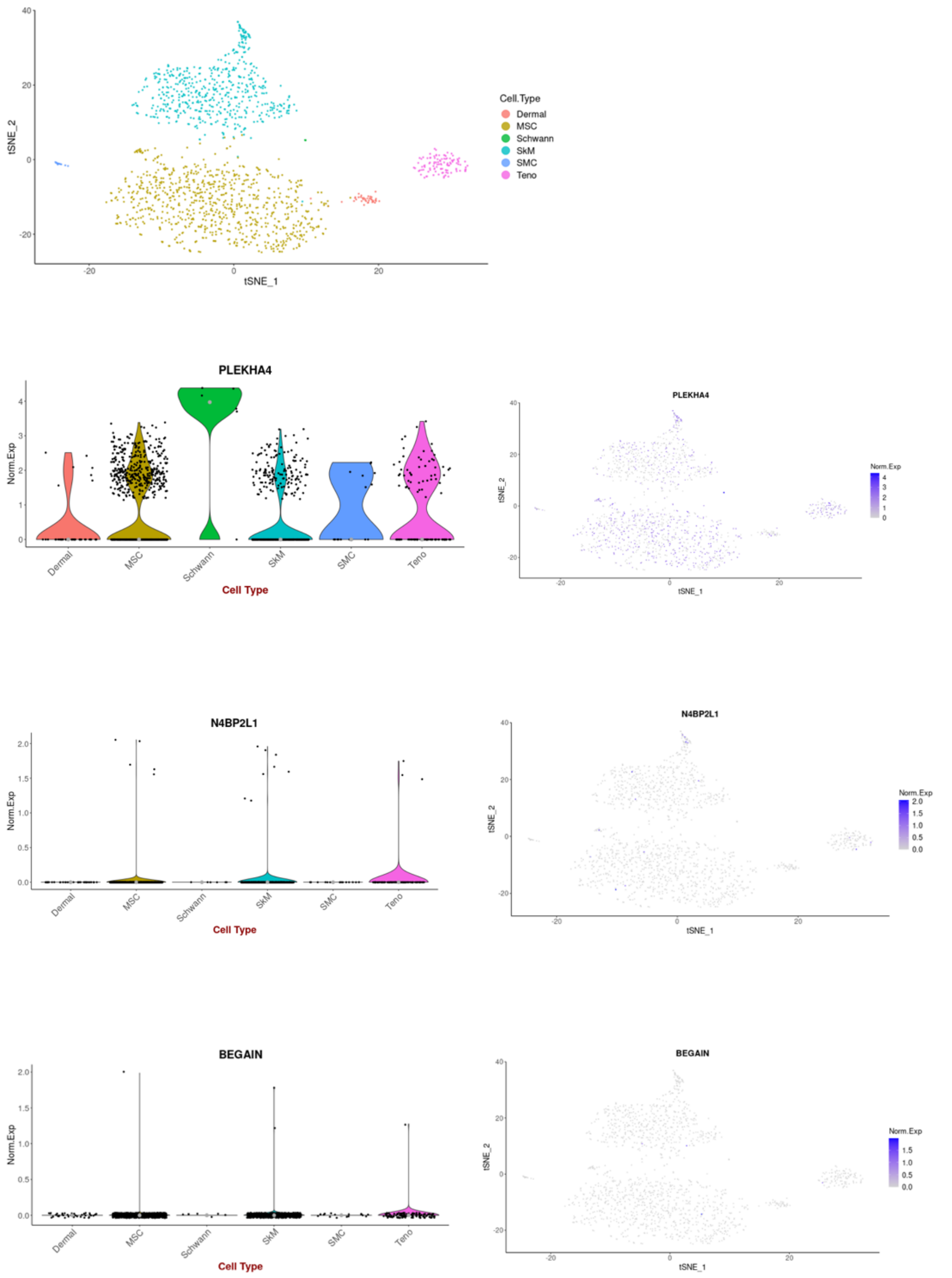

Fetal Week 12-14 Myogenic Subset

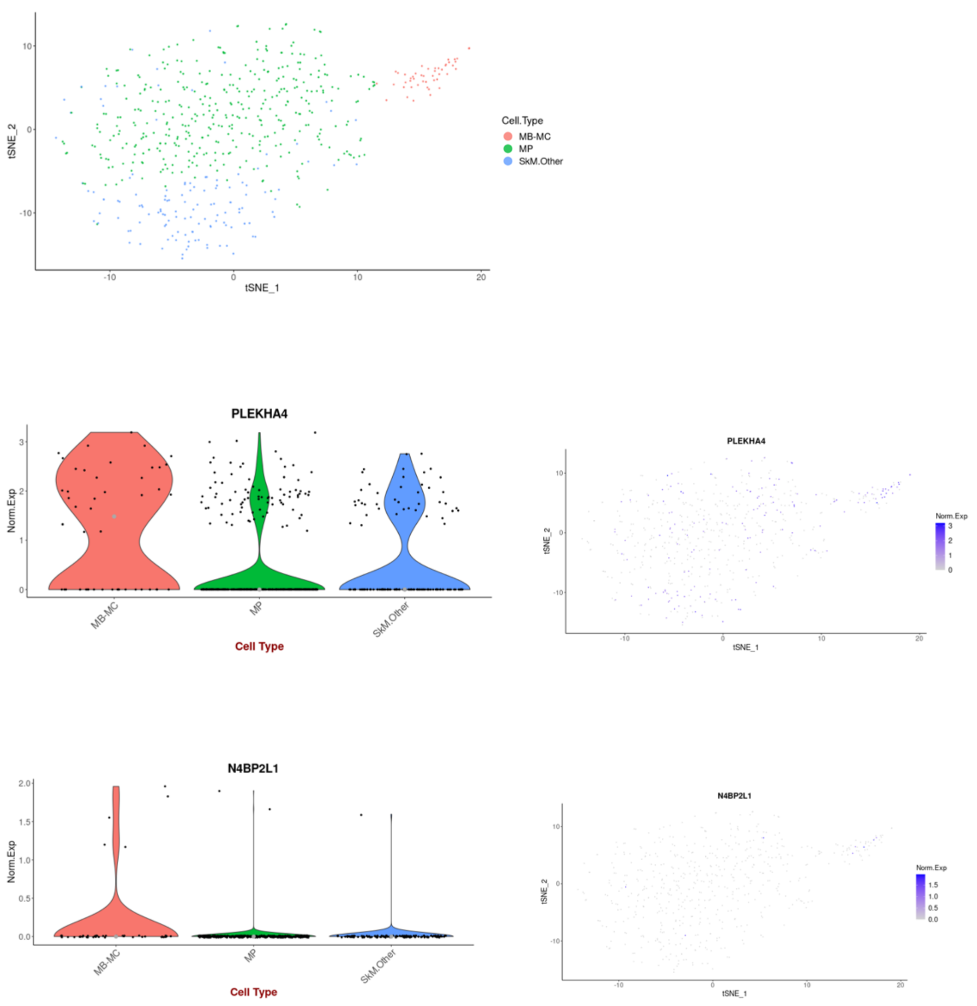

Fetal Week 17-18 Hindlimb Muscle

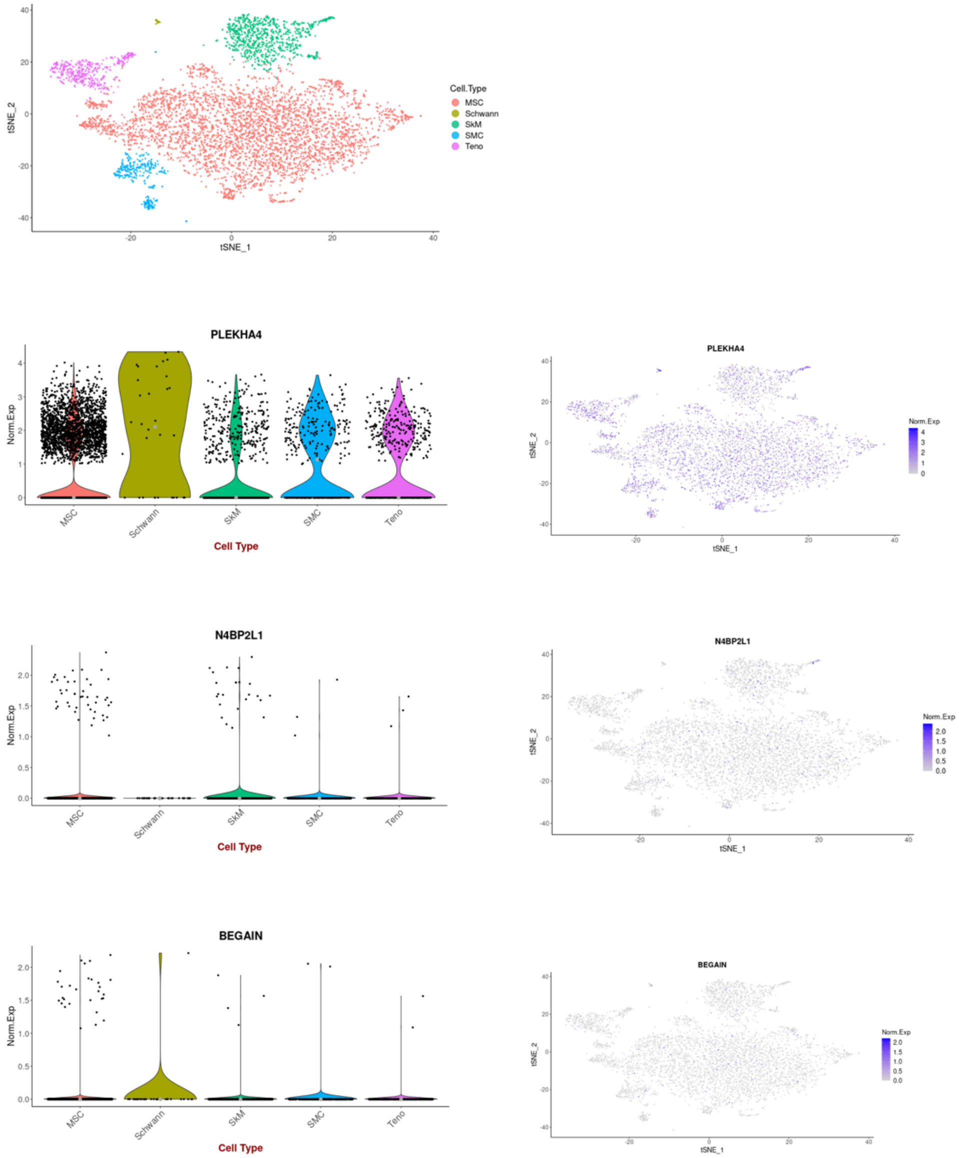

Fetal Week 17-18 Myogenic Subset

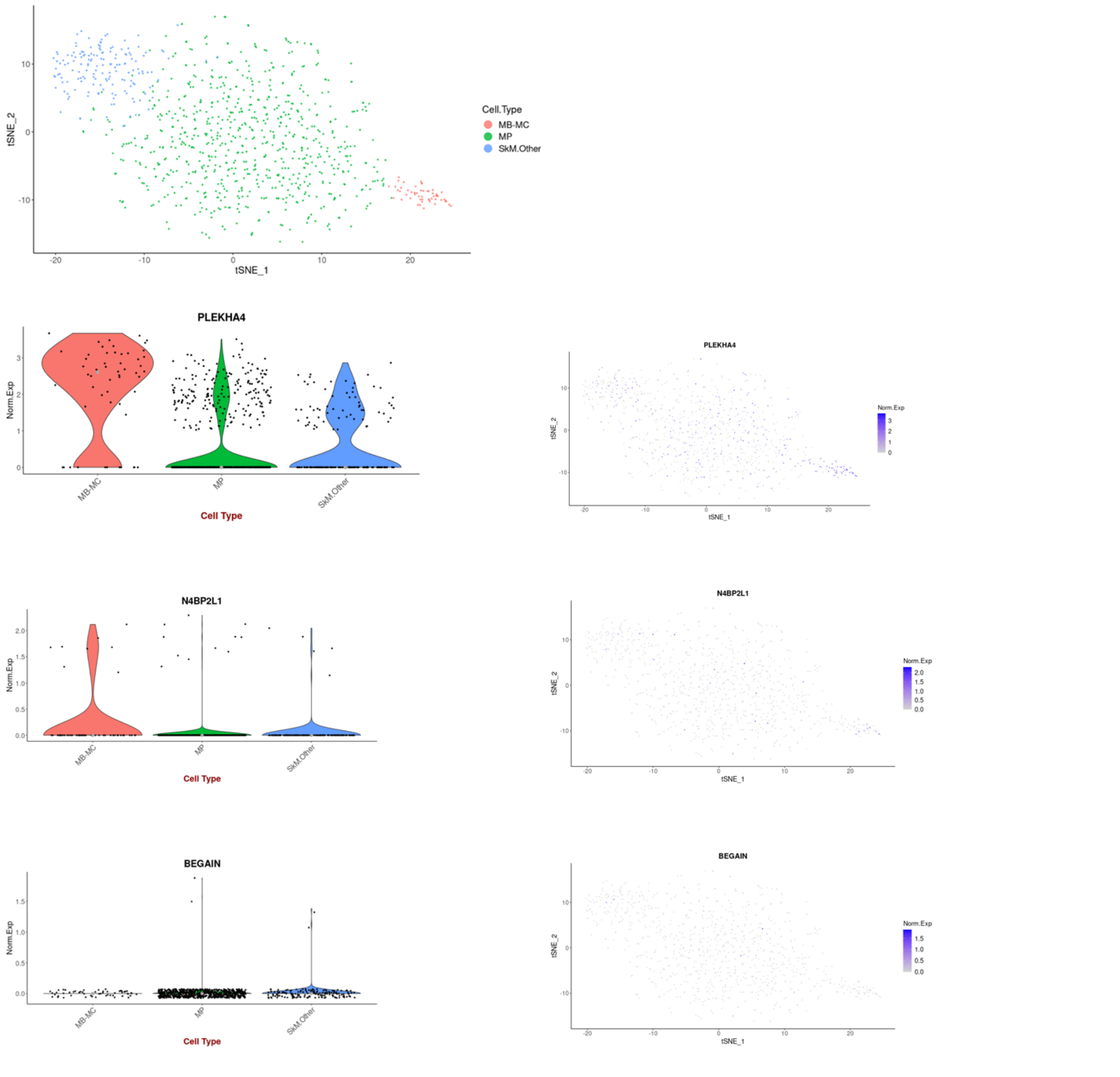

Panel.3. Juvenile

Juvenile Myogenic Subset

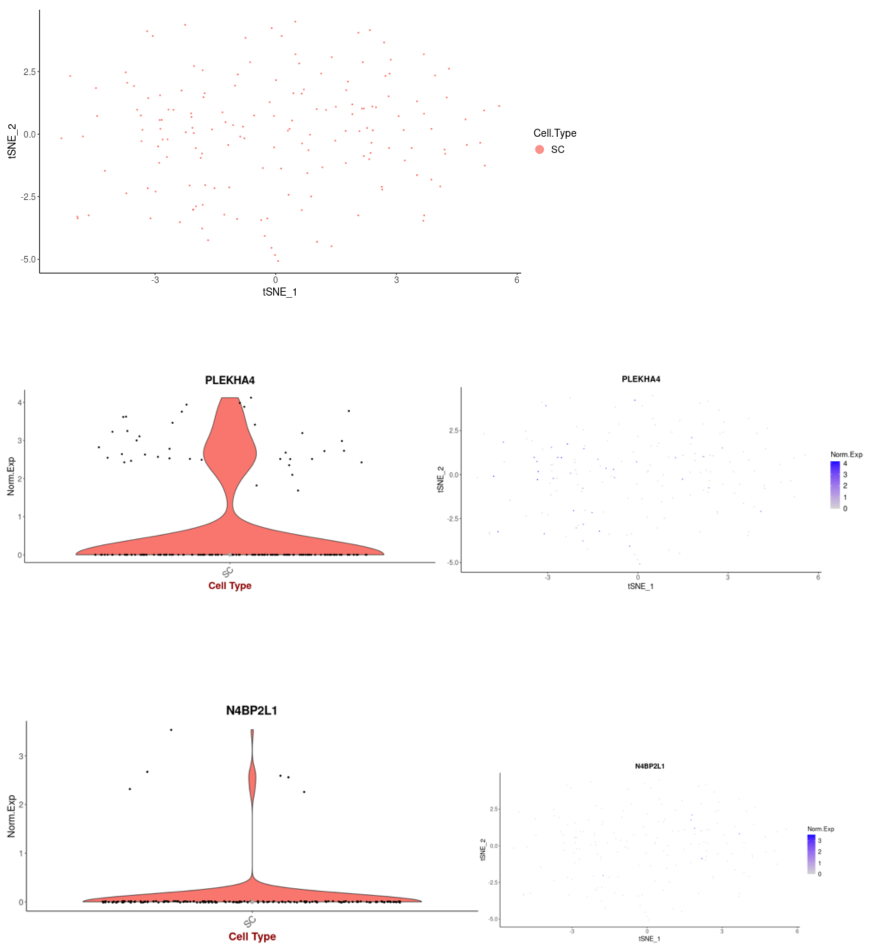

Juvenile Hindlimb Muscle

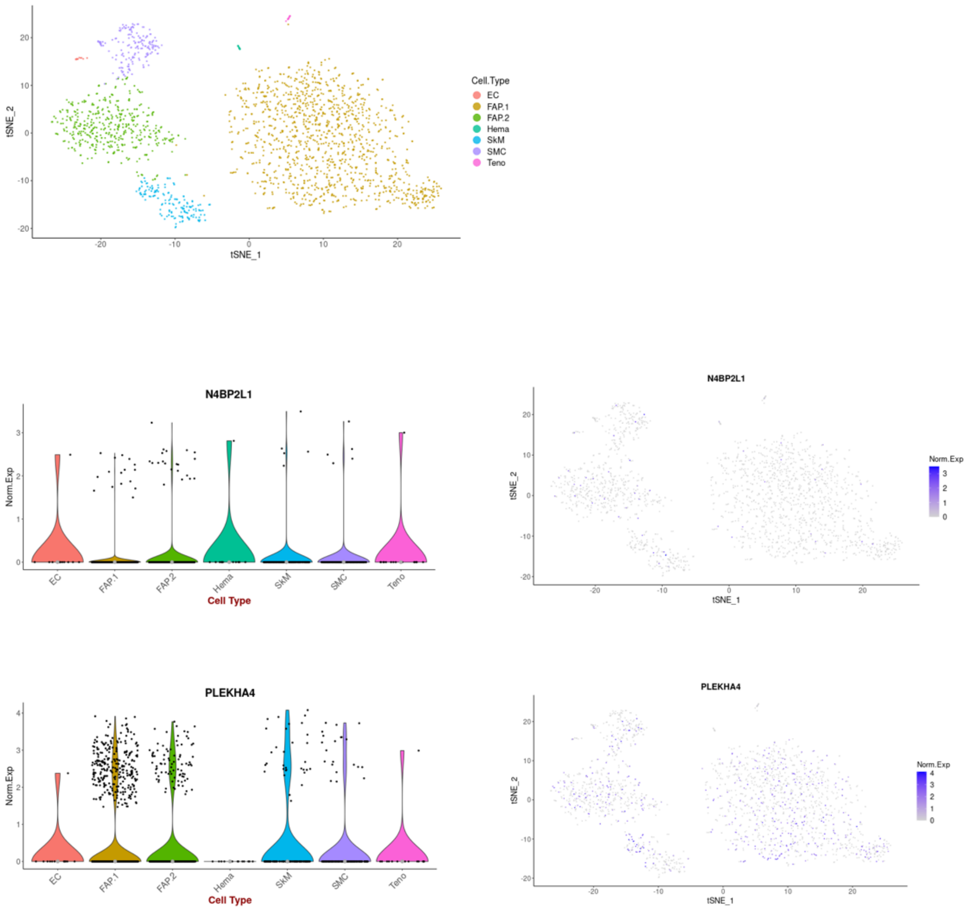

Abbreviations:

SKM: skeletal muscle; RBC: red blood cells; Limb.Mesen: limb mesenchymal progenitors; WBC: white blood cells; EC: endothelial cells; MP: myogenic progenitor; Chondro: chondrogenic cells; PreChondro: prechondrogenic cells, MB-MC: myoblasts-myocytes (MBs-MCs); Dermal: dermal fibroblasts and progenitors; Teno: tenogenic cells; SMC: smooth muscle cells; SC: postnatal satellite cells; FAP: fibro-adipogenic progenitors; Hema: hematopoietic lineages; EC-Hema.

Panel.4. Adult

Adult Hindlimb Muscle

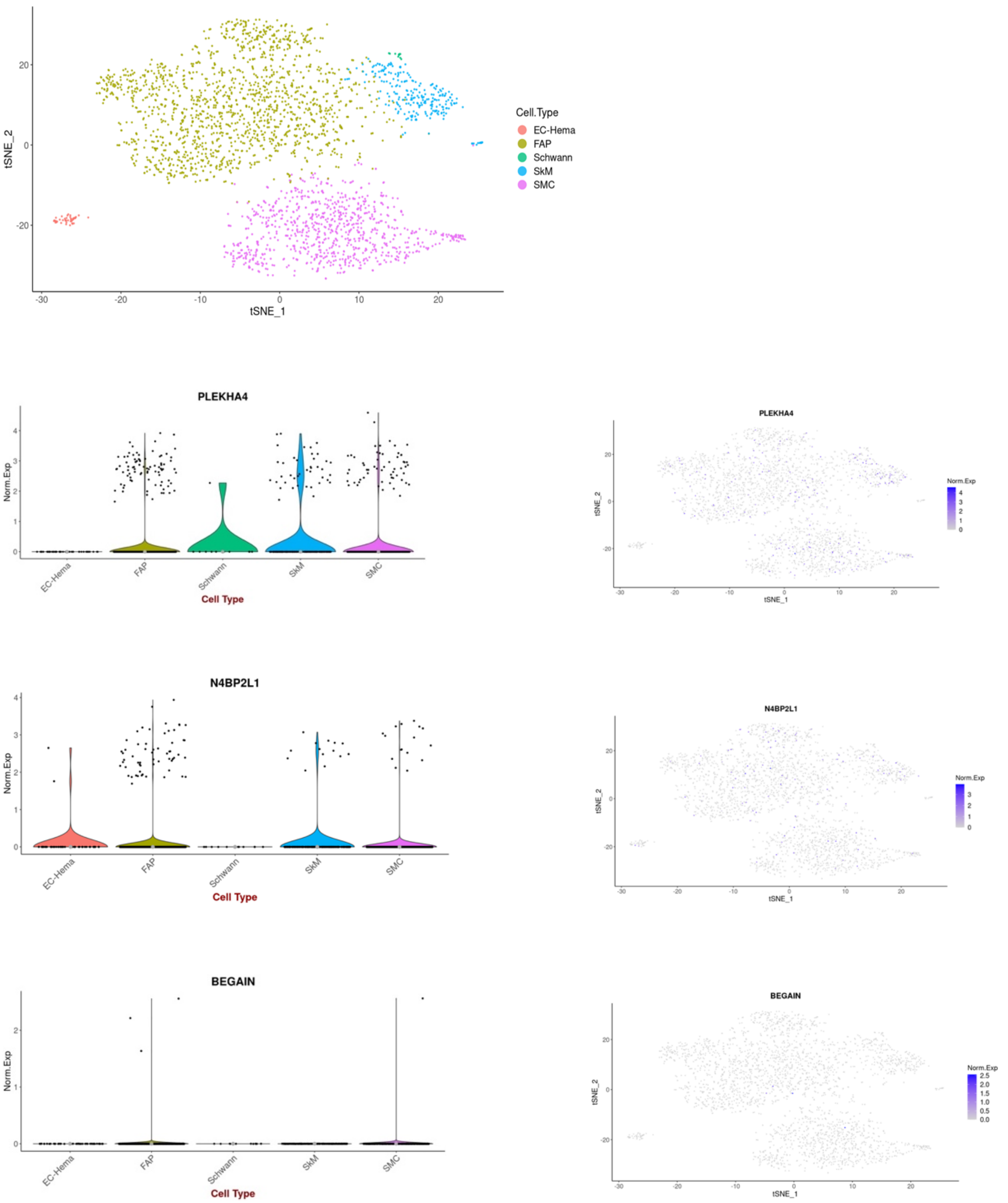

Adult Myogenic Subset

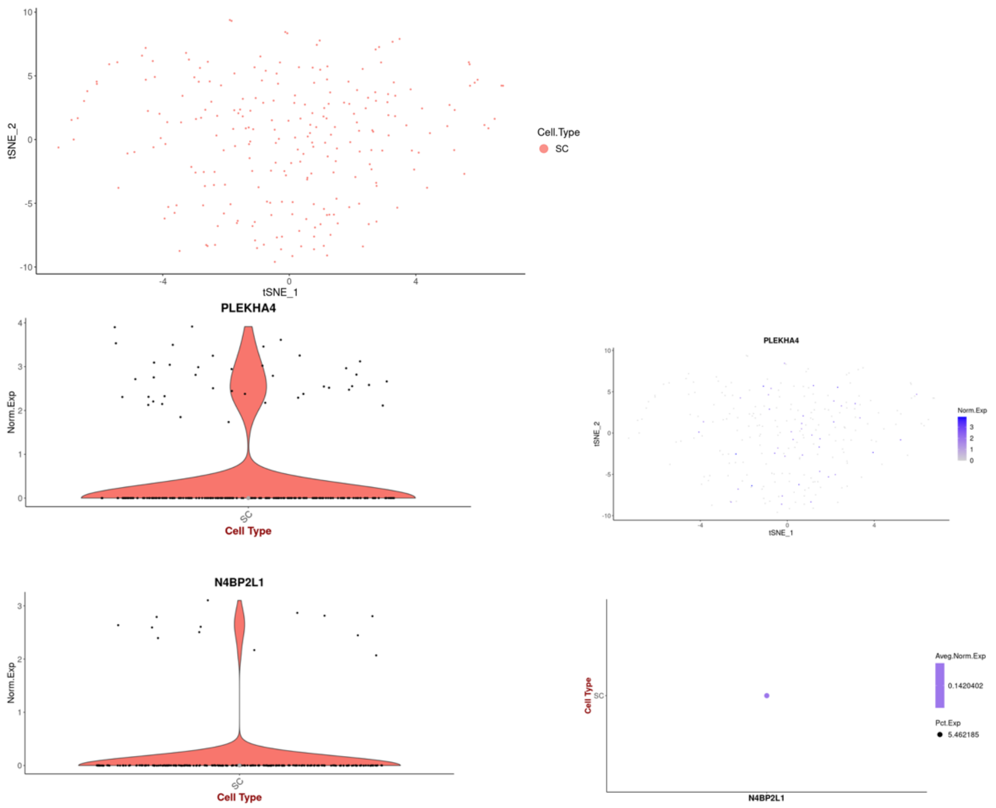

Supplement: Supplementary file 1 [file DataSheet1.zip › Supplementary files/Supplementary Figure S3.pdf]
