# Supplementary material for: Involvement of N4BP2L1, PLEKHA4, and BEGAIN genes in breast cancer and muscle cell development
Source: Front Cell Dev Biol. 2024 May 24;12:1295403. doi: 10.3389/fcell.2024.1295403 (PMC11163233; doi:10.3389/fcell.2024.1295403)
Supplement: Supplementary file 1 [file DataSheet1.zip › Supplementary files/Supplementary Figure S1.pdf]

Supplementary Figure S1. *N4BP2L1*, *PLEKHA4*, and *BEGAIN* expression in TCGA breast cancer based on different patient's statuses

Panel 1. *N4BP2L1*, *PLEKHA4*, and *BEGAIN* expression in breast cancer based on the individual cancer stage.

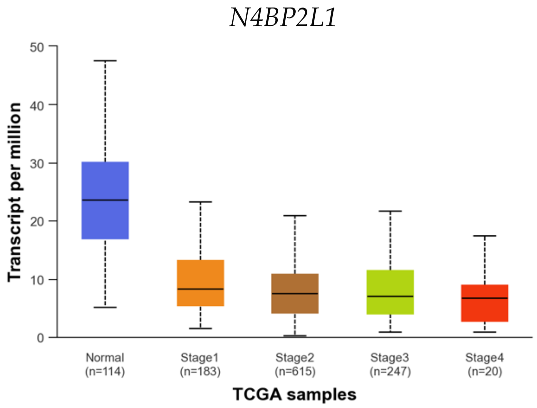

Comparison	#Statistical significance
Normal-vs-Stage 1	<1E-12
Normal-vs-Stage 2	<1E-12
Normal-vs-Stage 3	<1E-12
Normal-vs-Stage 4	1.62E-12
Stage 1-vs- Stage 2	6.46E-03
Stage 1-vs- Stage 4	1.57E-02

Only significant differential expression is given.

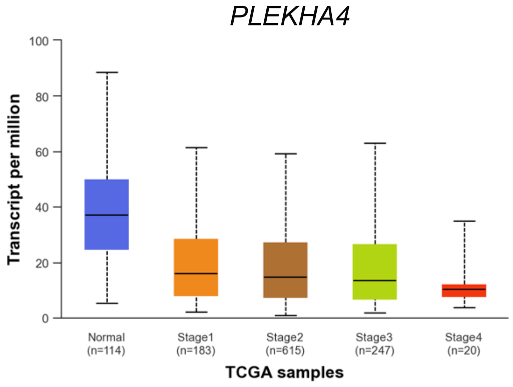

Comparison	#Statistical significance
Normal-vs-Stage 1	6.45E-14
Normal-vs-Stage 2	1.11E-16
Normal-vs-Stage 3	1.28E-14
Normal -vs-Stage4	1.883E-06

Only significant differential expression is given.

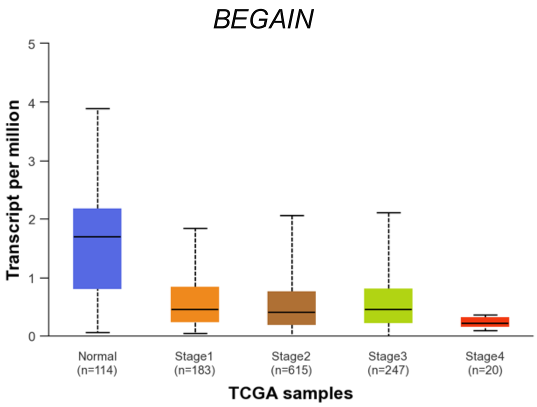

Comparison	#Statistical significance
Normal-vs-Stage 1	1.415570E-03
Normal-vs-Stage 2	4.87E-10
Normal-vs-Stage 3	1.63E-07
Normal-vs-Stage 4	3.417E-10
Stage1-vs-Stage4	6.63E-04
Stage2-vs-Stage4	3.66E-03
Stage3-vs-Stage4	2.18E-03

Only significant differential expression is given.

Panel 2. *N4BP2L1*, *PLEKHA4*, and *BEGAIN* expression in breast cancer based on three subclasses (luminal, HER2 positive, and Triple negative).

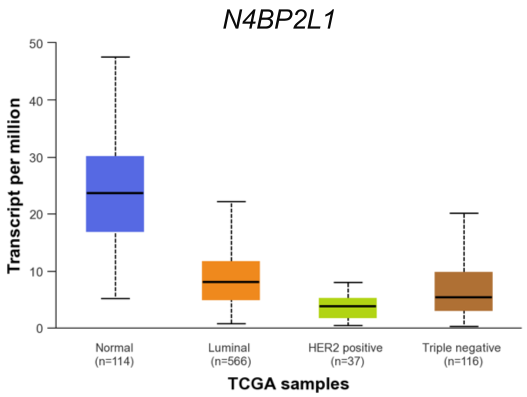

Comparison	#Statistical significance
Normal-vs-Luminal	<1E-12
Normal-vs-HER2 Positive	1.62E-12
Normal-vs-TNBC	<1E-12
Luminal-vs-HER2 Positive	3.66E-11
Luminal-vs-TNBC	2.82E-04
HER2 Positive-vs-TNBC	2.2E-04

Only significant differential expression is given

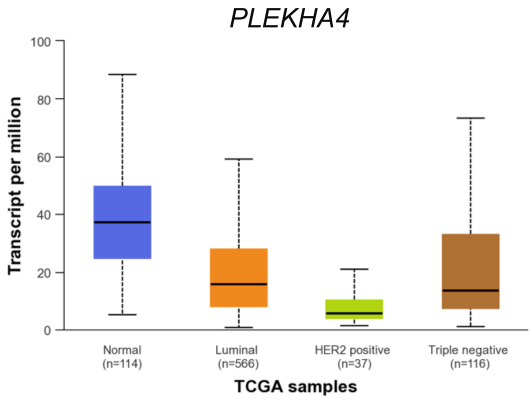

Comparison	#Statistical significance
N-vs-Luminal	1.62E-12
N-vs-HER2 Positive	1.62E-12
N-vs-TNBC	5.7E-10
Luminal-vs-HER2 Positive	1.01E-06
HER2 Positive-vs-TNBC	2.083E-05

Only significant differential expression is given

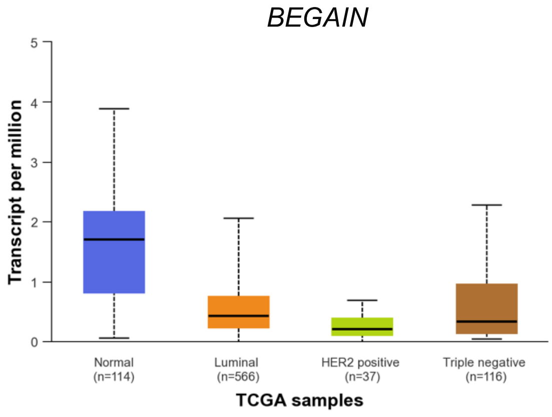

Comparison	#Statistical significance
N-vs-Luminal	1.49E-08
N-vs-HER2 Positive	4.33E-15
N-vs-TNBC	1.1E-03
Luminal-vs-HER2 Positive	1.86E-05
HER2 Positive-vs-TNBC	1.04E-03

Only significant differential expression is given

Panel 3. *N4BP2L1*, *PLEKHA4*, and *BEGAIN* expression in breast cancer based on major subclasses including TNBC.

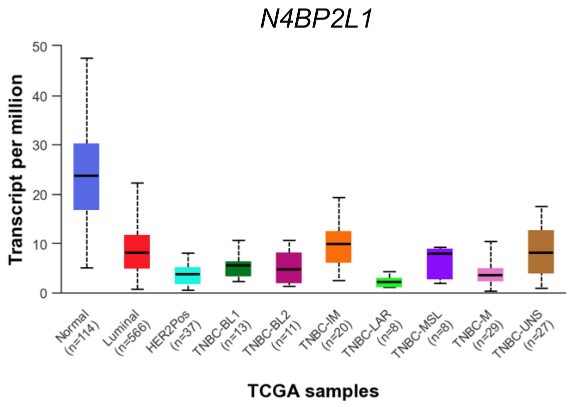

Comparison	#Statistical significance	Comparison	Statistical significance
Normal-vs-Luminal	<1E-12	HER2Pos-vs-TNBC-IM	2.21E-06
Normal-vs-HER2Pos	1.62E-12	HER2Pos-vs-TNBC-LAR	7.14E-03
Normal-vs-TNBC-BL1	1.62E-12	HER2Pos-vs-TNBC-UNS	1.94E-03
Normal-vs-TNBC-BL2	7.46E-14	TNBC-BL1-vs-TNBC-IM	1.93E-02
Normal-vs-TNBC-IM	1.62E-12	TNBC-BL1-vs-TNBC-LAR	1.66E-03
Normal-vs-TNBC-LAR	<1E-12	TNBC-BL2-vs-TNBC-IM	1.11E-02
Normal-vs-TNBC-MSL	1.72E-06	TNBC-BL2-vs-TNBC-LAR	1.99E-02
Normal-vs-TNBC-M	1.62E-12	TNBC-BL2-vs-TNBC-UNS	2.82E-02
Normal-vs-TNBC-UNS	7.21E-13	TNBC-IM-vs-TNBC-LAR	2.97E-07
Luminal-vs-HER2Pos	3.66E-11	TNBC-IM-vs-TNBC-M	8.56E-03
Luminal-vs-TNBC-BL1	3.57E-02	TNBC-LAR-vs-TNBC-MSL	4.51E-02
Luminal-vs-TNBC-BL2	1.86E-02	TNBC-LAR-vs-TNBC-M	2.04E-03
Luminal-vs-TNBC-LAR	7.22E-09	TNBC-LAR-vs-TNBC-UNS	5.64E-05
Luminal-vs-TNBC-M	5.96E-04	TNBC-M-vs-TNBC-UNS	3.19E-02

Only significant differential expression is given.

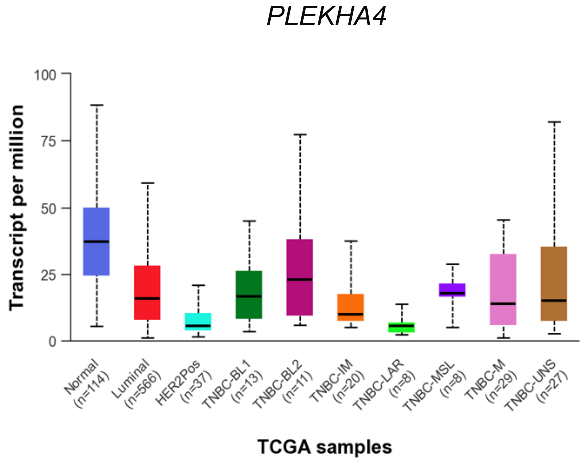

Comparison	#Statistical significance	Comparison	Statistical significance
Normal-vs-Luminal	1.62E-12	Luminal-vs-HER2Pos	1.01E-06
Normal-vs-HER2Pos	1.62E-12	HER2Pos-vs-TNBC-BL1	3.71E-02
Normal-vs-TNBC-BL1	3.59E-02	HER2Pos-vs-TNBC-BL2	3.36E-02
Normal-vs-TNBC-IM	4.40E-06	HER2Pos-vs-TNBC-MSL	1.76E-02
Normal-vs-TNBC-LAR	4.22E-05	HER2Pos-vs-TNBC-M	7.56E-03
Normal-vs-TNBC-MSL	1.05E-02	HER2Pos-vs-TNBC-UNS	4.98E-03
Normal-vs-TNBC-M	1.42E-06		

Only significant differential expression is given.

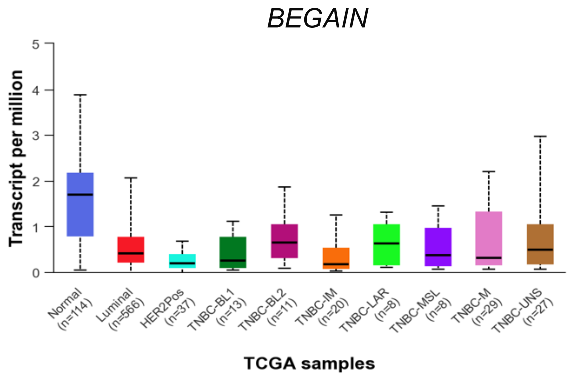

Comparison	#Statistical significance	Comparison	Statistical significance
Normal-vs-Luminal	1.49E-08	Luminal-vs-TNBC-IM	3.96E-03
Normal-vs-HER2Pos	4.33E-15	HER2Pos-vs-TNBC-M	3.22E-02
Normal-vs-TNBC-BL1	8.15E-08	HER2Pos-vs-TNBC-UNS	1.29E-02
Normal-vs-TNBC-IM	8.27E-09	TNBC-BL1-vs-TNBC-M	4.04E-02
Normal-vs-TNBC-LAR	1.81E-04	TNBC-BL1-vs-TNBC-UNS	2.16E-02
Luminal-vs-HER2Pos	1.86E-05	TNBC-IM-vs-TNBC-M	4.43E-02
Luminal-vs-TNBC-BL1	3.8E-03	TNBC-IM-vs-TNBC-UNS	2.56E-02

Only significant differential expression is given.

Panel 4. *N4BP2L1*, *PLEKHA4*, and *BEGAIN* expression in breast cancer based on histologic subtypes

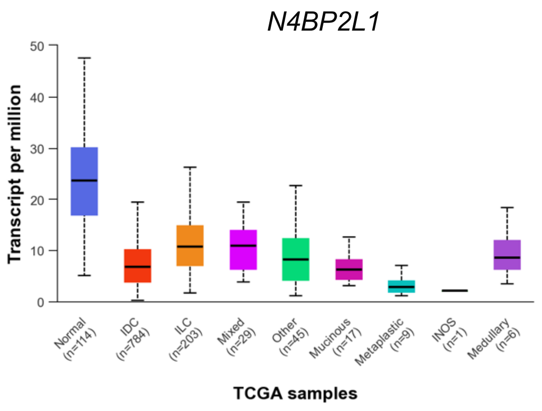

Comparison	#Statistical significance	Comparison	Statistical significance
Normal-vs-IDC	1.62E-12	ILC-vs-Other	5.6E-03
Normal-vs-ILC	1.62E-12	ILC-vs-Mucinous	1.59E-05
Normal-vs-Mixed	4.46E-05	ILC-vs-Metaplastic	4.35E-05
Normal-vs-Other	1.62E-12	Mixed-vs-Mucinous	3.01E-02
Normal-vs-Mucinous	1.62E-12	Mixed-vs-Metaplastic	2.14E-03
Normal-vs-Metaplastic	1.71E-12	Other-vs-Metaplastic	1.29E-02
Normal-vs-Medullary	1.97E-04	Mucinous-vs-Metaplastic	2.79E-02
IDC-vs-ILC	2.998E-15	Metaplastic-vs-Medullary	2.18E-02

Only significant differential expression is given.

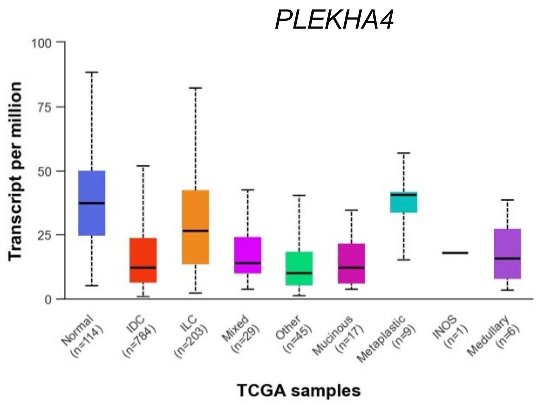

Comparison	#Statistical significance	Comparison	Statistical significance
Normal-vs-IDC	<1E-12	ILC-vs-Mixed	9.78E-07
Normal-vs-ILC	1.14E-03	ILC-vs-Other	2.09E-07
Normal-vs-Mixed	1.69E-11	ILC-vs-Mucinous	9.18E-03
Normal-vs-Other	2.45E-11	Mixed-vs-Metaplastic	1.560E-02
Normal-vs-Mucinous	2.2E-05	Other-vs-Metaplastic	3.84E-04
Normal-vs-Medullary	1.13E-02	Mucinous-vs-Metaplastic	6.22E-03
IDC-vs-ILC	4.88E-15		
IDC-vs-Metaplastic	2.570E-04		

Only significant differential expression is given.

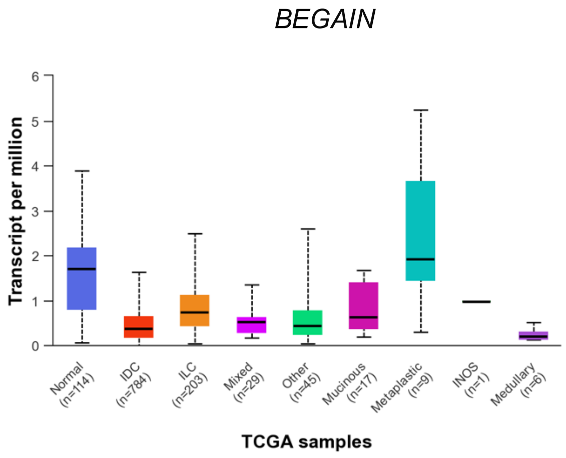

Comparison	#Statistical significance
Normal-vs-IDC	3.46E-14
Normal-vs-ILC	4.3E-02
Normal-vs-Mixed	1.34E-02
IDC-vs-ILC	7.87E-05
IDC-vs-Other	1.55E-02
IDC-vs-Metaplastic	2.51E-02
ILC-vs-Metaplastic	5.96E-03
Mixed-vs-Metaplastic	3.69E-02

Only significant differential expression is given.

Panel 5. *N4BP2L1*, *PLEKHA4*, and *BEGAIN* expression in breast cancer based on nodal metastasis status

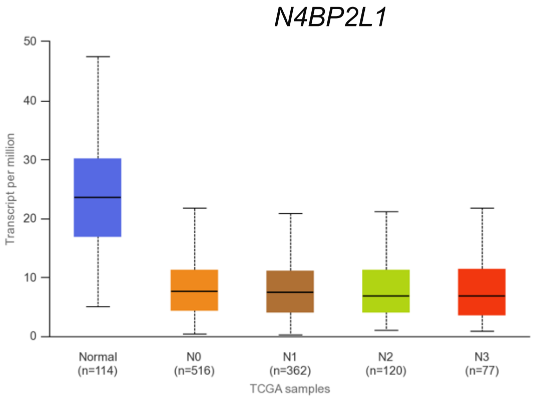

Comparison	#Statistical significance
Normal-vs-N0	<1E-12
Normal-vs-N1	<1E-12
Normal-vs-N2	1.62E-12
Normal-vs-N3	<1E-12

Only significant differential expression is given.

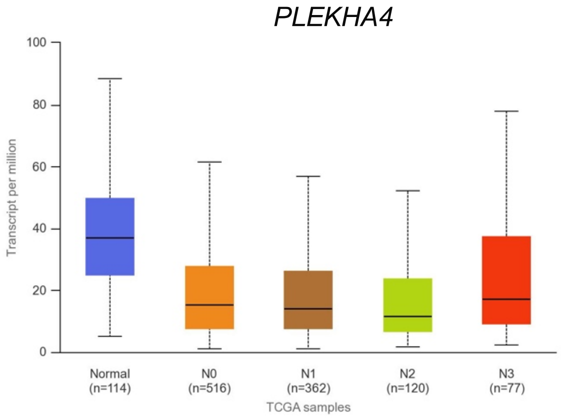

Comparison	#Statistical significance
Normal-vs-N0	1.62E-12
Normal-vs-N1	1.62E-12
Normal-vs-N2	<1E-12
Normal-vs-N3	3.81E-05
N1-vs-N3	3.75E-02
N2-vs-N3	1.67E-02

Only significant differential expression is given.

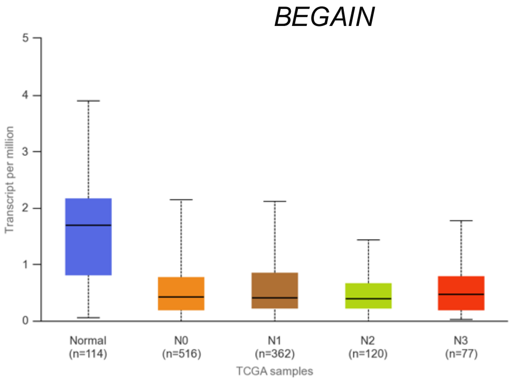

Comparison	#Statistical significance
Normal-vs-N0	4.89E-08
Normal-vs-N1	3.39E-07
Normal-vs-N2	1.68E-12
Normal-vs-N3	8.23E-05
N0-vs-N2	2.1E-03
N1-vs-N2	1.25E-02
N2-vs-N3	3.68E-02

Only significant differential expression is given.
